# Supplementary material for: Impacts of agricultural machine renting on cereal crop productivity and commercialization in West Gojjam Zone, Ethiopia
Source: PLoS One. 2025 Oct 27;20(10):e0300831. doi: 10.1371/journal.pone.0300831 (PMC12558508; doi:10.1371/journal.pone.0300831)
Supplement: S1 Data — (DOCX) [file pone.0300831.s001.docx]

Supporting data for impacts of agricultural machine renting on cereal crop productivity and commercialization in west gojjam zone, ethiopia".

Table 1 Demographic, socio-economic and institutional characteristics for continuous variable

| Variable | Obs | Mean | Std. Dev. | Min | Max |
| --- | --- | --- | --- | --- | --- |
| Age of hh | 400 | 44.75 | 9.64 | 24 | 70 |
| Education level | 400 | 3.78 | 3.08 | 0 | 12 |
| Dependence ratio | 400 | 0.48 | 0.39 | 0 | 2 |
| Farming experience | 400 | 20.05 | 9.68 | 1 | 48 |
| Family size | 400 | 6.12 | 1.69 | 1 | 12 |
| distance to market | 400 | 3.19 | 2.39 | 0.1 | 16 |
| Distance to main road | 400 | 2.37 | 1.54 | 0.1 | 7 |
| Distance to DA | 400 | 2.29 | 1.36 | .15 | 8 |
| Extension visit | 400 | 11.03 | 6.21 | 1 | 30 |
| Total land | 400 | 2.14 | 0.92 | 0.25 | 5 |
| Ownership of oxen | 400 | 2.39 | 0.97 | 0 | 6 |
| TLU | 400 | 4.00 | 2.06 | 0 | 12 |
| Own land | 400 | 1.44 | 0.77 | 0 | 4 |

Table 2. Demographic, socio-economic and institutional characteristics for categorical variable

| Variable | Description | No of households(n=400) | Percent |
| --- | --- | --- | --- |
| Sex of household head | Male | 379 | 94.75 |
|  | Female | 21 | 5.25 |
| Position social participation | Yes | 195 | 48.75 |
| Credit use | Yes | 173 | 43.25 |
| Membership of cooperative | Yes | 317 | 79.25 |
| Off -farm participation | Yes | 131 | 32.75 |
| Machine renting | Yes | 192 | 48 |
| access information EA | Yes | 282 | 70.5 |

| Table 3. **Common support** | | | |  | | | | |  | | | |  |
| --- | --- | --- | --- | --- | --- | --- | --- | --- | --- | --- | --- | --- | --- |
| psmatch2: | | | | psmatch2:common | | | | |  | | | |  |
| Treatment | | | | | Support | | | | | |  |  |  |
| assignment |  | | Off suppo | | |  | | 0n suppor | | Total | |  |  |
| Untreated | | 14 | | | | | 194 | | | | 208 | | |
| Treated | | 14 | | | | | 178 | | | | 192 | | |
| Total | | 21 | | | | | 372 | | | | 400 | | |

Table 4. Commercialization, Technical efficiency and Total factor productivity indices

| HH ID | TFP | TE | COIM | HH ID | TFP | TE | COIM |
| --- | --- | --- | --- | --- | --- | --- | --- |
| 1 | 3.314883 | 0.916087 | 0.604396 | 36 | 4.420247 | 0.934483 | 0.642857 |
| 2 | 3.265252 | 0.88335 | 0.491803 | 37 | 4.037621 | 0.943491 | 0.61017 |
| 3 | 3.081469 | 0.925374 | 0.666667 | 38 | 2.208696 | 0.905104 | 0.587302 |
| 4 | 3.440367 | 0.891414 | 0.5 | 39 | 2.491271 | 0.872582 | 0.6 |
| 5 | 2.956638 | 0.871048 | 0.587629 | 40 | 4.398241 | 0.945318 | 0.66 |
| 6 | 3.913043 | 0.812401 | 0.56 | 41 | 2.835788 | 0.934683 | 0.537634 |
| 7 | 2.518818 | 0.916558 | 0.557377 | 42 | 3.65774 | 0.908858 | 0.55 |
| 8 | 1.934741 | 0.902133 | 0.645833 | 43 | 2.452924 | 0.90539 | 0.6 |
| 9 | 3.936671 | 0.879911 | 0.304348 | 44 | 2.676375 | 0.898668 | 0.58 |
| 10 | 3.004197 | 0.872575 | 0.564516 | 45 | 3.471196 | 0.925908 | 0.469388 |
| 11 | 3.166773 | 0.957589 | 0.644445 | 46 | 3.969735 | 0.914061 | 0.621212 |
| 12 | 3.107817 | 0.924469 | 0.576271 | 47 | 2.861307 | 0.895231 | 0.495413 |
| 13 | 3.867105 | 0.860157 | 0.621277 | 48 | 3.404743 | 0.899068 | 0.594595 |
| 14 | 3.187682 | 0.800339 | 0.642336 | 49 | 2.6918 | 0.901705 | 0.663044 |
| 15 | 3.038212 | 0.728839 | 0.615385 | 50 | 4.656374 | 0.938142 | 0.655172 |
| 16 | 2.822423 | 0.901752 | 0.528571 | 51 | 3.301627 | 0.861791 | 0.649123 |
| 17 | 2.959502 | 0.75935 | 0.466667 | 52 | 2.765547 | 0.877734 | 0.448598 |
| 18 | 1.528004 | 0.837526 | 0.56383 | 53 | 2.769367 | 0.734188 | 0.575163 |
| 19 | 2.733333 | 0.752122 | 0.6375 | 54 | 3.799373 | 0.909737 | 0.72619 |
| 20 | 3.44086 | 0.900907 | 0.525 | 55 | 3.919479 | 0.880358 | 0.566038 |
| 21 | 3.064524 | 0.78401 | 0.633588 | 56 | 3.689655 | 0.799969 | 0.487805 |
| 22 | 3.710097 | 0.858278 | 0.615385 | 57 | 3.897893 | 0.929007 | 0.413044 |
| 23 | 3.262189 | 0.819641 | 0.544218 | 58 | 3.127728 | 0.90464 | 0.616667 |
| 24 | 3.525035 | 0.742353 | 0.631579 | 59 | 3.16886 | 0.934491 | 0.411765 |
| 25 | 3.083916 | 0.914213 | 0.714286 | 60 | 3.905109 | 0.919786 | 0.622222 |
| 26 | 2.467126 | 0.681855 | 0.404762 | 61 | 2.348263 | 0.658772 | 0.538462 |
| 27 | 2.933184 | 0.876528 | 0.515625 | 62 | 4.098551 | 0.91366 | 0.5 |
| 28 | 2.646557 | 0.921236 | 0.444444 | 63 | 3.056686 | 0.731668 | 0.5 |
| 29 | 2.976607 | 0.853201 | 0.514563 | 64 | 3.957794 | 0.93555 | 0.64 |
| 30 | 3.961562 | 0.909865 | 0.52381 | 65 | 2.932193 | 0.852737 | 0.611765 |
| 31 | 4.03437 | 0.931357 | 0.5 | 66 | 2.508234 | 0.939693 | 0.688889 |
| 32 | 3.465812 | 0.924566 | 0.725352 | 67 | 3.722994 | 0.929568 | 0.666667 |
| 33 | 4.017889 | 0.916027 | 0.62 | 68 | 3.491213 | 0.92508 | 0.683673 |
| 34 | 3.802334 | 0.883318 | 0.648649 | 69 | 3.869416 | 0.914597 | 0.559633 |
| 35 | 3.552463 | 0.918672 | 0.733333 | 70 | 2.9552 | 0.9389 | 0.763359 |

| HHID | TFP | TE | COIM | HHID | TFP | TE | COIM |
| --- | --- | --- | --- | --- | --- | --- | --- |
| 71 | 2.46047 | 0.938978 | 0.594595 | 106 | 2.711817 | 0.804642 | 0.45283 |
| 72 | 2.186186 | 0.868438 | 0.619883 | 107 | 2.977229 | 0.94903 | 0.626087 |
| 73 | 4.146498 | 0.91381 | 0.532609 | 108 | 3.286713 | 0.897967 | 0.424242 |
| 74 | 3.206569 | 0.884794 | 0.5 | 109 | 3.763174 | 0.889195 | 0.461539 |
| 75 | 3.414634 | 0.937775 | 0.526316 | 110 | 2.54468 | 0.887575 | 0.643836 |
| 76 | 2.755005 | 0.740413 | 0.5 | 111 | 2.923424 | 0.881888 | 0.597015 |
| 77 | 3.576361 | 0.9131 | 0.563218 | 112 | 3.08751 | 0.903463 | 0.461539 |
| 78 | 3.411223 | 0.91046 | 0.584906 | 113 | 2.616418 | 0.893313 | 0.569767 |
| 79 | 3.605252 | 0.925575 | 0.507692 | 114 | 3.403398 | 0.899092 | 0.5 |
| 80 | 3.283828 | 0.940856 | 0.527027 | 115 | 2.918919 | 0.867964 | 0.513889 |
| 81 | 1.570439 | 0.277492 | 0.5 | 116 | 2.983672 | 0.838371 | 0.5 |
| 82 | 1.071055 | 0.783051 | 0.392157 | 117 | 3.828077 | 0.906843 | 0.632768 |
| 83 | 1.988654 | 0.906954 | 0.56579 | 118 | 3.823125 | 0.909071 | 0.524753 |
| 84 | 2.366774 | 0.851341 | 0.432432 | 119 | 1.903003 | 0.876522 | 0.509091 |
| 85 | 2.914881 | 0.92819 | 0.415094 | 120 | 3.0951 | 0.848573 | 0.45946 |
| 86 | 3.597527 | 0.922268 | 0.65 | 121 | 2.919107 | 0.872254 | 0.581818 |
| 87 | 2.158631 | 0.669453 | 0.416667 | 122 | 2.585441 | 0.85219 | 0.619048 |
| 88 | 3.052593 | 0.854735 | 0.5625 | 123 | 2.129843 | 0.765272 | 0.566667 |
| 89 | 3.092511 | 0.901619 | 0.540541 | 124 | 4.539632 | 0.943413 | 0.597403 |
| 90 | 3.160041 | 0.897064 | 0.514286 | 125 | 3.610901 | 0.941145 | 0.616438 |
| 91 | 3.700234 | 0.901492 | 0.65625 | 126 | 3.433923 | 0.850523 | 0.482412 |
| 92 | 3.664444 | 0.912738 | 0.556962 | 127 | 4.114866 | 0.921261 | 0.73494 |
| 93 | 1.457627 | 0.797733 | 0.457143 | 128 | 2.562529 | 0.857171 | 0.694118 |
| 94 | 2.800337 | 0.748327 | 0.468085 | 129 | 3.762221 | 0.9234 | 0.608696 |
| 95 | 2.735143 | 0.828966 | 0.54 | 130 | 3.36243 | 0.842081 | 0.517241 |
| 96 | 3.103113 | 0.923646 | 0.558824 | 131 | 2.124164 | 0.885183 | 0.663044 |
| 97 | 2.434642 | 0.65078 | 0.547009 | 132 | 2.080434 | 0.829945 | 0.55 |
| 98 | 2.797439 | 0.701593 | 0.582857 | 133 | 3.522431 | 0.875621 | 0.442308 |
| 99 | 2.898135 | 0.725381 | 0.62037 | 134 | 4.46349 | 0.945543 | 0.651515 |
| 100 | 2.54551 | 0.805379 | 0.526316 | 135 | 2.166831 | 0.696529 | 0.425532 |
| 101 | 2.681695 | 0.749801 | 0.700389 | 136 | 3.416831 | 0.937162 | 0.66129 |
| 102 | 2.829975 | 0.457747 | 0.648649 | 137 | 2.981525 | 0.748731 | 0.555556 |
| 103 | 2.965446 | 0.831321 | 0.714286 | 138 | 3.616601 | 0.920704 | 0.577778 |
| 104 | 2.475192 | 0.758059 | 0.507463 | 139 | 3.75434 | 0.922443 | 0.622222 |
| 105 | 2.893557 | 0.800236 | 0.631579 | 140 | 3.342012 | 0.846915 | 0.58046 |

| HH ID | TFP | TE | COIM | HH ID | TFP | TE | COIM |
| --- | --- | --- | --- | --- | --- | --- | --- |
| 141 | 3.479532 | 0.917418 | 0.686567 | 176 | 3.867444 | 0.884477 | 0.744681 |
| 142 | 3.475707 | 0.86452 | 0.691589 | 177 | 3.398176 | 0.903178 | 0.712329 |
| 143 | 3.936096 | 0.922064 | 0.583333 | 178 | 3.102041 | 0.877403 | 0.707692 |
| 144 | 3.696581 | 0.932731 | 0.5 | 179 | 3.02185 | 0.842234 | 0.614458 |
| 145 | 1.434055 | 0.220551 | 0 | 180 | 2.808151 | 0.807057 | 0.596491 |
| 146 | 1.746433 | 0.88573 | 0.606061 | 181 | 2.536469 | 0.853436 | 0.563636 |
| 147 | 2.976099 | 0.901109 | 0.528571 | 182 | 3.081851 | 0.62785 | 0.30303 |
| 148 | 4.015584 | 0.900826 | 0.675676 | 183 | 4.277309 | 0.914273 | 0.605634 |
| 149 | 3.473804 | 0.906648 | 0.708333 | 184 | 1.975651 | 0.9032 | 0.466667 |
| 150 | 2.785334 | 0.849174 | 0.660714 | 185 | 1.988851 | 0.927853 | 0.478723 |
| 151 | 2.75672 | 0.884334 | 0.666667 | 186 | 2.541993 | 0.801779 | 0.454546 |
| 152 | 3.288982 | 0.90012 | 0.708333 | 187 | 1.566592 | 0.743576 | 0.395349 |
| 153 | 3.641853 | 0.891166 | 0.679012 | 188 | 3.608956 | 0.869339 | 0.5 |
| 154 | 3.182011 | 0.861782 | 0.652174 | 189 | 2.255342 | 0.511467 | 0.457143 |
| 155 | 2.635963 | 0.576332 | 0.409091 | 190 | 1.583561 | 0.464734 | 0.37037 |
| 156 | 2.961837 | 0.890128 | 0.592593 | 191 | 1.53587 | 0.35778 | 0 |
| 157 | 3.570185 | 0.90062 | 0.744898 | 192 | 1.711984 | 0.670826 | 0.294118 |
| 158 | 4.498106 | 0.914345 | 0.5 | 193 | 1.429551 | 0.485728 | 0.511628 |
| 159 | 2.862767 | 0.894097 | 0.555556 | 194 | 2.041365 | 0.503087 | 0.225806 |
| 160 | 2.346106 | 0.783364 | 0.447368 | 193 | 3.099025 | 0.866939 | 0.473684 |
| 161 | 2.977064 | 0.907756 | 0.563636 | 196 | 3.292573 | 0.858548 | 0.613636 |
| 162 | 3.219844 | 0.90111 | 0.654545 | 197 | 2.373364 | 0.597299 | 0.428571 |
| 163 | 2.921707 | 0.835407 | 0.704918 | 198 | 3.138212 | 0.785351 | 0.5 |
| 164 | 2.261966 | 0.612739 | 0.891892 | 199 | 3.439163 | 0.850561 | 0.421053 |
| 165 | 2.708631 | 0.78602 | 0.578947 | 200 | 3.697019 | 0.882043 | 0.535714 |
| 166 | 2.779884 | 0.892272 | 0.6875 | 201 | 3.188088 | 0.794568 | 0.5 |
| 167 | 2.674358 | 0.870979 | 0.630769 | 202 | 2.693224 | 0.69345 | 0.514286 |
| 168 | 3.916044 | 0.919122 | 0.75 | 203 | 2.681255 | 0.941357 | 0.554546 |
| 169 | 1.978085 | 0.881365 | 0.555556 | 204 | 3.794306 | 0.842587 | 0.407407 |
| 170 | 1.949965 | 0.798098 | 0.567164 | 205 | 3.798208 | 0.914063 | 0.444444 |
| 171 | 2.424661 | 0.907472 | 0.536585 | 206 | 2.751307 | 0.840979 | 0.380952 |
| 172 | 3.434508 | 0.885985 | 0.641026 | 207 | 2.778367 | 0.656829 | 0.391304 |
| 173 | 3.182921 | 0.925086 | 0.705882 | 208 | 3.109369 | 0.884271 | 0.589744 |
| 174 | 3.965262 | 0.900208 | 0.702128 | 209 | 3.245645 | 0.899106 | 0.48 |
| 175 | 2.783536 | 0.856582 | 0.560976 | 210 | 3.527018 | 0.880493 | 0.539683 |

| HHID | TFP | TE | COIM | HHID | TFP | TE | COIM |
| --- | --- | --- | --- | --- | --- | --- | --- |
| 71 | 2.46047 | 0.938978 | 0.594595 | 106 | 2.711817 | 0.804642 | 0.45283 |
| 72 | 2.186186 | 0.868438 | 0.619883 | 107 | 2.977229 | 0.94903 | 0.626087 |
| 73 | 4.146498 | 0.91381 | 0.532609 | 108 | 3.286713 | 0.897967 | 0.424242 |
| 74 | 3.206569 | 0.884794 | 0.5 | 109 | 3.763174 | 0.889195 | 0.461539 |
| 75 | 3.414634 | 0.937775 | 0.526316 | 110 | 2.54468 | 0.887575 | 0.643836 |
| 76 | 2.755005 | 0.740413 | 0.5 | 111 | 2.923424 | 0.881888 | 0.597015 |
| 77 | 3.576361 | 0.9131 | 0.563218 | 112 | 3.08751 | 0.903463 | 0.461539 |
| 78 | 3.411223 | 0.91046 | 0.584906 | 113 | 2.616418 | 0.893313 | 0.569767 |
| 79 | 3.605252 | 0.925575 | 0.507692 | 114 | 3.403398 | 0.899092 | 0.5 |
| 80 | 3.283828 | 0.940856 | 0.527027 | 115 | 2.918919 | 0.867964 | 0.513889 |
| 81 | 1.570439 | 0.277492 | 0.5 | 116 | 2.983672 | 0.838371 | 0.5 |
| 82 | 1.071055 | 0.783051 | 0.392157 | 117 | 3.828077 | 0.906843 | 0.632768 |
| 83 | 1.988654 | 0.906954 | 0.56579 | 118 | 3.823125 | 0.909071 | 0.524753 |
| 84 | 2.366774 | 0.851341 | 0.432432 | 119 | 1.903003 | 0.876522 | 0.509091 |
| 85 | 2.914881 | 0.92819 | 0.415094 | 120 | 3.0951 | 0.848573 | 0.45946 |
| 86 | 3.597527 | 0.922268 | 0.65 | 121 | 2.919107 | 0.872254 | 0.581818 |
| 87 | 2.158631 | 0.669453 | 0.416667 | 122 | 2.585441 | 0.85219 | 0.619048 |
| 88 | 3.052593 | 0.854735 | 0.5625 | 123 | 2.129843 | 0.765272 | 0.566667 |
| 89 | 3.092511 | 0.901619 | 0.540541 | 124 | 4.539632 | 0.943413 | 0.597403 |
| 90 | 3.160041 | 0.897064 | 0.514286 | 125 | 3.610901 | 0.941145 | 0.616438 |
| 91 | 3.700234 | 0.901492 | 0.65625 | 126 | 3.433923 | 0.850523 | 0.482412 |
| 92 | 3.664444 | 0.912738 | 0.556962 | 127 | 4.114866 | 0.921261 | 0.73494 |
| 93 | 1.457627 | 0.797733 | 0.457143 | 128 | 2.562529 | 0.857171 | 0.694118 |
| 94 | 2.800337 | 0.748327 | 0.468085 | 129 | 3.762221 | 0.9234 | 0.608696 |
| 95 | 2.735143 | 0.828966 | 0.54 | 130 | 3.36243 | 0.842081 | 0.517241 |
| 96 | 3.103113 | 0.923646 | 0.558824 | 131 | 2.124164 | 0.885183 | 0.663044 |
| 97 | 2.434642 | 0.65078 | 0.547009 | 132 | 2.080434 | 0.829945 | 0.55 |
| 98 | 2.797439 | 0.701593 | 0.582857 | 133 | 3.522431 | 0.875621 | 0.442308 |
| 99 | 2.898135 | 0.725381 | 0.62037 | 134 | 4.46349 | 0.945543 | 0.651515 |
| 100 | 2.54551 | 0.805379 | 0.526316 | 135 | 2.166831 | 0.696529 | 0.425532 |
| 101 | 2.681695 | 0.749801 | 0.700389 | 136 | 3.416831 | 0.937162 | 0.66129 |
| 102 | 2.829975 | 0.457747 | 0.648649 | 137 | 2.981525 | 0.748731 | 0.555556 |
| 103 | 2.965446 | 0.831321 | 0.714286 | 138 | 3.616601 | 0.920704 | 0.577778 |
| 104 | 2.475192 | 0.758059 | 0.507463 | 139 | 3.75434 | 0.922443 | 0.622222 |
| 105 | 2.893557 | 0.800236 | 0.631579 | 140 | 3.342012 | 0.846915 | 0.58046 |

| HH ID | TFP | TE | COIM | HH ID | TFP | TE | 0.419355 |
| --- | --- | --- | --- | --- | --- | --- | --- |
| 281 | 2.60558 | 0.685708 | 0.436893 | 316 | 2.809917 | 0.809308 | 0.305556 |
| 282 | 2.475559 | 0.638161 | 0.492064 | 317 | 2.655659 | 0.557093 | 0.405063 |
| 283 | 3.156014 | 0.764773 | 0.596154 | 318 | 2.053401 | 0.818913 | 0.371429 |
| 284 | 2.068829 | 0.631725 | 0.384615 | 319 | 1.609941 | 0.834555 | 0.307692 |
| 285 | 2.542373 | 0.798709 | 0.428571 | 320 | 1.373613 | 0.609361 | 0.525 |
| 286 | 2.249976 | 0.689746 | 0.516667 | 321 | 1.849061 | 0.658399 | 0 |
| 287 | 1.64765 | 0.669796 | 0.560976 | 322 | 2.091014 | 0.747181 | 0 |
| 288 | 2.443002 | 0.595283 | 0.305556 | 323 | 3.201581 | 0.385498 | 0.489209 |
| 289 | 3.482739 | 0.911496 | 0.54023 | 324 | 2.81671 | 0.828228 | 0.477612 |
| 290 | 3.006737 | 0.861971 | 0.47191 | 325 | 3.083925 | 0.916851 | 0.391892 |
| 291 | 2.681233 | 0.872045 | 0.518519 | 326 | 2.203074 | 0.884871 | 0.48718 |
| 292 | 2.097068 | 0.704729 | 0.491803 | 327 | 2.420888 | 0.902614 | 0.460318 |
| 293 | 2.1985 | 0.679308 | 0.473684 | 328 | 3.013819 | 0.859377 | 0.52381 |
| 294 | 2.127213 | 0.687016 | 0.387097 | 329 | 2.999072 | 0.732782 | 0.5 |
| 295 | 2.588336 | 0.860205 | 0.423729 | 330 | 2.546283 | 0.906258 | 0.453488 |
| 296 | 2.659902 | 0.605826 | 0.333333 | 331 | 3.028135 | 0.914806 | 0.578125 |
| 297 | 2.773415 | 0.785896 | 0.491228 | 332 | 2.646099 | 0.777398 | 0.419355 |
| 298 | 2.806144 | 0.745375 | 0.546875 | 333 | 1.6924 | 0.783889 | 0.434783 |
| 299 | 2.98424 | 0.907481 | 0.413793 | 334 | 3.779604 | 0.93621 | 0.468966 |
| 300 | 2.60224 | 0.752199 | 0.36 | 333 | 2.713346 | 0.760181 | 0.670732 |
| 301 | 2.998632 | 0.782627 | 0.507246 | 336 | 2.381292 | 0.831233 | 0.424242 |
| 302 | 3.662022 | 0.888359 | 0.456522 | 337 | 2.377307 | 0.760655 | 0.479452 |
| 303 | 3.296788 | 0.899253 | 0.414634 | 338 | 3.190731 | 0.852853 | 0 |
| 304 | 2.944039 | 0.85746 | 0.454546 | 349 | 2.088655 | 0.270397 | 0.461539 |
| 305 | 1.837754 | 0.719697 | 0.387755 | 350 | 2.951903 | 0.741841 | 0.45 |
| 306 | 2.379988 | 0.778684 | 0.396825 | 351 | 3.231939 | 0.817972 | 0.178571 |
| 307 | 2.507424 | 0.845665 | 0.43871 | 352 | 1.960098 | 0.82241 | 0 |
| 308 | 2.705045 | 0.832369 | 0.381356 | 353 | 1.719432 | 0.238839 | 0.5 |
| 309 | 2.386358 | 0.811835 | 0.392405 | 354 | 3.040791 | 0.806922 | 0.44 |
| 310 | 2.1637 | 0.817065 | 0.392157 | 355 | 2.720606 | 0.76049 | 0.346939 |
| 311 | 2.124921 | 0.778997 | 0.364865 | 356 | 2.054753 | 0.841079 | 0.482759 |
| 312 | 2.645785 | 0.830273 | 0.455882 | 357 | 2.975474 | 0.727592 | 0.377359 |
| 313 | 2.17491 | 0.723245 | 0.421053 | 358 | 1.635514 | 0.697627 | 0.5 |
| 314 | 3.340112 | 0.78501 | 0.533333 | 359 | 1.952614 | 0.863591 | 0.564516 |
| 315 | 1.777544 | 0.955267 | 0.473684 | 350 | 3.780343 | 0.917522 | 0.419355 |

| HHID | TFP | TE | COIM | HHID | TFP | TE | COIM |
| --- | --- | --- | --- | --- | --- | --- | --- |
| 351 | 1.756511 | 0.721271 | 0.533333 | 386 | 3.001253 | 0.683219 | 0.452381 |
| 352 | 2.471359 | 0.882659 | 0.416667 | 387 | 2.249291 | 0.569123 | 0.344828 |
| 353 | 3.905977 | 0.957076 | 0.473118 | 388 | 3.268134 | 0.906098 | 0.410959 |
| 354 | 4.089746 | 0.912068 | 0.530612 | 389 | 2.71978 | 0.874045 | 0.58 |
| 355 | 1.502074 | 0.559076 | 0.322581 | 390 | 2.668829 | 0.812958 | 0.529915 |
| 356 | 2.986151 | 0.814567 | 0.482759 | 391 | 2.059944 | 0.649206 | 0.540541 |
| 357 | 2.684544 | 0.831897 | 0.510638 | 392 | 2.28943 | 0.608457 | 0.384615 |
| 358 | 2.940043 | 0.759483 | 0.5 | 393 | 1.51325 | 0.527817 | 0.360656 |
| 359 | 4.343338 | 0.879104 | 0.431035 | 394 | 3.26734 | 0.823425 | 0.423729 |
| 360 | 2.736949 | 0.524466 | 0.5625 | 395 | 2.372039 | 0.751457 | 0.648352 |
| 361 | 2.709884 | 0.76271 | 0.438597 | 396 | 1.786959 | 0.528899 | 0.5625 |
| 362 | 2.665006 | 0.816458 | 0.6 | 397 | 2.546283 | 0.869478 | 0.476191 |
| 363 | 2.98424 | 0.874802 | 0.505747 | 398 | 2.542531 | 0.807804 | 0.516667 |
| 364 | 2.538358 | 0.70338 | 0.42 | 399 | 1.81252 | 0.801958 | 0.309524 |
| 365 | 1.894349 | 0.63375 | 0.461539 | 400 | 1.6924 | 0.740197 | 0.548387 |
| 366 | 3.417806 | 0.83194 | 0.478261 | **mean** | **2.89** | **.806** | **.506** |
| 367 | 3.47404 | 0.932212 | 0.433962 |  |  |  |  |
| 368 | 2.755261 | 0.84061 | 0.495798 |  |  |  |  |
| 369 | 1.818957 | 0.680784 | 0.469388 |  |  |  |  |
| 470 | 1.550422 | 0.786094 | 0.470588 |  |  |  |  |
| 371 | 2.009765 | 0.804998 | 0.405063 |  |  |  |  |
| 372 | 2.774194 | 0.611781 | 0.333333 |  |  |  |  |
| 373 | 5.556362 | 0.835821 | 0.396552 |  |  |  |  |
| 374 | 1.904607 | 0.820284 | 0.419355 |  |  |  |  |
| 375 | 1.988851 | 0.939454 | 0.43617 |  |  |  |  |
| 376 | 2.58512 | 0.727618 | 0.3 |  |  |  |  |
| 377 | 1.995091 | 0.776757 | 0.354167 |  |  |  |  |
| 378 | 2.985717 | 0.736786 | 0.428571 |  |  |  |  |
| 379 | 1.806911 | 0.359451 | 0.36 |  |  |  |  |
| 380 | 1.230048 | 0.524026 | 0.333333 |  |  |  |  |
| 381 | 1.53587 | 0.328553 | 0 |  |  |  |  |
| 382 | 1.645692 | 0.651497 | 0.264706 |  |  |  |  |
| 383 | 2.768195 | 0.74499 | 0.444444 |  |  |  |  |
| 384 | 2.253623 | 0.804214 | 0.461539 |  |  |  |  |
| 385 | 1.756511 | 0.721271 | 0.533333 |  |  |  |  |
