# Supplementary material for: Impacts of agricultural machine renting on cereal crop productivity and commercialization in West Gojjam Zone, Ethiopia
Source: PLoS One. 2025 Oct 27;20(10):e0300831. doi: 10.1371/journal.pone.0300831 (PMC12558508; doi:10.1371/journal.pone.0300831)
Supplement: Attachment — Submitted filename: PONE-D-24-08983.pdf [file pone.0300831.s003.pdf]

PLOS ONE

Impacts of Agricultural Machine renting on Cereal Crop Productivity and Commercialization in West Gojjam Zone, Ethiopia.

--Manuscript Draft--

**Manuscript Number:**	PONE-D-24-08983
**Article Type:**	Research Article
**Full Title:**	Impacts of Agricultural Machine renting on Cereal Crop Productivity and Commercialization in West Gojjam Zone, Ethiopia.
**Short Title:**	Impacts of Agricultural Machine renting on Cereal Crop
**Corresponding Author:**	Selam Tilahun<br>Wolaita Sodo University<br>Sodo, ETHIOPIA
**Keywords:**	Agricultural machine, Productivity, Commercialization, Endogenous switch regression, Impact
**Abstract:**	This study investigated the impacts of agricultural machine renting on cereal crop productivity and commercialization. The study used cross-sectional data collected from 192 agricultural machine users and 208 non-users from West Gojjam zone, Northern Ethiopia. The data were analyzed using descriptive statistics and econometric models. The econometric model includes Trans log production function, Endogenous switch regression and propensity score matching. The result of descriptive statistics showed that the total factor productivity index of the cereal crop was 2.89, and the mean commercialization index for the sample households was .0.506.The estimation of results of Trans log production function model showed an average level of Technical Efficiency (TE) of 80.6%. the result of first stage Endogenous switch regression model showed that age, education level, ownership of oxen, total cultivated land, extension visit, access to information, family size, the position a farmer, and distance to the nearest market significantly affected adoption of agricultural machine renting (tractor, combine harvester and maize Sheller).The result of endogenous switching regression and propensity score matching models showed that agricultural machine renting had a positive and significant impact on the selected outcome variables, total factor productivity, technical efficiency and output commercialization index. Based on the findings, the study suggests that the government and stakeholders should focus on strengthening the provision of formal and informal education, development of infrastructures, extension service, to promote agricultural machine renting service, and enhance cereal crops productivity and commercialization.
**Order of Authors:**	Selam Tilahun
	Berhanu Kuma
	Amsalu Bedemo
**Additional Information:**	
**Question**	**Response**
**Financial Disclosure**<br><br>Enter a financial disclosure statement that describes the sources of funding for the work included in this submission. Review the submission guidelines for detailed requirements. View published research articles from *PLOS ONE* for specific examples.	The author(s) received no specific funding for this work.

Powered by Editorial Manager® and ProduXion Manager® from Aries Systems Corporation

This statement is required for submission and **will appear in the published article** if the submission is accepted. Please make sure it is accurate.<br><br>**Funded studies**<br>Enter a statement with the following details:<br>• Initials of the authors who received each award<br>• Grant numbers awarded to each author<br>• The full name of each funder<br>• URL of each funder website<br>• Did the sponsors or funders play any role in the study design, data collection and analysis, decision to publish, or preparation of the manuscript?<br><br>Did you receive funding for this work?	
**Competing Interests**<br><br>Use the instructions below to enter a competing interest statement for this submission. On behalf of all authors, disclose any competing interests that could be perceived to bias this work—acknowledging all financial support and any other relevant financial or non-financial competing interests.<br><br>This statement is required for submission and **will appear in the published article** if the submission is accepted. Please make sure it is accurate and that any funding sources listed in your Funding Information later in the submission form are also declared in your Financial Disclosure statement.<br><br>View published research articles from *PLOS ONE* for specific examples.	The authors declare no conflict of interest.

Powered by Editorial Manager® and ProduXion Manager® from Aries Systems Corporation

**NO authors have competing interests**<br><br>Enter: *The authors have declared that no competing interests exist.*<br><br>**Authors with competing interests**<br><br>Enter competing interest details beginning with this statement:<br><br>*I have read the journal's policy and the authors of this manuscript have the following competing interests: [insert competing interests here]*<br><br>* typeset	
**Ethics Statement**<br><br>Enter an ethics statement for this submission. This statement is required if the study involved:<br><br>• Human participants<br>• Human specimens or tissue<br>• Vertebrate animals or cephalopods<br>• Vertebrate embryos or tissues<br>• Field research<br><br>Write "N/A" if the submission does not require an ethics statement.<br><br>General guidance is provided below. Consult the submission guidelines for detailed instructions. **Make sure that all information entered here is included in the Methods section of the manuscript.**	No permit number.it is PhD dissertation

Powered by Editorial Manager® and ProduXion Manager® from Aries Systems Corporation

**Format for specific study types**<br><br>**Human Subject Research (involving human participants and/or tissue)**<br>• Give the name of the institutional review board or ethics committee that approved the study<br>• Include the approval number and/or a statement indicating approval of this research<br>• Indicate the form of consent obtained (written/oral) or the reason that consent was not obtained (e.g. the data were analyzed anonymously)<br><br>**Animal Research (involving vertebrate animals, embryos or tissues)**<br>• Provide the name of the Institutional Animal Care and Use Committee (IACUC) or other relevant ethics board that reviewed the study protocol, and indicate whether they approved this research or granted a formal waiver of ethical approval<br>• Include an approval number if one was obtained<br>• If the study involved *non-human primates*, add *additional details* about animal welfare and steps taken to ameliorate suffering<br>• If anesthesia, euthanasia, or any kind of animal sacrifice is part of the study, include briefly which substances and/or methods were applied<br><br>**Field Research**<br><br>Include the following details if this study involves the collection of plant, animal, or other materials from a natural setting:<br>• Field permit number<br>• Name of the institution or relevant body that granted permission	
**Data Availability**<br><br>Authors are required to make all data underlying the findings described fully available, without restriction, and from the time of publication. PLOS allows rare exceptions to address legal and ethical concerns. See the PLOS Data Policy and FAQ for detailed information.	Yes - all data are fully available without restriction

A Data Availability Statement describing where the data can be found is required at submission. Your answers to this question constitute the Data Availability Statement and **will be published in the article**, if accepted.<br><br>**Important:** Stating 'data available on request from the author' is not sufficient. If your data are only available upon request, select 'No' for the first question and explain your exceptional situation in the text box.<br><br>Do the authors confirm that all data underlying the findings described in their manuscript are fully available without restriction?	
**Describe where the data may be found in full sentences. If you are copying our sample text, replace any instances of XXX with the appropriate details.**<br><br>• If the data are **held or will be held in a public repository**, include URLs, accession numbers or DOIs. If this information will only be available after acceptance, indicate this by ticking the box below. For example: *All XXX files are available from the XXX database (accession number(s) XXX, XXX.*).<br>• If the data are all contained **within the manuscript and/or Supporting Information files**, enter the following: *All relevant data are within the manuscript and its Supporting Information files.*<br>• If neither of these applies but you are able to provide **details of access elsewhere**, with or without limitations, please do so. For example:<br><br>*Data cannot be shared publicly because of [XXX]. Data are available from the XXX Institutional Data Access / Ethics Committee (contact via XXX) for researchers who meet the criteria for access to confidential data.*<br><br>*The data underlying the results presented in the study are available from (include the name of the third party*	XXX

and contact information or URL).<br>• This text is appropriate if the data are owned by a third party and authors do not have permission to share the data.<br><br>* typeset	
Additional data availability information:	

Powered by Editorial Manager® and ProduXion Manager® from Aries Systems Corporation

Manuscript Click here to access/download;Manuscript;Ploss.docx

Impacts of Agricultural Machine renting on Cereal Crop Productivity and Commercialization in West Gojjam Zone, Ethiopia.

SelamTilahun[a] ,BerhanuKuma[b] and AmsaluBedemo[c]

a. **Email:selam4998@gmail.com,**Department of Agricultural Economics, Wolaita Sodo University
b. **Email:berhanukuma@yahoo.com,** Department of Agricultural Economics, Wolaita Sodo, University
c. **Email:amsalu72@gmail.com,** Department of Economics, Addis Ababa, University

ABSTRACT

This study investigated the impacts of agricultural machine renting on cereal crop productivity and commercialization. The study used cross-sectional data collected from 192 agricultural machine users and 208 non-users from West Gojjam zone, Northern Ethiopia. ~~The data were analyzed using descriptive statistics and econometric models.~~ The econometric model includes Trans log production function, Endogenous switch regression and propensity score matching. The result of descriptive statistics showed that the total factor productivity index of the cereal crop was 2.89, and the mean commercialization index for the sample households was .0.506.The estimation of results of Trans log production function model showed an average level of Technical Efficiency of 80.6%. the result of first stage Endogenous switch regression model showed that age, education level, ownership of oxen, total cultivated land, extension visit, access to information, family size, the position a farmer, and distance to the nearest market significantly affected adoption of agricultural machine renting (tractor, combine harvester and maize Sheller).The result of endogenous switching regression and propensity score matching models showed that agricultural machine renting had a positive and significant impact on the selected outcome variables, total factor productivity, technical efficiency and output commercialization index. Based on the findings, the study suggests that the government and stakeholders should focus on strengthening the provision of formal and informal education, development of infrastructures, extension service, to promote agricultural machine renting service, and enhance cereal crops productivity and commercialization.

Keywords: Agricultural machine, Productivity, Commercialization, Endogenous switch regression, Impact

1. INTRODUCTION

Sub-Saharan Africa (SSA) is one of the regions in the world that is mainly characterized by smallholder farm households, ~~where their~~ livelihood depends primarily on agriculture. Similarly, Ethiopia's economy mainly depends on agriculture, which is the main source of income for 84% of the population, and it ~~accounts about~~ 33.8% of the country's ~~Gross Domestic Product~~ (GDP) and 70% of export earnings [1]. Cereal cultivation and marketing ~~is~~ the primary ~~source~~ of income for millions of subsistence farmers in the country. Cereals ~~are accounts~~ for 60 ~~percent~~ of rural employment in Ethiopia, 80% of cultivated land in the country, over 40% of food expenditure for the typical household, and over 60% of total calorie intake [2]. Cereals are the main food crop in terms of area coverage and production.

Cereal crop production and marketing have shown remarkable progress in Ethiopia, yet the domestic price for cereals is growing from time to time due to the rapid increase in domestic demand, rapid population growth, income, and urbanization [3]. Given the importance of cereal crops in national food security endeavors, smallholder farmers have used traditional methods of production with limited mechanization. ~~Thus low~~ productivity and, limited adoption of agricultural ~~technologies including~~ ~~mechanizations and~~ subsistence-based smallholder farming are characteristics of the sector [4,5]. Specifically, a low level of mechanization in agriculture continues to be the main obstacle to advancing cereal production, which, in turn, results in a high cost of production [6]. As a result, agricultural mechanization is among the mechanisms that increase production and productivity and reduce food insecurity [7].In the same way, according to [8] and [9] the utilization of appropriate agricultural mechanization technologies helps to increase the productivity and commercialization of cereal crops.

.

However, there ~~is a~~ public debates about the ~~benefit~~ of agricultural mechanization. According to the International Labor Organization (ILO, 1973) "mechanization leads to unemployment and is linked to economies of scale, meaning that it is more likely to be bought by larger farms [10,11]. In ~~Africafew~~ studies ~~have conducted~~ on impacts of mechanization on employment, and those that have mostly reported mixed effects [12,13,14,15] and positive ~~effect~~ [16,17]. According to

[18] the impacts of mechanization on employment depend on location-specific factors such as wages, land availability, and type of mechanization. On the other hand, smallholder farmers can take advantage of mechanization by renting markets and cooperative exchange [19,20,21].

Ethiopia's agriculture sector has depended on inexpensive and surplus labour for a long time. However, the condition is currently changing ~~owning to~~ the rapid migration of young people from rural to urban areas and the availability of universal access to education for all school-aged children [22,23]. This has led to a shortage of farm labor and pressure on the remaining household members. Additionally, the cost of oxen and lack of grazing land have ~~become a major concern~~ for smallholder farmers [19]. Consecquently, the use of machinery rental services has become a viable option for smallholder farmers. Recently, renting agricultural machines by smallholder farmers has been used in Ethiopia. Likewise, the Amhara region is one of the cereal crop growing areas and agriculture is the primary economic sector, but only 4.35% of agricultural activities oprate mechanically.

A number of studies have been conducted on the factors affecting the adoption of agricultural technology in Ethiopia; for example[24,25,26,27,28,29]. Only a few studies have been conducted on what may be the determinant of the adoption of the agricultural mechanization services with no specific studies on the agricultural machine renting services [30,31,32]. These previous studies applied logit, linear probability and tobit models to examine factors affecting agricultural mechanization. An important innovation in this paper was examining the effects of agricultural machine renting in Ethiopia on the productivity and commercialization of cereal crops. Hence there is no study on the impact of agricultural mechanization on productivity and commercialization which enables us ~~to identify its impact, and helps to design an appropriate policy towards fostering the adoption of agricultural machines.~~

Before the methodology, it should be needed literature review both theoretical and empirical review, and the authors should clarify how the conceptual framework guides the study.

2. METHODOLOGY

2.1. Area Description

The area of description part is shallow and it needs more explanation in terms agricultural machine renting on cereal crop productivity and commercialization in West Gojjam Zone particularly your selected districts.

The study was carried out in the West Gojjam Zone of the Amhara region. West Gojjam zone, is one of the eleven (11) administrative zones established under the Amhara region. The administrative zone is bordered to the north by the Northern and Southern Gonder zones; to the east by East Gojjam zone; to the south by Oromia region and to the west by Benshangul Gumuz region. Figure 1 shows the study area of the research.

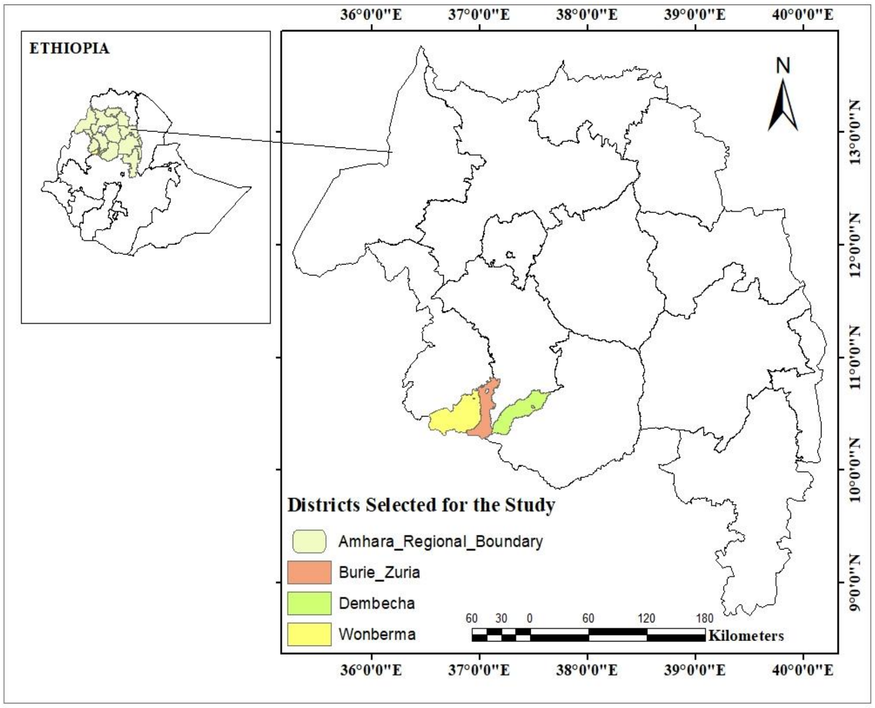

Figure 1: Map of the study area

Source:http://data.humdata.org/

2.2. Sampling Procedure and Sample Size Determination

To determine the sample size, you should not show the sampling techniques procedures.

A multi-step sampling procedure was used for the selection of the districts, the kebeles and the sample households. ~~The first stage included the selection of three districts (wonberma, buriezuria and dembecha) from the areas where agricultural machine rental practices took place. The~~

Which random sampling techniques were employed to select the sample respondents?

second stage involved the selection of two randomly selected kebeles for each district. The households were divided into two groups: those who adopted agricultural machines and those who did not. The third stage involved randomly selecting 400 household heads. The 400 household head sample consisted of 121 households (wonberma), 123 households (buriezuria) and 156 households (dembecha district) from each strata. Proportional sampling was used to determine the number of households in each strata, taking into account the size of the district and the kebel. ~~The final sample consisted of 192 households that had adopted at least one agricultural machine, 208 households that had not adopted an agricultural machine, were randomly selected from both strata using proportionate probability sampli~~ng based on the size of each district and kebel in the 2020/21 production season as shown Table 1

Cochran’s ~~(1963)~~ method was used determine the sample size. Cochran's method is particularly suitable for stratified sampling designs, where the population is divided into distinct subgroups or strata, and is beneficial when the population size is large or unknown. The equation is written as:

$$n = \frac{z^2\, pq}{d^2}\,(eq)(1)$$

where n is a minimum sample size, Z is 1.96 at 95 % confidence level; p is smallholder farmers who may adopt in agricultural machine (50 %); q is the weight variable and is computed as 1-p), and d is the desired precision or margin of error, expressed as a fraction of 0.05.

How did you calculate the sample households? it is not clear; thus, please show the calculation.

Table 1.Selected districts, kebeles and their sample sizes

District	Total hhs	Sampled Kebeles	Total hhs	Sample hhs	
Wenberma	18644	Markuma	1029	58	121
		Koki	1124	63	
Buriezuria	18929	Alefa	989	68	123
		Wadra	808	55	
Dembecha	23958	Wade	659	53	156
		Asteboch	1300	103	
Total	61756		7332	400	

The sample HHs are 69, 76, 67, 55, 45 and 88, repectively.

61,531

the summation of total hhs in sampled kebels are 5909.

2.3. Data Types and Sources

Both quantitative and qualitative data were used in this study. both secondary and primary data sources were used to generate data types. Primary data sources are farmers who rent agricultural machines and non-users of these machines. Secondary data sources include journals, books, CSA and internet browsing, national policies, and zonal, district and kebele reports.

I have not seen photos in the discussion part. What is the important for setting for data collection method part?

2.4. Data Collection Methods

The major data collection methods included formal surveys, ~~observations,~~ visual aids, discussions with key informants and focus groups.The primary data were collected though face-to-face personal interviews using a semi-structured questionnaire. The data were collected based on the research objectives. The questionnaire included both open and closed-ended questions to collect information relevant to the scope of the study. The questionnaire was first tested prior to the field survey for suitability (clarity, suitability and sequence of questions) and then re-tested based on the pre-test feedback.. In addition to interviews, discussion with each of the six selected kebeles extension agents who are working related to agricultural mechanization service (mechanization experts) were disscussed.

2.5. Methods of Data Analyses

Two types of data analysis methods were used to addressthe rereasch o~~bjective~~: ~~descriptive statistics~~ and econometric models.

Under the econometric model, you should be separately explained the theoretical and analytical framework, and then you should be specify the model and the conditional expectations, treatment, and heterogeneity effect.

Measurement of cereal crop productivity

In this study, TFP is the ratio of output to the total variable cost (TVC). This approach Total Fixed Cost (TFC), does not role since it is constant and does not have any effects ~~the~~ on the profit maximization and resource-use efficiency conditions [33]. According to [34] and [35] TFP for cross-sectional data or data within a specified time can be measured as the inverse of the unit variable cost because TFP is the ratio of the output and the Total Variable Cost (TVC) as shown in this equation;

$$TFP = \frac{Y}{TVC} = \frac{\sum_{i=1}^{n} Y}{\sum_{j=1}^{n} PijXji} \qquad eq(2)$$

$$AVC = \frac{TVC}{Y}$$

$$TFP = \frac{Y}{TVC} = \frac{1}{AVC}$$

Where, Yi : The output of cereal crops (in value or in quantities);

TVCi: Total Variable Cost of farm i;

Pji: Unit price of jth variable input used in farm i;

Xji: Quantity of jth variable input used in farm i.

Measurement of market participation

It is so shallow; it needs more explanation with supporting theoretical and empirical literature review.

Measuring Crop Output Market Participation Index (COMPI): The output side of commercialization (market participation) can be seen at the household or community level. At the household level, one definition of agricultural commercialization is defined as the ratio of the value of agricultural outputs sold to the total value of agricultural outputs produced by a household [36,37,38]. Hence, output side commercialization index or market participation for a household (COMPI) can be computed as:

$$COMPL_K = \sum_{i=1}^{N} \frac{S_{ki}}{Q_{ki}} \quad ; \quad Q_{ki} \geq S_{ki} \qquad \text{eq (3)}$$

Where, $COMPI_k$ is the proportion of crop k sold (S_{ki}) to the total amount produced (Q_{ki}) aggregated over the total sample households in a given location.

Efficiency and productivity analysis

The study used the production data of cereal crops (teff, wheat and maize) in the 2020/21 production season. The stochastic frontier methodology was later developed by[39,40]. It is specified as

In addition to these variables, why not incorporated agricultural machines (like tractor, combiner, and Sheller) as inputs for the production of cereal crops?

$$y_i = f(x_{i,}\beta) + \varepsilon_i(v_i - u_i) \qquad eq(4)$$

Where: Yi is the amount of the cereal crops yield produced by ith household in the 2020/21 production year that is expressed in quintals; is a production function $f(x_{i,}\beta)$are the parameters to be estimated; X's are exogenous input factors (labour, land, fertilizer seed and herbicide) affecting the amount of yield; $\varepsilon i$is the error term, equal to ($Vi - Ui$); Vi is a two-sided random error component beyond the control of the farmer, and $Ui$is one-sided inefficiency component.

Given the level of input, the TE of the ith cereal crop producer farmers are the ratio of the observed output to the maximum potential output (frontier output):

$$TE_i = \frac{actual\ output}{potential\ output} = \frac{f(x_i;\beta)\exp(v_i - \mu_i)}{f(x_i;\beta)\exp(v_i)} = \exp(-\mu_i)$$

The specification of the endogenous switching regression model should not well organize. So, please specify ESR model with supporting the theoretical and empirical literature review.

2.6. The impacts of agricultural machines renting on productivity and commercialization

To measure the impact of agricultural mechanization on the productivity and commercialization of cereal crops, endogenous switch regression (ESR) and propensity score matching (PSM) models were employed.

When assessing the impact of adopting an agricultural machine on the productivity commercialization of a farm household, it is important to capture both the observable and unobserved characteristics of the adopter (treatment group) as well as the non-adopter (control group). However, the majority of impact assessment methods that use non-experimental data do not capture the observable and/or non-observable characteristics that influence adoption as well as outcome variables. For example, instrumental variables only capture unobserved heterogeneity but assume that the parallel shift in outcome variables can be seen as a treatment effect [41,42,43].

The decision to adopt a technology can be modeled in a random utility framework by expressing the unobservable utility from adoption and non-adoption through observable variables[44].We specify the selection equation for technology adoption as:

$$\boldsymbol{P_i^*} = Zi\beta + U_i \qquad P = \begin{cases} 1, & if\ p *> 1 \\ 0, & otherwise \end{cases} \qquad eq(5)$$

Where$\boldsymbol{P_i^*}$: is unobservable or latent variable for machine renting, ~~Yi~~ is its observable counterpart (the dependent variable use of agricultural machines equals one, if the farmer has adopted at least one agricultural machine during 2020/21 cropping season, and zero otherwise), Zi are non-stochastic vectors of observed farm and non-farm characteristics determining adoption and Ui is random disturbances associated with renting of agricultural machine of improved technology.

The two regimes that the smallholder farmers fall in to are represented by the following two regression equations.

$$Regime\ 1: Y_I = \alpha_1 X_i + e_{1i} \qquad if\ p = 1 \qquad eq(6)$$

$$Regime\ 2: Y_2 = \alpha_2 X_i + e_{2i} \qquad if\ p = 0 \qquad eq(7)$$

Y1i and Y2i are the dependent outcome variables determined by the exogenous variables Xi, α1, and α2, are parameters that show the direction and strength of the relation between the outcome variable and the independent variables. e_{1i} ande_{2i} are error terms.

Finally, the error terms are assumed to have a trivariate normal distribution, with zero mean and non-singular covariance matrix expressed as:

$$cov(u_i, e_{1i}, e_{2i}) = \begin{bmatrix} \sigma^2 u & . & . \\ \sigma^2 e_1 u & \sigma^2 e_1 & . \\ \sigma^2 e_2 u & . & \sigma^2 e_2 \end{bmatrix} \qquad eq(8)$$

Where $\sigma^2 u$ variance of the error term in the selection equation, $\sigma^2 e_1$ and $\sigma^2 e_2$ are variances of the error terms in the continuous equations. $\sigma^2 e_1 u$and$\sigma^2 e_2 u$ are covariance of $u_i, and\ \ e_{1i}\ and\ e_{2i}$respectively. Since Y_{1i} and Y_{21} are not observed simultaneously a covariance of the corresponding error terms is not defined [45]. This structure of the error terms indicates

that the error terms of the outcome equation and the error term of the selection equation are correlated which results in non-zero expected value of e_{1i} and e_{2i} given ui - error term of the selection equation [46]. Therefore, the expected values of the truncated error terms E(e_1 | p = 1) and $E(e_2$ | p = 0) are given below:

$$E\,(e_1 \mid p = 1) = E(e_1 \mid U > - Z\beta)$$

$$+= \sigma e_1 u \frac{\varphi(\frac{Z\beta}{\sigma})}{\Phi \frac{Z\beta}{\sigma}} = \sigma e_1 u \lambda_1 \qquad eq(9)$$

$$E\,(e_2 \mid p = 1) = E(e_2 \mid U \leq - Z\beta)$$

$$= \sigma e_1 u \frac{-\varphi(\frac{Z\beta}{\sigma})}{1 - \Phi(\frac{Z\beta}{\sigma})} = \sigma e_2 u \lambda_2 \qquad eq(10)$$

φ and Φ are the probability density and cumulative distribution function of the standard normal distribution, respectively. The ratio of φ and Φ evaluated at Zβ is referred to as the inverse Mills ratio $\lambda 1$ and $\lambda 2$ (selectivity terms). If the estimated covariance $\sigma^2 \varepsilon 1u$ and $\sigma^2 \varepsilon 2u$ are significantly different from 0 the decision to renting agricultural machine and the outcome variable (productivity and commercialization) are correlated. This implies endogenous switching and the presence of a sample selectivity bias [47]. Further, estimations of treatment effects will be made. Average Treatment effect on the Treated and Untreated (ATT and ATU) are computed using the results for expected values of the dependent variable for users and non-users in actual and counterfactual scenarios:

$$E(Y_{1i}|p_i = 1, X_{1i}) = \alpha_1 X_{1i} + \sigma e_1 u \rho_1 \frac{\varphi(z\beta)}{\Phi(z\beta)} \qquad eq(11)$$

$$E(Y_{2i}|p_i = 0, X_{2i}) = \alpha_2 X_{2i} - \sigma e_2 u \rho_2 \frac{\varphi(z\beta)}{(1 - \Phi(z\beta)} \qquad eq(12)$$

$$E(Y_{2i}|p_i = 1, X_{1i}) = \alpha_2 X_{1i} + \sigma e_2 u \rho_2 \frac{\varphi(z\beta)}{\Phi(z\beta)} \qquad eq(13)$$

$$E(Y_{1i}|p_i = 0, X_{2i}) = \alpha_2 X_{2i} - \sigma e_1 u\rho_2 \frac{\varphi z\beta}{(1 - \Phi(z\beta)} \qquad eq(14)$$

ATT is the difference between the expected value of the outcome variable from Equations 11 and 13; this is the difference between the expected value of the dependent variable for users and if they were not used. ATU is the difference between Equations 12 and 14 which estimates the difference between the expected value of the outcome variable for non-users of agricultural machine.

PSM techniques were applied to supplement the ESR model and to assess the consistency of the results with different assumptions such as nearest neighbour matching, caliper and radius matching, and kernel and local linear matching [48]. The treatment effects will be estimated based on matching estimators selected on the common support region [49]. The average treatment effects can be estimated using the inverse propensity weighing estimates as stated in IPSW [50] using matching techniques of Kernel Matching (KM), Nearest Neighbor Matching (NNM) and Radius Caliper Matching (RCM).

The selection of a particular approach depends on the data in question and, in particular, the degree of similarity between the treatment and control groups in terms of propensity score [51].The Average Treatment Effect of an individual i can be written as:

$$\mathrm{ATE} = \mathrm{E}(\mathrm{Y}_\mathrm{i}^1|\mathrm{D}_\mathrm{i} = 1) - \mathrm{E}\left(\mathrm{Y}_\mathrm{i}^0 \,\middle|\, \mathrm{D}_\mathrm{i} = 0\right) \qquad \mathrm{eq}(15)$$

Where ATE, Average Treatment Effect, which is the effect of treatment on the outcome variables:

$E(Yi^1 | Di=1)$: Average outcomes for individual, with user, if he/she would participant
$E (Yi^0 | Di=0)$: Average outcome of non-user, when he/she would non-participant

The dependent variable:

Adoption of agricultural machine renting: A dichotomous dependent variable measured in participation of agricultural machine renting, taking value 1 if there is participation in agricultural machine renting and 0 otherwise.

Outcome variable

Total factor productivity: Total factor productivity index of cereal crops includes (teff, wheat and maize) produced by households in the 2020/21 production year.

Technical efficiency: Technical efficiency of cereal crops (teff, wheat and maize) in the 2020/21 production season.

Output commercialization: Cereal crop output commercialization indices include teff, wheat and maize output commercialization indicesin the 2020/21 production season.

The independent variables: the explanatory variables that were assumed to affect both the participation of households in renting machines and the outcome variable are age, family size, level of education, total cultivated land credit use, frequency of extension contact, participation in off-farm activity, distance to the nearest market, distance from road, distance from DA office, position of the farmer in farmer's association, ownership oxen, livestock and membership of cooperative.

It is not only listing the independent variable; you should be defined each independent variables and hypothesize based on the literature review.

3. RESULTS AND DISCUSSION

3.1. Demographic, Socio-Economic and Institutional Characteristics

Table 2 shows the statistical reports of the demographic, socio economic and institutional characteristics of the sampled households. The age of the sample households ranged from 24 to 70 years, with an average age of 44.718 years. Moreover, the mean typical household attended approximately 4 years of formal education. The selected households owned on average four livestock in TLU, with a minimum of none and a maximum of twelve, excluding the ownership of oxen. The mean total land holding of households was about 2.14ha with a minimum of 0.25 and a maximum of 5 hectares. In addition, the mean own land holding of the sampled household was 1.45 hectares. Households in the study area have an average of six members with a mean total dependency ratio of 0.49.

Table2.Demographic, socio-economic and institutional characteristics for continuous variable

Variable	Obs	Mean	Std. Dev.	Min	Max
Age of hh	400	44.75	9.64	24	70
Education level	400	3.78	3.08	0	12
Farming experience	400	20.05	9.68	1	48
Family size	400	6.12	1.69	1	12
distance to market	400	3.19	2.39	0.1	16
Distance to main road	400	2.37	1.54	0.1	7
Distance to DA	400	2.29	1.36	.15	8
Extension visit	400	11.03	6.21	1	30
Total land	400	2.14	0.92	0.25	5
Ownership of oxen	400	2.39	0.97	0	6
TLU	400	4.00	2.06	0	12
Own land	400	1.44	0.77	0	4

Source: Computed from survey data (2021)

The sex of the sample of farm household heads, about 94.75% were male headed. The result indicated that about 43.25% of the households used credit services for different purposes. As shown in Table 3 below, about 79.25% of the sampled household heads were members of cooperatives, and about 49.25% of the sampled respondents also heldpositions in social activities (edir ,equib and position on agricultural activities).

Table 3. Demographic, socio-economic and institutional characteristics for categorical variable

Variable	Description	No of households(n=400)	Percent
Sex of household head	Male	379	94.75
	Female	21	5.25
Position social participation	Yes	195	48.75
Credit use	Yes	173	43.25
Membership of cooperative	Yes	317	79.25
Off -farm participation	Yes	131	32.75
Machine renting	Yes	192	48
access information EA	Yes	282	70.5

Source: Computed from survey data (2021)

3.2 Agricultural machine renting

In the study area, tractors, combine harvestesrs and maize Sheller are used in the production process of cereal crops, either solely or simultaneously. The study showed that Table 4 approximatly 25.3% rented tractors only for ploughing services. Farmers commonly rent tractors in the study area for the first and second tillage. This is becauset the first ploughing operation is the heaviest and most challenging for oxen, whereas a tractor can plough easily and deeply. Similarly, about 22.3% of farmers rented combined harvesters only for wheat harvesting, threshing and winnowing operation and about 28.54% rented maize Sheller machines only for shelling of maize after harvesting. About 11.16% of farm households rent three types of machines (tractor, combine harvester and maize Sheller) simultaneously.

Table 4.Major agricultural machine used in the study area

Types of agricultural machines used	Freq.	Percent
Tractor	118	25.3
Combiner	104	22.3
Sheller	133	28.54
All (tractor, combiner and Sheller)	52	11.16
Tractor &combiner	17	3.65
Tractor & Sheller	20	4.3
Combiner & Sheller	22	4.72

The investigation needs deeper and detail discussion for the implications of the findings and relate them to the existing literature.

3.3. Impacts of agricultural machine renting on productivity and commercialization

Table 5 represents the first stage ESR binary probit estimation results. The probit model fits the data reasonably well [Wald Chi-squared = 137.31, p= 0.000)]. The model results reaveled that household, socioeconomic, and institutional factors had a significant impact on the decision to rent agricultural machines.

The age of the household head affected adoption of agricultural machine renting positively and significantly at 1% level. The marginal effect result indicates that aged household heads adopt more agricultural machines than younger households because, as the age of households increases, working power decreases. Agricultural machine renting increased with the level of education of the household heads. Household heads who attened better education level have a good awareness about agricultural machines. The total land cultivated significantly and positively affected the adoption of agricultural machines. Farmers who owned large amount of cultivated land were more likely to adopt agricultural machines than small land-holding farmers. Ownership of large amounts of cultivated land often required more labor and time to perform agricultural operations.

Frequency of extension contact influenced the adoption of agricultural machines significantly and positively at the 1% level. This implies that farmers who are frequently in contact with extension agents are more likely to adopt agricultural machines than are those in lower contact with extension agents. This is because, development agents provide advice on agricultural machine utilization and benefits to farmers. The extension provides in-depth information, training and consultancy services on the source, application and relevance of agricultural technologies for farmers [52]. Similarly, farmers with information about agricultural machines from government extension agents are more likely to adopt agricultural machines. This is because the provision of information helps farmers to become aware of the benefits and costs of machines. The use of credit negatively and significantly affected the probability of farmers using agricultural machines to produce cereal crops at 1%. The result indicated that farmers who used

This citation appearing in the narrative was not shown in the reference section.

credit services decreased the probability of agricultural machines renting. This may be because households used credit for consumption, fattening livestock and home construction.

Distance from the market and the main road negatively and significantly affected the adoption of agricultural machines by 10% and 5%, a significant level. Proximity to the market and main roads are important factor in adopting agricultural machines because better access to markets and roads is an indicator of access to information and infrastructure. Thus, farmers who live near the market and main road are more likely to rent agricultural machines and other important inputs at a lower cost than those who live far away from markets and main roads. The number of oxen affects the adoption of agricultural machines positively and significantly at 1%. This implies an increase in the number of oxen and the probability of adopting agricultural machines increases. This means that households with many oxen perform agricultural activities like the second plow, third plow, sowing and threshing more easily than thos fewer oxen.

The results of the second stage ESR and,the estimated coefficients of total factor productivity, technical efficiency and commercialization index are presented in the appendix.

Table 5. Decision of adopting agricultural machines renting: Probit model

Variables	Coeff	Std.Err	Marginal Effect
Age of household	0.03***	0.009	0.012
Education level of household	0.09***	0.026	0.036
Adult equivalent	-0.11	0.059	-0.045
Poweron social activity	-0.49***	0.149	-0.196
Market distance	-0.07*	0.043	-0.031
~~Road distance~~	~~0.14**~~	~~0.073~~	~~0.058~~
Distance from office	0.1271	0.086	-0.050
Extension visit	0.0376***	0.012	0.015
Member of cooperative	0.2692	0.185	0.107
Credit use	-0.4276***	0.146	-0.170
Total land	0.3655***	0.040	0.145
TLU	-0.0372	0.041	-0.014
Number of oxen	0.3934***	0.110	0.156
Off farm activities	-0.0666	0.158	-0.026
Information for mechanization	0.3425**	0.165	0.136
-Cons-	-2.1487***	0.705	

Source: Computed from survey data (2021)

Table 6 shows the results ATT and ATU for the key outcome variables related to agricultural machine renting. In this analysis the main outcome vaiables are TFP, TE and COIM index. Before, we analyzed the result of ESR model to check the validity of instrumental variables using a falsification test. The falsification test shows the instrumental variable significantly affects the adoption of agricultural machines but not TFP, TE and COIM index. Instrument variables are jointly statistically significant in the adoption equation [$\chi 2 = 4.22$ ($p = 0.04$)] but not outcome functions. The instrumental variable did not affect the TFP [F=0.10(p= 0.7497)] technical efficiency [F=0.45 (p=0.5038)], and commercialization index [F= 1.92 (p=0.1668)].

The adoption of agricultural machines was renting increases total productivity, technical efficiency and commercialization level of farm households. For farmers who adopted agricultural machine renting but theoretically would have not adopted, then their TFP decreased by 0.573 (57.3%). This means, the ATT of total factor productivity of cereal crops would decrease by 57.3% as a result of did not use agricultural machines renting. Likewise, if non-users of agricultural machine adopted agricultural machine renting, then their TFP would have

significantly increased by 0.419(41.9%). For farmers who adopted agricultural machines but theoretically would not have adopted them, then their TE decreased by 0.104 (10.4). This means, the ATT of TE on cereal crops would decrease by 10.4% due to do not adopting agricultural machines. Similarly, if non-users of agricultural machine adopted agricultural machines, then their TE would have significantly increased by 0.064(6.4%). In addition, the results of the commercialization index of cereal crops for adopters would drop by 0. 12(12%) if the adopters were not adopted. In the same way, if the non-users of agricultural machine had adopted agricultural machine renting, their COIM would have increased by 0.098 (9.8%).

Consequently, ESR shows that in Ethiopia the adoption of agricultural machine renting would have increased productivity and commercialization of cereal crops. This result resembles to [53] the adoption of agricultural technology has a positive effect on crop productivity including wheat. The adoption of agricultural technologies has also a positive and significant impact on productivity of teff [54] The adoption of agricultural mechanization has a significant positive impact on the productivity of all types of crops [55].

Table. 6 Endogenous switching regression model result (average treatment effects)

Outcome variables	Mean farm household type and treatment effect	Decision stage		Treatment effect
		To Adopt	Not to Non-adopt	
Productivity	ATT	3.1	2.52	0.573***
	ATU	3.13	2.7	0.422***
TE	ATT	0.845	0.741	0.104***
	ATU	0.837	0.772	0.064***
COIM	ATT	0.572	0.452	0.120***
	ATU	0.544	0.444	0.099***

Note. ATT = Adoption effect for adopters, ATU = Adoption effect for non-adopters, and *** p <.01.

Popensity Score Matching Estimation Results

In addition to the ESR model, this study uses the PSM technique to check the robustness of the results obtained from the ESR model. Propensity scores (the probability of adoption in agricultural machine) are estimated using a probit model. Figure2 shows the distribution of adopter and non-adopter households with respect to estimated propensity scores. The figure illustrates the estimated propensity distribution for treatment and control households. The upper half of the graph refers to the propensity score distribution of treatment groups, while the bottom half shows the control groups. The y-axis refers to the densities of estimated propensity scores.

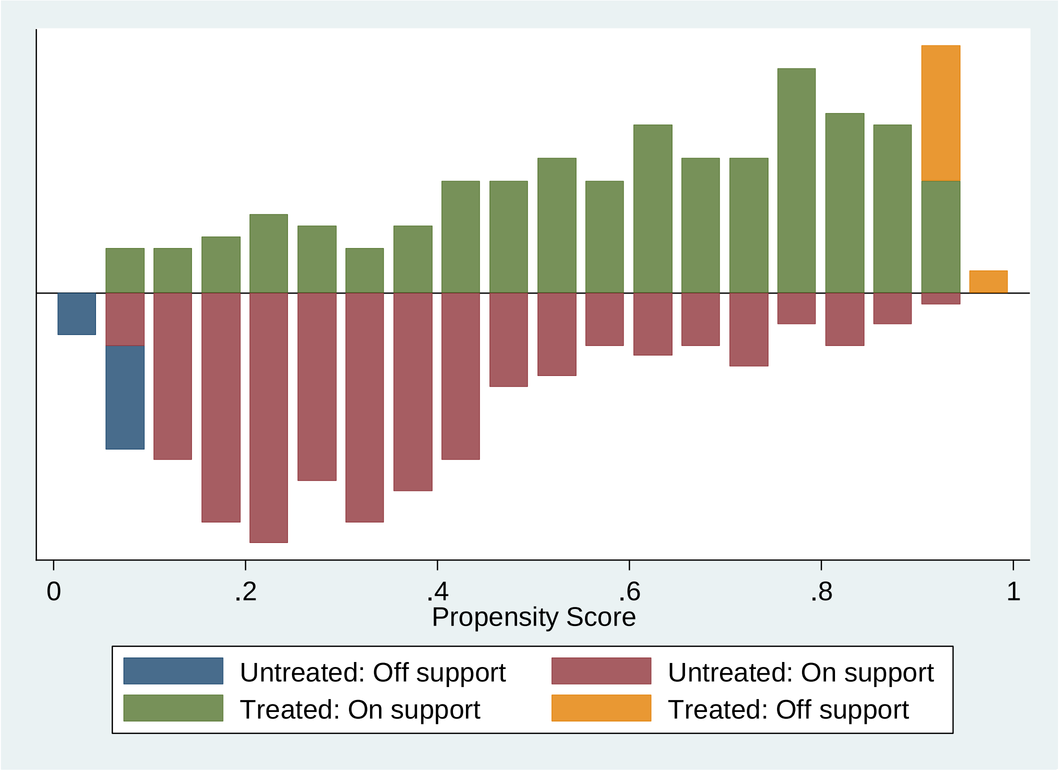

Figure 2: Distribution of estimated propensity distribution for treatment and control groups and common support area

There should be a common support condition that should be applied to how the propensity score is distributed between adopters of agricultural machine renting and those who do not. The

estimated propensity scores varied between 0.0826423 and 0.9633814 (mean =0. 6249002) and 0. 0347458 and 0.92732443 (mean, =0.3462459) in the treatment and control groups, respectively. The common support lies between 0.0826423 and 0.92732443. Table 7 presents the balancing tests for each matching algorithm before and after matching. The results show that the mean standardized bias is reduced after matching from 10.3 to 5.3 percent) compared to before matching (38percent). Similarly, Pseudo-R^2 declined substantially, from 22.4 percent to 1 percent. The p-values from the likelihood ratio tests showed that all of the covariates were statistically significant at a p-value of less than 1 percent prior to matching, but insignificant post-match. These p-values clearly demonstrate that the matching process balance the observed characteristics between treated and control after matching.

Table 7.Covariates balancing tests before and after matching

Matching algorism	Pesido-R-square		LRx^2P −value		Mean standard bias	
	Before	After	Before	After	Before	After
NNM_1	0.224	0.010	123.87 (0.00)	4.99 (0.986)	38	5.3
NNM_2	0.224	0.014	123.87 (0.00)	6.71 (0.278)	38.	6.9
KBM_3	0.224	0.010	123.87 (0.00)	4.83 (0.729)	38	6.6
KBM_4	0.224	0.011	123.87 (0.00)	5.36 (0.980)	38.	6.5
RCM_5	0.224	0.030	123.87 (0.00)	8.55 (0.881)	38	7.8
RCM_6	0.224	0.030	123.87 (0.00)	10.22 (0.746)	38	10.3

NNM1 = One nearest neighbor matching and common support

NNM_2 = Two nearest neighbor matching and common support

KBM3 = Kernel with band width 0.05 and common support

KBM4 = Kernel with band width 0.1 and common support

RCM4 = Radius Caliper 0.05 matching

RCM5= Radius Caliper 0.1 matching

Table 8 shows the result of PSM, using three different matching algorithm techniques (nearest neighbor matching (NNM), Kernel based matching (KBM), and Radius matching methods). The result reveals that, as in the ESR analysis, the adoption of agricultural machine renting resulted in an increase in TFP, TE and COIM. The PSM result revealed that increased adoption of agricultural machines renting significantly increases total factor productivity of crops in the range of 0.41 to 0.52 (41 to 52 percent). Similarly, the adoption of agricultural machines increases technical efficiency from 0.077 to 0.104 (7.7% -10.4%). Moreover, on average, the adoption of agricultural machines increased the household's commercialization level in the range from 0.102 to 0.116 (10 to 11.6 percent). Therefore, it can be concluded that, apart from the minor differences in magnitude between the PSM and ESR estimates, the results of ESR and PSM indicate that the adoption of agricultural machines had positive impacts on TFP, TE and COIM levels.

Table 8.Average treatment effects: propensity score matching

Outcome variable	Matching algorism	Mean of outcome variables based on matched observation		ATE	ATT	SE
		Adopter	Non-adopter			
COMI						
	NNM_1	0.573	0.459	0.1105	0.114	0.015
	NNM_2	0.573	0.459	0.105	0.113	0.016
	KBM_3	0.573	0.46	0.105	0.113	0.016
	KBM_4	0.573	0.459	0.105	0.113	0.016
	RCM_5	0.555	0.456	0.098	0.100	0.013
	RCM_6	0.567	0.451	0.108	0.116	0.011
	NNM_1	3.10	2.57	0.46	0.524	0.104
TFP	NNM_2	3.10	2.6	0.44	0.49	0.102
	KBM_3	3.10	2.58	0.44	0.511	0.093
	KBM_4	310	2.58	0.45	0.513	0.090
	RCM_5	3.12	2.64	0.46	0.47	0.080
	RCM_6	3.11	2.68	0.39	0.411	0.066
	NNM_1	0.847	0.753	0.089	0.093	0.020
	NNM_2	0.847	0.745	0.089	0.102	0.017
TE	KBM_3	0.847	0.749	0.085	0.098	0.015
	KBM_4	0.847	0.751	0.84	0.096	0.015
	RCM_5	0.847	0.773	0.073	0.077	0.010
	RCM_6	0.847	0.774	0.070	0.077	0.009

4. SUMMARY

The agricultural sector in Ethiopia is the main source of economic development, and its growth originates from smallholder farmers. Several methodologies have been devised and applied, including the use of improved crop varieties to improve food production. However, the situation is still dire for a significant proportion of the population that is dependent on agriculture..Thus, this study analyzed the of impact agricultural mahines on the productivity and commercialization of cereal crops in West Gojjam, Ethiopia. A series of formal and informal surveys were carried out during the 2020/2021 crop season on 400 sample farm household heads that were randomly drawn from six kebeles in three woredas through a multi-stage sampling procedure. Secondary data were collected from published and unpublished sources to support the primary data.

Descriptive statistics and econometric models were used to analyze the data. The descriptive statistics include the mean and percentage of the demographic, socioeconomic and institutional characteristics of the sample farm households. Econometric models, including endogenous switch regression (ESR) and propensity score matching (PSM) were employed.

The descriptive statistics showed that approximately 48% sample of households were renting agricultural machines to perform different agricultural operations. From this, approximately 25.3%household heads rented tractors only for ploughing services and about 22.3% of farmers' rented combined harvesters for wheat harvesting, threshing, and winnowing operations. Similarly, about 28.54% rented maize sheller machines only for shelling of maize after harvesting. The study also investigates the total factor productivity and technical efficiency of cereal crops by using the total factor productivity index and stochastic frontier function. The results showed that the total factor index of the cereal crop was 2.89 and the stochastic frontier model showed that technical efficiency of cereal crop producers was 80.6%. The impact of agricultural machine renting on productivity and commercialization was estimated using endogenous switch regression and propensity score matching models. In these models, 192 machine users and 208 non-users of agricultural machines were used. The results of the endogenous switch regression and propensity score matching model indicate that the adoption of agricultural machines has positive and significant impacts on total factor productivity, technical efficiency and output commercialization index.

4.1. Policy implication

The adoption of agricultural machines has a positive and significant impact on the productivity and commercialization of cereal crops. Therefore, governmental and non-governmental stakeholders give more consideration to the selective and appropriate utilization of agricultural machines to increase productivity and commercialization. The government also develops appropriate mechanization technologies, strengthens extension services to provide training and technical assistance, fosters collaboration between stakeholders, and creats supportive policies and financial mechanisms to facilitate the adoption of mechanization by farmers of all sizes.

5. REFERENCE

1. World Bank Indicators Washington, DC, 2020. Author

2. CSA (Central Statistical Agency), Agricultural sample survey report on Area and production of major Crops (private peasant holdings, meher season 2016 / 2017; Addis Ababa Ethiopia, the FDRE statistical bulletin, Volume I.

3. Fisseha Z , Abrham S, and Dawit, A . Cereal production practices and technical efficiency among farm households in major "*teff*" growing mixed farming areasofEthiopia:*CogentEconomics&Finance,* 2022;10:1, doi: 10.1080/23322039.2021.2012986

4. Doss C. R, W. M. Mwangi, H. Verkuijl, and H, De Groote. Adoption of Maize and Wheat Technologies in Eastern Africa: A Synthesis of the Findings of 22 Case Studies. International Maize and Wheat Improvement Center (CIMMYT) Economics Working Paper Mexico City,2020, http://repository.cimmyt.org/handle/10883/1037

5. Shita A, Kumar N, and Singh S. Agricultural Technology Adoption and its Determinants in Ethiopia: A Reviewed Paper. *Asia Pacific Journal of Research,*I(LVV) 2018, 99–104

6. Macauley H. Feeding Africa: Cereal Crops: Rice, Maize, Millet, Sorghum, Wheat. 2015; Vol. 86, AbdouDiouf International Conference Centre, Senegal, 74.

7. CIMMYT . Farm mechanization and conservation Agriculture for sustainable intensification (FACASI) project.Market analysis for small mechanization Ethiopia,2014 .

8. Dagninet A, and Wolelaw E. Agricultural Mechanization: Assessment of Mechanization Impact Experiences on the Rural Population and the Implications for Ethiopian Smallholders. *Engineering and Applied Sciences*, 2016; 1(2): 39-48.

9. Houmy K.; M. F, Rojas, and C. Side,. Agri-hire in sub-Saharan Africa Business models or investing in sustainable mechanization Rome,. 2021, https://doi.org/10.4060/cb5071en

10. Berhane G, Kalle H, and Bart M. Agricultural Mechanization in Ethiopia: Evidence from the 2015 Feed the Future survey, International Food Policy Research Institute, 2016.

11. Feder G, Just R. E., & Zilberman D. Adoption of agricultural innovations in developing countries: A survey. *Economic development and cultural change*, 198;.*33*(2) :255-298

12. Adu-B F, Daum T., &Birner R. Can small farms benefit from big companies' initiatives to promote mechanization in Africa? A case study from Zambia. *Food policy,* 2019*; 84*, 133-145.

13. Kirui O. The Agricultural mechanization in Africa: micro-level analysis of state drivers and effects. *ZEF-Discussion Papers on Development Policy*, 2019 ; (272).

14. Yukichi Y, Mano Yukichi Y, Takahashi, K, Otsuka K. Contract Farming, Farm Mechanization, and Agricultural Intensification: the Case of Rice Farming in Cote d'Ivoire. Working Papers, 2017; vol. 157 JICA Research Institute.

15. Houssou N, andChapoto A. Adoption of farm mechanization, cropland expansion, and intensification in Ghana, 2015. (No. 1008-2016-80214).

16. Kahan D, Bymolt R, Zaal F. Thinking outside the plot: insights on small-scale mechanisation from case studies in east Africa. J. Dev. Stud, 2017;1–16.

17. Cossar F. Impact of mechanization on smallholder agricultural production: evidence from Ghana, 2019 (No. 2229-2019-1910).

18. Daum T, and Birner R. Agricultural mechanization in Africa: Myths, realities and an emerging research agenda. Global Food Security, 2020; 26, 100393. https://doi.org/10.1016/j.gfs.2020.100393

19. Berhane G, Dereje M, Minten B., and Tamru S. The rapid – but from a low base – uptake of agricultural mechanization in Ethiopia : Patterns, implications and challenges. *International Food Policy Research Institute* (IFPRI). ESSPWorkingPaper,2017; 105

20. Diao X, Cossar F, Houssou N, &Kolavalli S. Mechanization in Ghana: Emerging demand, and the search for alternative supply models. Food Policy, 2014.; 48, 168–181. https://doi.org/10.1016/j.foodpol.2014.05.013

21. Takeshima H. Market imperfections for tractor service provision in Nigeria: International perspectives and empirical evidence, 2016; 32. Intl Food Policy Res Inst.

22. FAO and AUC. Sustainable Agricultural Mechanization: A Framework for Africa. Addis Ababa, 2018; 127pp. Licence: CC BY-NC-SA 3.0 IGO

23. Rosegrant, M. W., Koo, J., Cenacchi, N., Ringler, C., Robertson, R., Fisher, M., Cox, C., Garrett, K., Perez, N. D., and Sabbagh, P. Food Security in a World of Natural Resource Scarcity the Role of Agricultural Technologies. Monographs of the Society for Research in Child Development, 2014; 79 (1)

24. Wudu B. Determinants of adoption of improved wheat technology: in case of Gozzamen district, east Gojjam in Amhara regional state, Ethiopia (thesis, St. Mary's University) ,2017.

25. Gebru A. The Determinants of Modern Agricultural Inputs Adoption and Their Productivity in Ethiopia (The Case of Amhara and Tigray Regions) (thesis, Addis Ababa University), 2016.

26. Worku A . Factors affecting diffusion and adoption of agricultural innovations among farmers in Ethiopia case study of Ormia regional state WestsernShewa. *International Journal of Agricultural Extension*, 2019;7(2):137–147. https://doi.org/10.33687/ijae.007.02.2648

27. Workineh M, Lakew T, and Haile K. Eshetie. Agricultural technology adoption and its impact on smallholder farmers welfare in Ethiopia. *African Journal of Agricultural Research*, 2020; 15(3): 431–445. https://doi.org/10.5897/AJAR2019.14302

28. Massresha E, Lema Z, Neway, and Degu A. Perception and determinants of agricultural technology adoption in north shoa zone, Amhara regional state, Ethiopia. *Cogent* Economics & Finance, 2021; 9(1), 1956774.https://doi.org/10.1080/23322039.2021.1956774

29. Tamrat G, Mengistu K, Arega S, and Getachew L. Determinants of farm mechanization in central and southeast oromia region, Ethiopia,2023. https://doi.org/10.1016/j.heliyon.2023.e18390

30. Hassena M, Regassa E, Mwangi H, Verkuijl H. A Comparative Assessment of Combine Harvesting Vis-à-vis Conventional Harvesting and Threshing in Arsi Region, Ethiopia. Mexico, D.F. (CIMMYT) and Ethiopia Agricultural Research Organization(EARO) , 2000.

31. Berhane G, Kalle H, & Bart, M. Agricultural Mechanization in Ethiopia: Evidence from the 2015 Feed the Future survey, International Food Policy Research Institute, 2016.

32. Tamirat N, and Abafita J. Adoption of row planting technology and household welfare in southern Ethiopia, in case of wheat grower farmers in Duna district, Ethiopia. *Asia-Pacific Journal of Science and Technology*, 2021; 26(2).

33. Bamidele F.S, Babatunde R.O, and Rasheed A. Productivity analysis of cassava-basede production systems in the Guinea Savannah: Case study of Kwara State, Nigeria. *American-Eurasian Journal of Scientific Research*, 2008; 3 (1): 33-39.

34. Key N. and Mcbride W. Production contracts and productivity in the US hog sector. *American Journal of Agricultural Economics*, 2003; 85 (1): 121-133

35. Ukoha O.O, Okoye B.C and Emetu J. Analysis of the Determinants of Total Factor Productivity among Small-Holder Cassava Farmers in Ohafia L.G.A of Abia State, 2010.

36. Von Braun J and Kennedy E. Agricultural Commercialization, Economic Development and Nutrition.The Johns Hopkins University Press, Baltimore and London,1994.

37. Strasberg, P Jayne, T. Yamano, T., Nyoro, J., Karanja.D and Strauss, J. Effects of agricultural commercialization on food crop input use and productivity in Kenya: Office of sustainable development; policy synthesis for USAID-Africa Bureau, 1999.

38. Berhanu G and Moti J. Commercialization of smallholders: Does market orientation translate into market participation? Improving Productivity and Market Success (IPMS) of Ethiopian farmer's project, working paper, 2010; 22: 30 p.

39. Coelli T.J. and G.E. Battesse. A Model of technical efficiency effects in stochastic frontier function for panel data. *Journal* of *Empirical Economics*, 1995; 20: 325-332.

40. Meeusen W. and van den Broeck J. Efficiency estimation from Cobb-Douglas production functions with composed error. *International Economic Review*, 1977; 18: 435-444.

41. KabungaN. S., Dubois, T., and Qaim, M. Yield effects of tissue culture bananas in Kenya: Accounting for selection Bias and the role of complementary inputs. *Journal ofAgricultural Economics*, 2012; 63:444–464. https://doi.org/10.1111/j.1477-9552.2012.00337.

42. Shiferaw B, Kassie M, Jaleta M,. and Yirga C. Adoption of improved wheat varieties and impacts on household food security in Ethiopia. *Food Policy*, 2014; 44:272–284. https://doi.org/10.1016/j.foodpol.2013.09.012

43. Ahmed M, Kassaun G, Amreo, T., and Eeden A. The impact of improved maize varieties on farm productivity and wellbeing: Evidence from the east Hararghe zone of Ethiopia. *Development Studies Research*, 2017; 4, 9–21. ..

44. Khonje M, Manda J, Alene A. D, & Kassie M.. Analysis of Adoption and Impacts of Improved Maize Varieties in Eastern Zambia. *World Development*, 2015; 66: 695- 706.

45. Maddala G.S. Limited dependent and qualitative variables in econometrics. Cambridge, U.K.: Cambridge University Press, 1983.

46. Maddala, G.S. and Nelson, F.D. Switching regression models with exogenous and endogenous switching. Proceeding of the American Statistical Association (Business and Economics Section), 1975; pp. 423-426.

47. Caliendo, M., and Kopeinig, S. 2008. Some practical guidance for the implementation of propensity score matching, Discussion paper No. 1588, University of Cologne.

48. Owusu V, and Awudu A. nonfarm Employment and Poverty Reduction in Rural Ghana: A Propensity-Score Matching Analysis, 2009. DOI 10.22004/ag.econ.51363

49. Nguezet P, Diagne,A, Okoruwa V, Ojohomon V. Impact of improved rice technology on income and poverty among rice farming household in Nigeria; contributed paper prepared for the 25th conference of the Centre for the Studies of African Economics, 2011.

50. Dehejia R, and Wahba S. Propensity score matching methods for non -experimental causal studies. The Review of Economics and Statistics, 2002; 84(1):151–161.

51. Asfaw D., & Neka M. Factors affecting adoption of soil and water conservation practices: The case of Wereillu Woreda, south Wollo zone, Amhara region, Ethiopia. International Soil and Water Conservation Research, . (2017); (5): 273-279.

53. Mesele Z, Abebaw H and Getamesay B. Determinants of multiple agricultural technology adoption: evidence from rural Amhara region, Ethiopia, *Cogent Economics & Finance*, 2022; 10:1, DOI: 10.1080/23322039.2022.2058189.

54. Etsehiwot S. The Determinants of Agricultural Technology Adoption and Its' Impact on teff productivitye in the Case of Amhara and Oromia National Regional State. Degree of Masters of Science in Economics A.A, Ethiopia, 2018..

55. Peng J, Zhao Z, and Liu D .. Impact of agricultural mechanization on agricultural production, income, and mechanism: evidence from hubei province, china. front. environ. sci, 2022; 10:838686. doi: 10.3389/fenvs.2022.838686

Click here to access/download
Supporting Information
Appendix.docx
