# Supplementary material for: Impacts of agricultural machine renting on cereal crop productivity and commercialization in West Gojjam Zone, Ethiopia
Source: PLoS One. 2025 Oct 27;20(10):e0300831. doi: 10.1371/journal.pone.0300831 (PMC12558508; doi:10.1371/journal.pone.0300831)
Supplement: Attachment — Submitted filename: PONE-D-24-08983_R1- Second review.pdf [file pone.0300831.s006.pdf]

PLOS ONE

Impacts of Agricultural Machine renting on Cereal Crop Productivity and Commercialization in West Gojjam Zone, Ethiopia.

--Manuscript Draft--

**Manuscript Number:**	PONE-D-24-08983R1
**Article Type:**	Research Article
**Full Title:**	Impacts of Agricultural Machine renting on Cereal Crop Productivity and Commercialization in West Gojjam Zone, Ethiopia.
**Short Title:**	Impacts of Agricultural Machine renting on Cereal Crop
**Corresponding Author:**	Selam Tilahun<br>Wolaita Sodo University<br>Sodo, ETHIOPIA
**Keywords:**	Agricultural machine, Productivity, Commercialization, Endogenous switch regression, Impact
**Abstract:**	This study investigated the impacts of agricultural machine renting on cereal crop productivity and commercialization. The study used cross-sectional data collected from 192 agricultural machine users and 208 non-users from West Gojjam zone, Northern Ethiopia. The data were analyzed using descriptive statistics and econometric models. The econometric model includes Trans log production function, Endogenous switch regression and propensity score matching. The result of descriptive statistics showed that the total factor productivity index of the cereal crop was 2.89, and the mean commercialization index for the sample households was .0.506.The estimation of results of Trans log production function model showed an average level of Technical Efficiency (TE) of 80.6%. the result of first stage Endogenous switch regression model showed that age, education level, ownership of oxen, total cultivated land, extension visit, access to information, family size, the position a farmer, and distance to the nearest market significantly affected adoption of agricultural machine renting (tractor, combine harvester and maize Sheller).The result of endogenous switching regression and propensity score matching models showed that agricultural machine renting had a positive and significant impact on the selected outcome variables, total factor productivity, technical efficiency and output commercialization index. Based on the findings, the study suggests that the government and stakeholders should focus on strengthening the provision of formal and informal education, development of infrastructures, extension service, to promote agricultural machine renting service, and enhance cereal crops productivity and commercialization.
**Order of Authors:**	Selam Tilahun
	Berhanu Kuma
	Amsalu Bedemo
**Response to Reviewers:**	We appreciate the constructive and insightful comments of both reviewers for their careful reading of the manuscript and their constructive remarks, on our manuscript entitled ‘‘Impacts of Agricultural Machine Renting on Cereal Crop Productivity and Commercialization in West Gojjam Zone, Ethiopia". We are pleased by the comment, input, and clarity raised by both reviewers’. We have taken the reviewers’ comments fully to incorporate, clarify, and improve our manuscript, which will meet the standard of the Plos ONE
**Additional Information:**	
**Question**	**Response**
**Financial Disclosure**<br><br>Enter a financial disclosure statement that describes the sources of funding for the work included in this submission. Review	The author(s) received no specific funding for this work.

Powered by Editorial Manager® and ProduXion Manager® from Aries Systems Corporation

the submission guidelines for detailed requirements. View published research articles from *PLOS ONE* for specific examples.<br><br>This statement is required for submission and **will appear in the published article** if the submission is accepted. Please make sure it is accurate.<br><br>**Funded studies**<br>Enter a statement with the following details:<br>• Initials of the authors who received each award<br>• Grant numbers awarded to each author<br>• The full name of each funder<br>• URL of each funder website<br>• Did the sponsors or funders play any role in the study design, data collection and analysis, decision to publish, or preparation of the manuscript?<br><br>Did you receive funding for this work?	
**Competing Interests**<br><br>Use the instructions below to enter a competing interest statement for this submission. On behalf of all authors, disclose any competing interests that could be perceived to bias this work—acknowledging all financial support and any other relevant financial or non-financial competing interests.<br><br>This statement is required for submission and **will appear in the published article** if the submission is accepted. Please make sure it is accurate and that any funding sources listed in your Funding Information later in the submission form are also declared in your Financial Disclosure statement.<br><br>View published research articles from *PLOS ONE* for specific examples.	The authors declare no conflict of interest.

**NO authors have competing interests**<br><br>Enter: *The authors have declared that no competing interests exist.*<br><br>**Authors with competing interests**<br><br>Enter competing interest details beginning with this statement:<br><br>*I have read the journal's policy and the authors of this manuscript have the following competing interests: [insert competing interests here]*<br><br>* typeset	
**Ethics Statement**<br><br>Enter an ethics statement for this submission. This statement is required if the study involved:<br><br>• Human participants<br>• Human specimens or tissue<br>• Vertebrate animals or cephalopods<br>• Vertebrate embryos or tissues<br>• Field research<br><br>Write "N/A" if the submission does not require an ethics statement.<br><br>General guidance is provided below. Consult the submission guidelines for detailed instructions. **Make sure that all information entered here is included in the Methods section of the manuscript.**	The ethical approval for the study was obtained from the “Wolaita Sodo University ethical board and College of agriculture” and the research was carried out based guidelines of Wolaita Sodo University ethics statement. Therefore, the study offered an approval number of 356/16 by the ethical committee. We also obtained a survey study titled "agricultural machine renting and its impact on cereal crop productivity and commercialization: the case of smallholder farmers in West Gojjam zone, Amhara region, Ethiopia". Informed consent was obtained from all individual participants included in the study who are voluntary to answer the questionnaires of the research. The consent was obtained through interview method then the response documented by interviewer in written format.

Powered by Editorial Manager® and ProduXion Manager® from Aries Systems Corporation

**Format for specific study types**<br><br>**Human Subject Research (involving human participants and/or tissue)**<br>• Give the name of the institutional review board or ethics committee that approved the study<br>• Include the approval number and/or a statement indicating approval of this research<br>• Indicate the form of consent obtained (written/oral) or the reason that consent was not obtained (e.g. the data were analyzed anonymously)<br><br>**Animal Research (involving vertebrate animals, embryos or tissues)**<br>• Provide the name of the Institutional Animal Care and Use Committee (IACUC) or other relevant ethics board that reviewed the study protocol, and indicate whether they approved this research or granted a formal waiver of ethical approval<br>• Include an approval number if one was obtained<br>• If the study involved *non-human primates*, add *additional details* about animal welfare and steps taken to ameliorate suffering<br>• If anesthesia, euthanasia, or any kind of animal sacrifice is part of the study, include briefly which substances and/or methods were applied<br><br>**Field Research**<br><br>Include the following details if this study involves the collection of plant, animal, or other materials from a natural setting:<br>• Field permit number<br>• Name of the institution or relevant body that granted permission	
**Data Availability**<br><br>Authors are required to make all data underlying the findings described fully available, without restriction, and from the time of publication. PLOS allows rare exceptions to address legal and ethical concerns. See the PLOS Data Policy and FAQ for detailed information.	Yes - all data are fully available without restriction

A Data Availability Statement describing where the data can be found is required at submission. Your answers to this question constitute the Data Availability Statement and **will be published in the article**, if accepted.<br><br>**Important:** Stating 'data available on request from the author' is not sufficient. If your data are only available upon request, select 'No' for the first question and explain your exceptional situation in the text box.<br><br>Do the authors confirm that all data underlying the findings described in their manuscript are fully available without restriction?	
**Describe where the data may be found in full sentences. If you are copying our sample text, replace any instances of XXX with the appropriate details.**<br><br>• If the data are **held or will be held in a public repository**, include URLs, accession numbers or DOIs. If this information will only be available after acceptance, indicate this by ticking the box below. For example: *All XXX files are available from the XXX database (accession number(s) XXX, XXX.)*.<br>• If the data are all contained **within the manuscript and/or Supporting Information files**, enter the following: *All relevant data are within the manuscript and its Supporting Information files.*<br>• If neither of these applies but you are able to provide **details of access elsewhere**, with or without limitations, please do so. For example:<br><br>*Data cannot be shared publicly because of [XXX]. Data are available from the XXX Institutional Data Access / Ethics Committee (contact via XXX) for researchers who meet the criteria for access to confidential data.*<br><br>*The data underlying the results presented in the study are available from (include the name of the third party*	All relevant data are within the paper and its Supporting Information files

and contact information or URL).<br>• This text is appropriate if the data are owned by a third party and authors do not have permission to share the data.<br><br>* typeset	
Additional data availability information:	Tick here if the URLs/accession numbers/DOIs will be available only after acceptance of the manuscript for publication so that we can ensure their inclusion before publication.

Manuscript Click here to access/download;Manuscript;Ploss.docx

Impacts of Agricultural Machine renting on Cereal Crop Productivity and Commercialization in West Gojjam Zone, Ethiopia.

SelamTilahun[a] ,BerhanuKuma[b] and AmsaluBedemo[c]

a. **Email:selam4998@gmail.com,**Department of Agricultural Economics, Wolaita Sodo University
b. **Email:berhanukuma@yahoo.com,** Department of Agricultural Economics, Wolaita Sodo, University
c. **Email:amsalu72@gmail.com,** Department of Economics, Addis Ababa, University

ABSTRACT

This study investigated the impacts of agricultural machine renting on cereal crop productivity and commercialization. The study used cross-sectional data collected from 192 agricultural machine users and 208 non-users from West Gojjam zone, Northern Ethiopia. The data were analyzed using descriptive statistics and econometric models. The econometric model includes Trans log production function, Endogenous switch regression and propensity score matching. The result of descriptive statistics showed that the total factor productivity index of the cereal crop was 2.89, and the mean commercialization index for the sample households was .0.506.The estimation of results of Trans log production function model showed an average level of Technical Efficiency of 80.6%. the result of first stage Endogenous switch regression model showed that age, education level, ownership of oxen, total cultivated land, extension visit, access to information, family size, the position a farmer, and distance to the nearest market significantly affected adoption of agricultural machine renting (tractor, combine harvester and maize Sheller).The result of endogenous switching regression and propensity score matching models showed that agricultural machine renting had a positive and significant impact on the selected outcome variables, total factor productivity, technical efficiency and output commercialization index. Based on the findings, the study suggests that the government and stakeholders should focus on strengthening the provision of formal and informal education, development of infrastructures, extension service, to promote agricultural machine renting service, and enhance cereal crops productivity and commercialization.

Keywords: Agricultural machine, Productivity, Commercialization, Endogenous switch regression, Impact

1. INTRODUCTION

Sub-Saharan Africa (SSA) is one of the regions in the world that is mainly characterized by smallholder farm households, where their livelihood depends primarily on agriculture. Similarly, Ethiopia's economy mainly depends on agriculture, which is the main source of income for 84% of the population, and it accounts about 33.8% of the country's Gross Domestic Product (GDP) and 70% of export earnings [1]. Cereal cultivation and marketing is the primary source of income for millions of subsistence farmers in the country. Cereals are accounts for 60 percent of rural employment in Ethiopia, 80% of cultivated land in the country, over 40% of food expenditure for the typical household, and over 60% of total calorie intake [2]. Cereals are the main food crop in terms of area coverage and production.

Cereal crop production and marketing have shown remarkable progress in Ethiopia, yet the domestic price for cereals is growing from time to time due to the rapid increase in domestic demand, rapid population growth, income, and urbanization [3]. Given the importance of cereal crops in national food security endeavors, smallholder farmers have used traditional methods of production with limited mechanization. Thus low productivity and, limited adoption of agricultural technologies including mechanizations and subsistence-based smallholder farming are characteristics of the sector [4,5]. Specifically, a low level of mechanization in agriculture continues to be the main obstacle to advancing cereal production, which, in turn, results in a high cost of production [6]. As a result, agricultural mechanization is among the mechanisms that increase production and productivity and reduce food insecurity [7].In the same way, according to [8] and [9] the utilization of appropriate agricultural mechanization technologies helps to increase the productivity and commercialization of cereal crops.

.

However, there is a public debates about the benefit of agricultural mechanization. According to the International Labor Organization (ILO, 1973) "mechanization leads to unemployment and is linked to economies of scale, meaning that it is more likely to be bought by larger farms [10,11]. In Africafew studies have conducted on impacts of mechanization on employment, and those that have mostly reported mixed effects [12,13,14,15] and positive effect [16,17]. According to

[18] the impacts of mechanization on employment depend on location-specific factors such as wages, land availability, and type of mechanization. On the other hand, smallholder farmers can take advantage of mechanization by renting markets and cooperative exchange [19,20,21].

Ethiopia's agriculture sector has depended on inexpensive and surplus labour for a long time. However, the condition is currently changing owning to the rapid migration of young people from rural to urban areas and the availability of universal access to education for all school-aged children [22,23]. This has led to a shortage of farm labor and pressure on the remaining household members. Additionally, the cost of oxen and lack of grazing land have become a major concern for smallholder farmers [19]. Consecquently, the use of machinery rental services has become a viable option for smallholder farmers. Recently, renting agricultural machines by smallholder farmers has been used in Ethiopia. Likewise, the Amhara region is one of the cereal crop growing areas and agriculture is the primary economic sector, but only 4.35% of agricultural activities oprate mechanically.

A number of studies have been conducted on the factors affecting the adoption of agricultural technology in Ethiopia; for example[24,25,26,27,28,29]. Only a few studies have been conducted on what may be the determinant of the adoption of the agricultural mechanization services with no specific studies on the agricultural machine renting services [30,31,32]. These previous studies applied logit, linear probability and tobit models to examine factors affecting agricultural mechanization. An important innovation in this paper was examining the effects of agricultural machine renting in Ethiopia on the productivity and commercialization of cereal crops. Hence there is no study on the impact of agricultural mechanization on productivity and commercialization which enables us to identify its impact, and helps to design an appropriate policy towards fostering the adoption of agricultural machines.

2. METHODOLOGY

2.1. Area Description

The study was carried out in the West Gojjam Zone of the Amhara region. West Gojjam zone, is one of the eleven (11) administrative zones established under the Amhara region. The administrative zone is bordered to the north by the Northern and Southern Gonder zones; to the east by East Gojjam zone; to the south by Oromia region and to the west by Benshangul Gumuz region. Figure 1 shows the study area of the research.

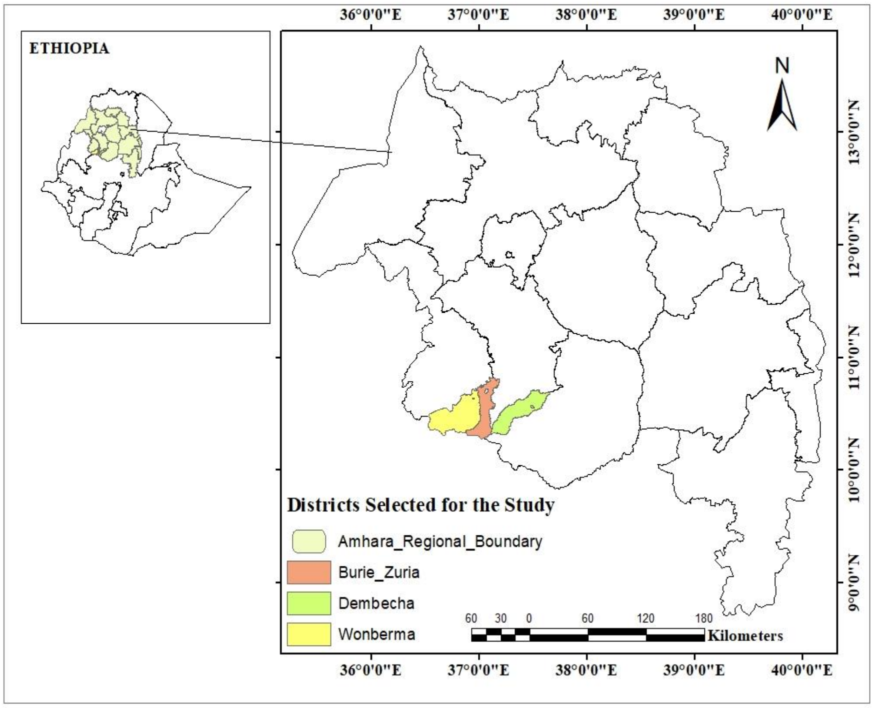

Figure 1: Map of the study area

Source:http://data.humdata.org/

2.2. Sampling Procedure and Sample Size Determination

A multi-step sampling procedure was used for the selection of the districts, the kebeles and the sample households. The first stage included the selection of three districts (wonberma, buriezuria and dembecha) from the areas where agricultural machine rental practices took place. The

second stage involved the selection of two randomly selected kebeles for each district. The households were divided into two groups: those who adopted agricultural machines and those who did not. The third stage involved randomly selecting 400 household heads. The 400 household head sample consisted of 121 households (wonberma), 123 households (buriezuria) and 156 households (dembecha district) from each strata. Proportional sampling was used to determine the number of households in each strata, taking into account the size of the district and the kebel. The final sample consisted of 192 households that had adopted at least one agricultural machine, 208 households that had not adopted an agricultural machine, were randomly selected from both strata using proportionate probability sampling based on the size of each district and kebel in the 2020/21 production season as shown Table 1

Cochran’s (1963) method was used determine the sample size. Cochran's method is particularly suitable for stratified sampling designs, where the population is divided into distinct subgroups or strata, and is beneficial when the population size is large or unknown. The equation is written as:

$$n = \frac{z^2\, pq}{d^2}\, (eq)(1)$$

where n is a minimum sample size, Z is 1.96 at 95 % confidence level; p is smallholder farmers who may adopt in agricultural machine (50 %); q is the weight variable and is computed as 1-p), and d is the desired precision or margin of error, expressed as a fraction of 0.05.

Table 1.Selected districts, kebeles and their sample sizes

District	Total hhs	Sampled Kebeles	Total hhs	Sample hhs	
Wenberma	18644	Markuma	1029	58	121
		Koki	1124	63	
Buriezuria	18929	Alefa	989	68	123
		Wadra	808	55	
Dembecha	23958	Wade	659	53	156
		Asteboch	1300	103	
Total	61756		7332	400	

2.3. Data Types and Sources

Both quantitative and qualitative data were used in this study. both secondary and primary data sources were used to generate data types. Primary data sources are farmers who rent agricultural machines and non-users of these machines. Secondary data sources include journals, books, CSA and internet browsing, national policies, and zonal, district and kebele reports.

2.4. Data Collection Methods

The major data collection methods included formal surveys, observations, visual aids, discussions with key informants and focus groups.The primary data were collected though face-to-face personal interviews using a semi-structured questionnaire. The data were collected based on the research objectives. The questionnaire included both open and closed-ended questions to collect information relevant to the scope of the study. The questionnaire was first tested prior to the field survey for suitability (clarity, suitability and sequence of questions) and then re-tested based on the pre-test feedback.. In addition to interviews, discussion with each of the six selected kebeles extension agents who are working related to agricultural mechanization service (mechanization experts) were disscussed.

2.5. Methods of Data Analyses

Two types of data analysis methods were used to addressthe rereasch objective: descriptive statistics and econometric models.

Measurement of cereal crop productivity

In this study, TFP is the ratio of output to the total variable cost (TVC). This approach Total Fixed Cost (TFC), does not role since it is constant and does not have any effects the on the profit maximization and resource-use efficiency conditions [33]. According to [34] and [35 TFP for cross-sectional data or data within a specified time can be measured as the inverse of the unit variable cost because TFP is the ratio of the output and the Total Variable Cost (TVC) as shown in this equation;

$$TFP = \frac{Y}{TVC} = \frac{\sum_{i=1}^{n} Y}{\sum_{j=1}^{n} PijXji} \qquad eq(2)$$

$$AVC = \frac{TVC}{Y}$$

$$TFP = \frac{Y}{TVC} = \frac{1}{AVC}$$

Where, Yi : The output of cereal crops (in value or in quantities);

TVCi: Total Variable Cost of farm i;

Pji: Unit price of jth variable input used in farm i;

Xji: Quantity of jth variable input used in farm i.

Measurement of market participation

Measuring Crop Output Market Participation Index (COMPI): The output side of commercialization (market participation) can be seen at the household or community level. At the household level, one definition of agricultural commercialization is defined as the ratio of the value of agricultural outputs sold to the total value of agricultural outputs produced by a household [36,37,38]. Hence, output side commercialization index or market participation for a household (COMPI) can be computed as:

$$COMPL_K = \sum_{i=1}^{N} \frac{S_{ki}}{Q_{ki}} \quad ; \quad Q_{ki} \geq S_{ki} \qquad \text{eq (3)}$$

Where, $COMPI_k$ is the proportion of crop k sold (S_{ki}) to the total amount produced (Q_{ki}) aggregated over the total sample households in a given location.

Efficiency and productivity analysis

The study used the production data of cereal crops (teff, wheat and maize) in the 2020/21 production season. The stochastic frontier methodology was later developed by[39,40]. It is specified as

$$y_i = f(x_{i,}\beta) + \varepsilon_i(v_i - u_i) \qquad eq(4)$$

Where: Yi is the amount of the cereal crops yield produced by ith household in the 2020/21 production year that is expressed in quintals; is a production function $f(x_{i,}\beta)$are the parameters to be estimated; X's are exogenous input factors (labour, land, fertilizer seed and herbicide) affecting the amount of yield; $\varepsilon i$is the error term, equal to ($Vi - Ui$); Vi is a two-sided random error component beyond the control of the farmer, and $Ui$is one-sided inefficiency component.

Given the level of input, the TE of the ith cereal crop producer farmers are the ratio of the observed output to the maximum potential output (frontier output):

$$TE_i = \frac{actual\ output}{potential\ output} = \frac{f(x_i;\beta)\exp(v_i - \mu_i)}{f(x_i;\beta)\exp(v_i)} = \exp(-\mu_i)$$

2.6. The impacts of agricultural machines renting on productivity and commercialization

To measure the impact of agricultural mechanization on the productivity and commercialization of cereal crops, endogenous switch regression (ESR) and propensity score matching (PSM) models were employed.

When assessing the impact of adopting an agricultural machine on the productivity commercialization of a farm household, it is important to capture both the observable and unobserved characteristics of the adopter (treatment group) as well as the non-adopter (control group). However, the majority of impact assessment methods that use non-experimental data do not capture the observable and/or non-observable characteristics that influence adoption as well as outcome variables. For example, instrumental variables only capture unobserved heterogeneity but assume that the parallel shift in outcome variables can be seen as a treatment effect [41,42,43].

The decision to adopt a technology can be modeled in a random utility framework by expressing the unobservable utility from adoption and non-adoption through observable variables[44].We specify the selection equation for technology adoption as:

$$\boldsymbol{P}_i^* = Zi\beta + U_i \qquad P = \begin{cases} 1, & if\ p* > 1 \\ 0, & otherwise \end{cases} \qquad eq(5)$$

Where$\boldsymbol{P}_i^*$: is unobservable or latent variable for machine renting, Yi is its observable counterpart (the dependent variable use of agricultural machines equals one, if the farmer has adopted at least one agricultural machine during 2020/21 cropping season, and zero otherwise), Zi are non-stochastic vectors of observed farm and non-farm characteristics determining adoption and Ui is random disturbances associated with renting of agricultural machine of improved technology.

The two regimes that the smallholder farmers fall in to are represented by the following two regression equations.

$$Regime\ 1: Y_I = \alpha_1 X_i + e_{1i} \qquad if\ p = 1 \qquad eq(6)$$

$$Regime\ 2: Y_2 = \alpha_2 X_i + e_{2i} \qquad if\ p = 0 \qquad eq(7)$$

Y1i and Y2i are the dependent outcome variables determined by the exogenous variables Xi, α1, and α2, are parameters that show the direction and strength of the relation between the outcome variable and the independent variables. e_{1i} ande_{2i} are error terms.

Finally, the error terms are assumed to have a trivariate normal distribution, with zero mean and non-singular covariance matrix expressed as:

$$cov(u_i, e_{1i}, e_{2i}) = \begin{bmatrix} \sigma^2 u & . & . \\ \sigma^2 e_1 u & \sigma^2 e_1 & . \\ \sigma^2 e_2 u & . & \sigma^2 e_2 \end{bmatrix} \qquad eq(8)$$

Where $\sigma^2 u$ variance of the error term in the selection equation, $\sigma^2 e_1$ and $\sigma^2 e_2$ are variances of the error terms in the continuous equations. $\sigma^2 e_1 u$and$\sigma^2 e_2 u$ are covariance of $u_i, and\ \ e_{1i}\ and\ e_{2i}$respectively. Since Y_{1i} and Y_{21} are not observed simultaneously a covariance of the corresponding error terms is not defined [45]. This structure of the error terms indicates

that the error terms of the outcome equation and the error term of the selection equation are correlated which results in non-zero expected value of e_{1i} and e_{2i} given ui - error term of the selection equation [46]. Therefore, the expected values of the truncated error terms E(e_1 | p = 1) and $E(e_2$ | p = 0) are given below:

E (e_1 | p = 1) = $E(e_1$ | U > - Zβ)

$$+= \sigma e_1 u \frac{\varphi(\frac{Z\beta}{\sigma})}{\Phi \frac{Z\beta}{\sigma}} = \sigma e_1 u \lambda_1 \qquad eq(9)$$

E (e_2 | p = 1) = $E(e_2$ | U ≤ - Zβ)

$$= \sigma e_1 u \frac{-\varphi(\frac{Z\beta}{\sigma})}{1 - \Phi(\frac{Z\beta}{\sigma})} = \sigma e_2 u \lambda_2 \qquad eq(10)$$

φ and Φ are the probability density and cumulative distribution function of the standard normal distribution, respectively. The ratio of φ and Φ evaluated at Zβ is referred to as the inverse Mills ratio $\lambda 1$ and $\lambda 2$ (selectivity terms). If the estimated covariance $\sigma^2 \varepsilon 1u$ and $\sigma^2 \varepsilon 2u$ are significantly different from 0 the decision to renting agricultural machine and the outcome variable (productivity and commercialization) are correlated. This implies endogenous switching and the presence of a sample selectivity bias [47]. Further, estimations of treatment effects will be made. Average Treatment effect on the Treated and Untreated (ATT and ATU) are computed using the results for expected values of the dependent variable for users and non-users in actual and counterfactual scenarios:

$$E(Y_{1i}|p_i = 1, X_{1i}) = \alpha_1 X_{1i} + \sigma e_1 u \rho_1 \frac{\varphi(z\beta)}{\Phi(z\beta)} \qquad eq(11)$$

$$E(Y_{2i}|p_i = 0, X_{2i}) = \alpha_2 X_{2i} - \sigma e_2 u \rho_2 \frac{\varphi(z\beta)}{(1 - \Phi(z\beta)} \qquad eq(12)$$

$$E(Y_{2i}|p_i = 1, X_{1i}) = \alpha_2 X_{1i} + \sigma e_2 u \rho_2 \frac{\varphi(z\beta)}{\Phi(z\beta)} \qquad eq(13)$$

$$E(Y_{1i}|p_i = 0, X_{2i}) = \alpha_2 X_{2i} - \sigma e_1 u \rho_2 \frac{\varphi z\beta}{(1 - \Phi(z\beta)} \qquad eq(14)$$

ATT is the difference between the expected value of the outcome variable from Equations 11 and 13; this is the difference between the expected value of the dependent variable for users and if they were not used. ATU is the difference between Equations 12 and 14 which estimates the difference between the expected value of the outcome variable for non-users of agricultural machine.

PSM techniques were applied to supplement the ESR model and to assess the consistency of the results with different assumptions such as nearest neighbour matching, caliper and radius matching, and kernel and local linear matching [48]. The treatment effects will be estimated based on matching estimators selected on the common support region [49]. The average treatment effects can be estimated using the inverse propensity weighing estimates as stated in IPSW [50] using matching techniques of Kernel Matching (KM), Nearest Neighbor Matching (NNM) and Radius Caliper Matching (RCM).

The selection of a particular approach depends on the data in question and, in particular, the degree of similarity between the treatment and control groups in terms of propensity score [51].The Average Treatment Effect of an individual i can be written as:

$$\mathrm{ATE} = \mathrm{E}(\mathrm{Y}_\mathrm{i}^1|\mathrm{D}_\mathrm{i} = 1) - \mathrm{E}\left(\mathrm{Y}_\mathrm{i}^0 \,\middle|\, \mathrm{D}_\mathrm{i} = 0\right) \qquad \mathrm{eq}(15)$$

Where ATE, Average Treatment Effect, which is the effect of treatment on the outcome variables:

$E(Yi^1 | Di=1)$: Average outcomes for individual, with user, if he/she would participant
$E (Yi^0 | Di=0)$: Average outcome of non-user, when he/she would non-participant

The dependent variable:

Adoption of agricultural machine renting: A dichotomous dependent variable measured in participation of agricultural machine renting, taking value 1 if there is participation in agricultural machine renting and 0 otherwise.

Outcome variable

Total factor productivity: Total factor productivity index of cereal crops includes (teff, wheat and maize) produced by households in the 2020/21 production year.

Technical efficiency: Technical efficiency of cereal crops (teff, wheat and maize) in the 2020/21 production season.

Output commercialization: Cereal crop output commercialization indices include teff, wheat and maize output commercialization indicesin the 2020/21 production season.

The independent variables: the explanatory variables that were assumed to affect both the participation of households in renting machines and the outcome variable are age, family size, level of education, total cultivated land credit use, frequency of extension contact, participation in off-farm activity, distance to the nearest market, distance from road, distance from DA office, position of the farmer in farmer's association, ownership oxen, livestock and membership of cooperative.

3. RESULTS AND DISCUSSION

3.1. Demographic, Socio-Economic and Institutional Characteristics

Table 2 shows the statistical reports of the demographic, socio-economic and institutional characteristics of the sampled households. The age of the sample households ranged from 24 to 70 years, with an average age of 44.718 years. Moreover, the mean typical household attended approximately 4 years of formal education. The selected households owned on average four livestock in TLU, with a minimum of none and a maximum of twelve, excluding the ownership of oxen. The mean total land holding of households was about 2.14ha with a minimum of 0.25 and a maximum of 5 hectares. In addition, the mean own land holding of the sampled household was 1.45 hectares. Households in the study area have an average of six members with a mean total dependency ratio of 0.49.

Table2.Demographic, socio-economic and institutional characteristics for continuous variable

Variable	Obs	Mean	Std. Dev.	Min	Max
Age of hh	400	44.75	9.64	24	70
Education level	400	3.78	3.08	0	12
Farming experience	400	20.05	9.68	1	48
Family size	400	6.12	1.69	1	12
distance to market	400	3.19	2.39	0.1	16
Distance to main road	400	2.37	1.54	0.1	7
Distance to DA	400	2.29	1.36	.15	8
Extension visit	400	11.03	6.21	1	30
Total land	400	2.14	0.92	0.25	5
Ownership of oxen	400	2.39	0.97	0	6
TLU	400	4.00	2.06	0	12
Own land	400	1.44	0.77	0	4

Source: Computed from survey data (2021)

The sex of the sample of farm household heads, about 94.75% were male-headed. The result indicated that about 43.25% of the households used credit services for different purposes. As shown in Table 3 below, about 79.25% of the sampled household heads were members of cooperatives, and about 49.25% of the sampled respondents also heldpositions in social activities (edir ,equib and position on agricultural activities).

Table 3. Demographic, socio-economic and institutional characteristics for categorical variable

Variable	Description	No of households(n=400)	Percent
Sex of household head	Male	379	94.75
	Female	21	5.25
Position social participation	Yes	195	48.75
Credit use	Yes	173	43.25
Membership of cooperative	Yes	317	79.25
Off -farm participation	Yes	131	32.75
Machine renting	Yes	192	48
access information EA	Yes	282	70.5

Source: Computed from survey data (2021)

3.2 Agricultural machine renting

In the study area, tractors, combine harvestesrs and maize Sheller are used in the production process of cereal crops, either solely or simultaneously. The study showed that Table 4 approximatly 25.3% rented tractors only for ploughing services. Farmers commonly rent tractors in the study area for the first and second tillage. This is becauset the first ploughing operation is the heaviest and most challenging for oxen, whereas a tractor can plough easily and deeply. Similarly, about 22.3% of farmers rented combined harvesters only for wheat harvesting, threshing and winnowing operation and about 28.54% rented maize Sheller machines only for shelling of maize after harvesting. About 11.16% of farm households rent three types of machines (tractor, combine harvester and maize Sheller) simultaneously.

Table 4.Major agricultural machine used in the study area

Types of agricultural machines used	Freq.	Percent
Tractor	118	25.3
Combiner	104	22.3
Sheller	133	28.54
All (tractor, combiner and Sheller)	52	11.16
Tractor &combiner	17	3.65
Tractor & Sheller	20	4.3
Combiner & Sheller	22	4.72

3.3. Impacts of agricultural machine renting on productivity and commercialization

Table 5 represents the first stage ESR binary probit estimation results. The probit model fits the data reasonably well [Wald Chi-squared = 137.31, p= 0.000)]. The model results reaveled that household, socioeconomic, and institutional factors had a significant impact on the decision to rent agricultural machines.

The age of the household head affected adoption of agricultural machine renting positively and significantly at 1% level. The marginal effect result indicates that aged household heads adopt more agricultural machines than younger households because, as the age of households increases, working power decreases. Agricultural machine renting increased with the level of education of the household heads. Household heads who attened better education level have a good awareness about agricultural machines. The total land cultivated significantly and positively affected the adoption of agricultural machines. Farmers who owned large amount of cultivated land were more likely to adopt agricultural machines than small land-holding farmers. Ownership of large amounts of cultivated land often required more labor and time to perform agricultural operations.

Frequency of extension contact influenced the adoption of agricultural machines significantly and positively at the 1% level. This implies that farmers who are frequently in contact with extension agents are more likely to adopt agricultural machines than are those in lower contact with extension agents. This is because, development agents provide advice on agricultural machine utilization and benefits to farmers. The extension provides in-depth information, training and consultancy services on the source, application and relevance of agricultural technologies for farmers [52]. Similarly, farmers with information about agricultural machines from government extension agents are more likely to adopt agricultural machines. This is because the provision of information helps farmers to become aware of the benefits and costs of machines. The use of credit negatively and significantly affected the probability of farmers using agricultural machines to produce cereal crops at 1%. The result indicated that farmers who used

credit services decreased the probability of agricultural machines renting. This may be because households used credit for consumption, fattening livestock and home construction.

Distance from the market and the main road negatively and significantly affected the adoption of agricultural machines by 10% and 5%, a significant level. Proximity to the market and main roads are important factor in adopting agricultural machines because better access to markets and roads is an indicator of access to information and infrastructure. Thus, farmers who live near the market and main road are more likely to rent agricultural machines and other important inputs at a lower cost than those who live far away from markets and main roads. The number of oxen affects the adoption of agricultural machines positively and significantly at 1%. This implies an increase in the number of oxen and the probability of adopting agricultural machines increases. This means that households with many oxen perform agricultural activities like the second plow, third plow, sowing and threshing more easily than thos fewer oxen.

The results of the second stage ESR and,the estimated coefficients of total factor productivity, technical efficiency and commercialization index are presented in the appendix.

Table 5. Decision of adopting agricultural machines renting: Probit model

Variables	Coeff	Std.Err	Marginal Effect
Age of household	0.03***	0.009	0.012
Education level of household	0.09***	0.026	0.036
Adult equivalent	-0.11	0.059	-0.045
Poweron social activity	-0.49***	0.149	-0.196
Market distance	-0.07*	0.043	-0.031
Road distance	0.14**	0.073	0.058
Distance from office	0.1271	0.086	-0.050
Extension visit	0.0376***	0.012	0.015
Member of cooperative	0.2692	0.185	0.107
Credit use	-0.4276***	0.146	-0.170
Total land	0.3655***	0.040	0.145
TLU	-0.0372	0.041	-0.014
Number of oxen	0.3934***	0.110	0.156
Off farm activities	-0.0666	0.158	-0.026
Information for mechanization	0.3425**	0.165	0.136
-Cons-	-2.1487***	0.705	

Source: Computed from survey data (2021)

Table 6 shows the results ATT and ATU for the key outcome variables related to agricultural machine renting. In this analysis the main outcome vaiables are TFP, TE and COIM index. Before, we analyzed the result of ESR model to check the validity of instrumental variables using a falsification test. The falsification test shows the instrumental variable significantly affects the adoption of agricultural machines but not TFP, TE and COIM index. Instrument variables are jointly statistically significant in the adoption equation [$\chi 2$ = 4.22 (p = 0.04)] but not outcome functions. The instrumental variable did not affect the TFP [F=0.10(p= 0.7497)] technical efficiency [F=0.45 (p=0.5038)], and commercialization index [F= 1.92 (p=0.1668)].

The adoption of agricultural machines was renting increases total productivity, technical efficiency and commercialization level of farm households. For farmers who adopted agricultural machine renting but theoretically would have not adopted, then their TFP decreased by 0.573 (57.3%). This means, the ATT of total factor productivity of cereal crops would decrease by 57.3% as a result of did not use agricultural machines renting. Likewise, if non-users of agricultural machine adopted agricultural machine renting, then their TFP would have

significantly increased by 0.419(41.9%). For farmers who adopted agricultural machines but theoretically would not have adopted them, then their TE decreased by 0.104 (10.4). This means, the ATT of TE on cereal crops would decrease by 10.4% due to do not adopting agricultural machines. Similarly, if non-users of agricultural machine adopted agricultural machines, then their TE would have significantly increased by 0.064(6.4%). In addition, the results of the commercialization index of cereal crops for adopters would drop by 0. 12(12%) if the adopters were not adopted. In the same way, if the non-users of agricultural machine had adopted agricultural machine renting, their COIM would have increased by 0.098 (9.8%).

Consequently, ESR shows that in Ethiopia the adoption of agricultural machine renting would have increased productivity and commercialization of cereal crops. This result resembles to [53] the adoption of agricultural technology has a positive effect on crop productivity including wheat. The adoption of agricultural technologies has also a positive and significant impact on productivity of teff [54] The adoption of agricultural mechanization has a significant positive impact on the productivity of all types of crops [55].

Table. 6 Endogenous switching regression model result (average treatment effects)

Outcome variables	Mean farm household type and treatment effect	Decision stage		Treatment effect
		To Adopt	Not to Non-adopt	
Productivity	ATT	3.1	2.52	0.573***
	ATU	3.13	2.7	0.422***
TE	ATT	0.845	0.741	0.104***
	ATU	0.837	0.772	0.064***
COIM	ATT	0.572	0.452	0.120***
	ATU	0.544	0.444	0.099***

Note. ATT = Adoption effect for adopters, ATU = Adoption effect for non-adopters, and *** p <.01.

Popensity Score Matching Estimation Results

In addition to the ESR model, this study uses the PSM technique to check the robustness of the results obtained from the ESR model. Propensity scores (the probability of adoption in agricultural machine) are estimated using a probit model. Figure2 shows the distribution of adopter and non-adopter households with respect to estimated propensity scores. The figure illustrates the estimated propensity distribution for treatment and control households. The upper half of the graph refers to the propensity score distribution of treatment groups, while the bottom half shows the control groups. The y-axis refers to the densities of estimated propensity scores.

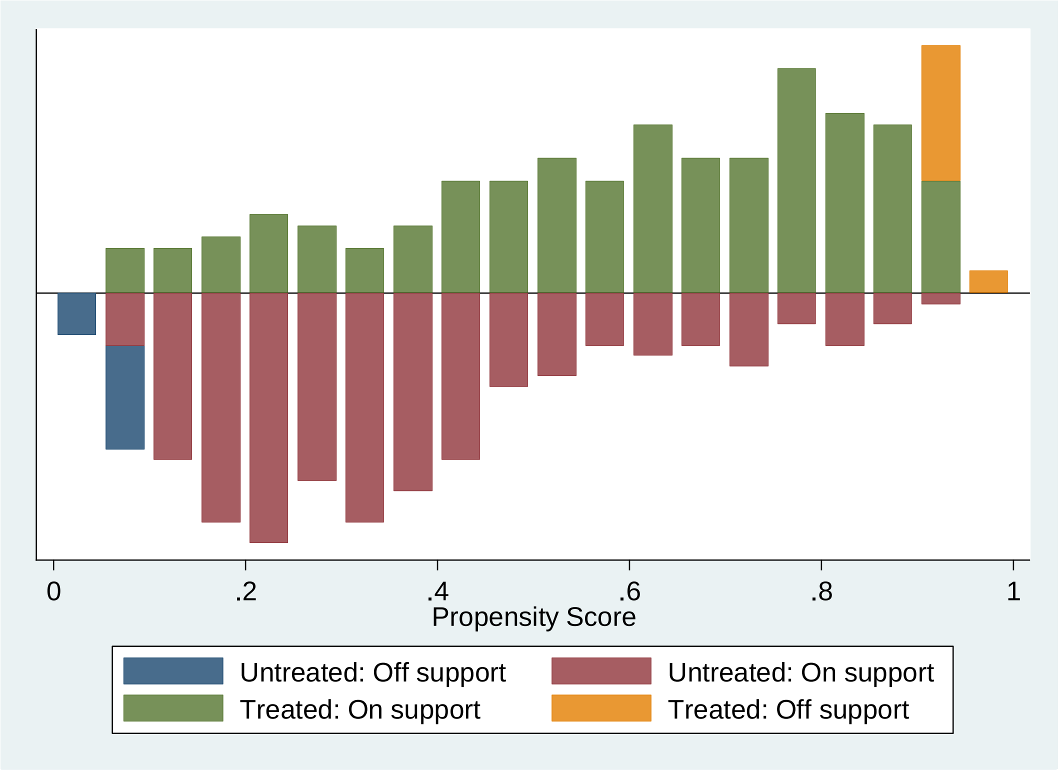

Figure 2: Distribution of estimated propensity distribution for treatment and control groups and common support area

There should be a common support condition that should be applied to how the propensity score is distributed between adopters of agricultural machine renting and those who do not. The

estimated propensity scores varied between 0.0826423 and 0.9633814 (mean =0. 6249002) and 0. 0347458 and 0.92732443 (mean, =0.3462459) in the treatment and control groups, respectively. The common support lies between 0.0826423 and 0.92732443. Table 7 presents the balancing tests for each matching algorithm before and after matching. The results show that the mean standardized bias is reduced after matching from 10.3 to 5.3 percent) compared to before matching (38percent). Similarly, Pseudo-R^2 declined substantially, from 22.4 percent to 1 percent. The p-values from the likelihood ratio tests showed that all of the covariates were statistically significant at a p-value of less than 1 percent prior to matching, but insignificant post-match. These p-values clearly demonstrate that the matching process balance the observed characteristics between treated and control after matching.

Table 7.Covariates balancing tests before and after matching

Matching algorism	Pesido-R-square		LRx^2P −value		Mean standard bias	
	Before	After	Before	After	Before	After
NNM_1	0.224	0.010	123.87<br>(0.00)	4.99<br>(0.986)	38	5.3
NNM_2	0.224	0.014	123.87<br>(0.00)	6.71<br>(0.278)	38.	6.9
KBM_3	0.224	0.010	123.87<br>(0.00)	4.83<br>(0.729)	38	6.6
KBM_4	0.224	0.011	123.87<br>(0.00)	5.36<br>(0.980)	38.	6.5
RCM_5	0.224	0.030	123.87<br>(0.00)	8.55<br>(0.881)	38	7.8
RCM_6	0.224	0.030	123.87<br>(0.00)	10.22<br>(0.746)	38	10.3

NNM1 = One nearest neighbor matching and common support

NNM_2 = Two nearest neighbor matching and common support

KBM3 = Kernel with band width 0.05 and common support

KBM4 = Kernel with band width 0.1 and common support

RCM4 = Radius Caliper 0.05 matching

RCM5= Radius Caliper 0.1 matching

Table 8 shows the result of PSM, using three different matching algorithm techniques (nearest neighbor matching (NNM), Kernel based matching (KBM), and Radius matching methods). The result reveals that, as in the ESR analysis, the adoption of agricultural machine renting resulted in an increase in TFP, TE and COIM. The PSM result revealed that increased adoption of agricultural machines renting significantly increases total factor productivity of crops in the range of 0.41 to 0.52 (41 to 52 percent). Similarly, the adoption of agricultural machines increases technical efficiency from 0.077 to 0.104 (7.7% -10.4%). Moreover, on average, the adoption of agricultural machines increased the household's commercialization level in the range from 0.102 to 0.116 (10 to 11.6 percent). Therefore, it can be concluded that, apart from the minor differences in magnitude between the PSM and ESR estimates, the results of ESR and PSM indicate that the adoption of agricultural machines had positive impacts on TFP, TE and COIM levels.

Table 8.Average treatment effects: propensity score matching

Outcome variable	Matching algorism	Mean of outcome variables based on matched observation		ATE	ATT	SE
		Adopter	Non-adopter			
COMI						
	NNM_1	0.573	0.459	0.1105	0.114	0.015
	NNM_2	0.573	0.459	0.105	0.113	0.016
	KBM_3	0.573	0.46	0.105	0.113	0.016
	KBM_4	0.573	0.459	0.105	0.113	0.016
	RCM_5	0.555	0.456	0.098	0.100	0.013
	RCM_6	0.567	0.451	0.108	0.116	0.011
	NNM_1	3.10	2.57	0.46	0.524	0.104
TFP	NNM_2	3.10	2.6	0.44	0.49	0.102
	KBM_3	3.10	2.58	0.44	0.511	0.093
	KBM_4	310	2.58	0.45	0.513	0.090
	RCM_5	3.12	2.64	0.46	0.47	0.080
	RCM_6	3.11	2.68	0.39	0.411	0.066
	NNM_1	0.847	0.753	0.089	0.093	0.020
	NNM_2	0.847	0.745	0.089	0.102	0.017
TE	KBM_3	0.847	0.749	0.085	0.098	0.015
	KBM_4	0.847	0.751	0.84	0.096	0.015
	RCM_5	0.847	0.773	0.073	0.077	0.010
	RCM_6	0.847	0.774	0.070	0.077	0.009

4. SUMMARY

The agricultural sector in Ethiopia is the main source of economic development, and its growth originates from smallholder farmers. Several methodologies have been devised and applied, including the use of improved crop varieties to improve food production. However, the situation is still dire for a significant proportion of the population that is dependent on agriculture..Thus, this study analyzed the of impact agricultural mahines on the productivity and commercialization of cereal crops in West Gojjam, Ethiopia. A series of formal and informal surveys were carried out during the 2020/2021 crop season on 400 sample farm household heads that were randomly drawn from six kebeles in three woredas through a multi-stage sampling procedure. Secondary data were collected from published and unpublished sources to support the primary data.

Descriptive statistics and econometric models were used to analyze the data. The descriptive statistics include the mean and percentage of the demographic, socioeconomic and institutional characteristics of the sample farm households. Econometric models, including endogenous switch regression (ESR) and propensity score matching (PSM) were employed.

The descriptive statistics showed that approximately 48% sample of households were renting agricultural machines to perform different agricultural operations. From this, approximately 25.3%household heads rented tractors only for ploughing services and about 22.3% of farmers' rented combined harvesters for wheat harvesting, threshing, and winnowing operations. Similarly, about 28.54% rented maize sheller machines only for shelling of maize after harvesting. The study also investigates the total factor productivity and technical efficiency of cereal crops by using the total factor productivity index and stochastic frontier function. The results showed that the total factor index of the cereal crop was 2.89 and the stochastic frontier model showed that technical efficiency of cereal crop producers was 80.6%. The impact of agricultural machine renting on productivity and commercialization was estimated using endogenous switch regression and propensity score matching models. In these models, 192 machine users and 208 non-users of agricultural machines were used. The results of the endogenous switch regression and propensity score matching model indicate that the adoption of agricultural machines has positive and significant impacts on total factor productivity, technical efficiency and output commercialization index.

4.1. Policy implication

The adoption of agricultural machines has a positive and significant impact on the productivity and commercialization of cereal crops. Therefore, governmental and non-governmental stakeholders give more consideration to the selective and appropriate utilization of agricultural machines to increase productivity and commercialization. The government also develops appropriate mechanization technologies, strengthens extension services to provide training and technical assistance, fosters collaboration between stakeholders, and creats supportive policies and financial mechanisms to facilitate the adoption of mechanization by farmers of all sizes.

5. REFERENCE

1. World Bank Indicators Washington, DC, 2020. Author

2. CSA (Central Statistical Agency), Agricultural sample survey report on Area and production of major Crops (private peasant holdings, meher season 2016 / 2017; Addis Ababa Ethiopia, the FDRE statistical bulletin, Volume I.

3. Fisseha Z , Abrham S, and Dawit, A . Cereal production practices and technical efficiency among farm households in major "*teff"* growing mixed farming areasofEthiopia:*CogentEconomics&Finance,* 2022;10:1, doi: 10.1080/23322039.2021.2012986

4. Doss C. R, W. M. Mwangi, H. Verkuijl, and H, De Groote. Adoption of Maize and Wheat Technologies in Eastern Africa: A Synthesis of the Findings of 22 Case Studies. International Maize and Wheat Improvement Center (CIMMYT) Economics Working Paper Mexico City,2020, http://repository.cimmyt.org/handle/10883/1037

5. Shita A, Kumar N, and Singh S. Agricultural Technology Adoption and its Determinants in Ethiopia: A Reviewed Paper. *Asia Pacific Journal of Research,*I(LVV) 2018, 99–104

6. Macauley H. Feeding Africa: Cereal Crops: Rice, Maize, Millet, Sorghum, Wheat. 2015; Vol. 86, AbdouDiouf International Conference Centre, Senegal, 74.

7. CIMMYT . Farm mechanization and conservation Agriculture for sustainable intensification (FACASI) project.Market analysis for small mechanization Ethiopia,2014 .

8. Dagninet A, and Wolelaw E. Agricultural Mechanization: Assessment of Mechanization Impact Experiences on the Rural Population and the Implications for Ethiopian Smallholders. *Engineering and Applied Sciences,* 2016; 1(2): 39-48.

9. Houmy K.; M. F, Rojas, and C. Side,. Agri-hire in sub-Saharan Africa Business models or investing in sustainable mechanization Rome,. 2021, https://doi.org/10.4060/cb5071en

10. Berhane G, Kalle H, and Bart M. Agricultural Mechanization in Ethiopia: Evidence from the 2015 Feed the Future survey, International Food Policy Research Institute, 2016.

11. Feder G, Just R. E., & Zilberman D. Adoption of agricultural innovations in developing countries: A survey. *Economic development and cultural change,* 198;.*33*(2) :255-298

12. Adu-B F, Daum T., &Birner R. Can small farms benefit from big companies' initiatives to promote mechanization in Africa? A case study from Zambia. *Food policy*, 2019; *84*, 133-145.

13. Kirui O. The Agricultural mechanization in Africa: micro-level analysis of state drivers and effects. *ZEF-Discussion Papers on Development Policy*, 2019 ; (272).

14. Yukichi Y, Mano Yukichi Y, Takahashi, K, Otsuka K. Contract Farming, Farm Mechanization, and Agricultural Intensification: the Case of Rice Farming in Cote d'Ivoire. Working Papers, 2017; vol. 157 JICA Research Institute.

15. Houssou N, andChapoto A. Adoption of farm mechanization, cropland expansion, and intensification in Ghana, 2015. (No. 1008-2016-80214).

16. Kahan D, Bymolt R, Zaal F. Thinking outside the plot: insights on small-scale mechanisation from case studies in east Africa. J. Dev. Stud, 2017;1–16.

17. Cossar F. Impact of mechanization on smallholder agricultural production: evidence from Ghana, 2019 (No. 2229-2019-1910).

18. Daum T, and Birner R. Agricultural mechanization in Africa: Myths, realities and an emerging research agenda. Global Food Security, 2020; 26, 100393. https://doi.org/10.1016/j.gfs.2020.100393

19. Berhane G, Dereje M, Minten B., and Tamru S. The rapid – but from a low base – uptake of agricultural mechanization in Ethiopia : Patterns, implications and challenges. *International Food Policy Research Institute* (IFPRI). ESSPWorkingPaper,2017; 105

20. Diao X, Cossar F, Houssou N, &Kolavalli S. Mechanization in Ghana: Emerging demand, and the search for alternative supply models. Food Policy, 2014.; 48, 168–181. https://doi.org/10.1016/j.foodpol.2014.05.013

21. Takeshima H. Market imperfections for tractor service provision in Nigeria: International perspectives and empirical evidence, 2016; 32. Intl Food Policy Res Inst.

22. FAO and AUC. Sustainable Agricultural Mechanization: A Framework for Africa. Addis Ababa, 2018; 127pp. Licence: CC BY-NC-SA 3.0 IGO

23. Rosegrant, M. W., Koo, J., Cenacchi, N., Ringler, C., Robertson, R., Fisher, M., Cox, C., Garrett, K., Perez, N. D., and Sabbagh, P. Food Security in a World of Natural Resource Scarcity the Role of Agricultural Technologies. Monographs of the Society for Research in Child Development, 2014; 79 (1)

24. Wudu B. Determinants of adoption of improved wheat technology: in case of Gozzamen district, east Gojjam in Amhara regional state, Ethiopia (thesis, St. Mary's University) ,2017.

25. Gebru A. The Determinants of Modern Agricultural Inputs Adoption and Their Productivity in Ethiopia (The Case of Amhara and Tigray Regions) (thesis, Addis Ababa University), 2016.

26. Worku A . Factors affecting diffusion and adoption of agricultural innovations among farmers in Ethiopia case study of Ormia regional state WestsernShewa. *International Journal of Agricultural Extension*, 2019;7(2):137–147. https://doi.org/10.33687/ijae.007.02.2648

27. Workineh M, Lakew T, and Haile K. Eshetie. Agricultural technology adoption and its impact on smallholder farmers welfare in Ethiopia. *African Journal of Agricultural Research*, 2020; 15(3): 431–445. https://doi.org/10.5897/AJAR2019.14302

28. Massresha E, Lema Z, Neway, and Degu A. Perception and determinants of agricultural technology adoption in north shoa zone, Amhara regional state, Ethiopia. *Cogent* Economics & Finance, 2021; 9(1), 1956774.https://doi.org/10.1080/23322039.2021.1956774

29. Tamrat G, Mengistu K, Arega S, and Getachew L. Determinants of farm mechanization in central and southeast oromia region, Ethiopia,2023. https://doi.org/10.1016/j.heliyon.2023.e18390

30. Hassena M, Regassa E, Mwangi H, Verkuijl H. A Comparative Assessment of Combine Harvesting Vis-à-vis Conventional Harvesting and Threshing in Arsi Region, Ethiopia. Mexico, D.F. (CIMMYT) and Ethiopia Agricultural Research Organization(EARO) , 2000.

31. Berhane G, Kalle H, & Bart, M. Agricultural Mechanization in Ethiopia: Evidence from the 2015 Feed the Future survey, International Food Policy Research Institute, 2016.

32. Tamirat N, and Abafita J. Adoption of row planting technology and household welfare in southern Ethiopia, in case of wheat grower farmers in Duna district, Ethiopia. *Asia-Pacific Journal of Science and Technology*, 2021; 26(2).

33. Bamidele F.S, Babatunde R.O, and Rasheed A. Productivity analysis of cassava-basede production systems in the Guinea Savannah: Case study of Kwara State, Nigeria. *American-Eurasian Journal of Scientific Research*, 2008; 3 (1): 33-39.

34. Key N. and Mcbride W. Production contracts and productivity in the US hog sector. *American Journal of Agricultural Economics*, 2003; 85 (1): 121-133

35. Ukoha O.O, Okoye B.C and Emetu J. Analysis of the Determinants of Total Factor Productivity among Small-Holder Cassava Farmers in Ohafia L.G.A of Abia State, 2010.

36. Von Braun J and Kennedy E. Agricultural Commercialization, Economic Development and Nutrition.The Johns Hopkins University Press, Baltimore and London,1994.

37. Strasberg, P Jayne, T. Yamano, T., Nyoro, J., Karanja.D and Strauss, J. Effects of agricultural commercialization on food crop input use and productivity in Kenya: Office of sustainable development; policy synthesis for USAID-Africa Bureau, 1999.

38. Berhanu G and Moti J. Commercialization of smallholders: Does market orientation translate into market participation? Improving Productivity and Market Success (IPMS) of Ethiopian farmer's project, working paper, 2010; 22: 30 p.

39. Coelli T.J. and G.E. Battesse. A Model of technical efficiency effects in stochastic frontier function for panel data. *Journal* of *Empirical Economics*, 1995; 20: 325-332.

40. Meeusen W. and van den Broeck J. Efficiency estimation from Cobb-Douglas production functions with composed error. *International Economic Review*, 1977; 18: 435-444.

41. KabungaN. S., Dubois, T., and Qaim, M. Yield effects of tissue culture bananas in Kenya: Accounting for selection Bias and the role of complementary inputs. *Journal ofAgricultural Economics*, 2012; 63:444–464. https://doi.org/10.1111/j.1477-9552.2012.00337.

42. Shiferaw B, Kassie M, Jaleta M,. and Yirga C. Adoption of improved wheat varieties and impacts on household food security in Ethiopia. *Food Policy*, 2014; 44:272–284. https://doi.org/10.1016/j.foodpol.2013.09.012

43. Ahmed M, Kassaun G, Amreo, T., and Eeden A. The impact of improved maize varieties on farm productivity and wellbeing: Evidence from the east Hararghe zone of Ethiopia. *Development Studies Research*, 2017; 4, 9–21. ..

44. Khonje M, Manda J, Alene A. D, & Kassie M.. Analysis of Adoption and Impacts of Improved Maize Varieties in Eastern Zambia. *World Development*, 2015; 66: 695- 706.

45. Maddala G.S. Limited dependent and qualitative variables in econometrics. Cambridge, U.K.: Cambridge University Press, 1983.

46. Maddala, G.S. and Nelson, F.D. Switching regression models with exogenous and endogenous switching. Proceeding of the American Statistical Association (Business and Economics Section), 1975; pp. 423-426.

47. Caliendo, M., and Kopeinig, S. 2008. Some practical guidance for the implementation of propensity score matching, Discussion paper No. 1588, University of Cologne.

48. Owusu V, and Awudu A. nonfarm Employment and Poverty Reduction in Rural Ghana: A Propensity-Score Matching Analysis, 2009. DOI 10.22004/ag.econ.51363

49. Nguezet P, Diagne,A, Okoruwa V, Ojohomon V. Impact of improved rice technology on income and poverty among rice farming household in Nigeria; contributed paper prepared for the 25th conference of the Centre for the Studies of African Economics, 2011.

50. Dehejia R, and Wahba S. Propensity score matching methods for non -experimental causal studies. The Review of Economics and Statistics, 2002; 84(1):151–161.

51. Asfaw D., & Neka M. Factors affecting adoption of soil and water conservation practices: The case of Wereillu Woreda, south Wollo zone, Amhara region, Ethiopia. International Soil and Water Conservation Research, . (2017); (5): 273-279.

53. Mesele Z, Abebaw H and Getamesay B. Determinants of multiple agricultural technology adoption: evidence from rural Amhara region, Ethiopia, *Cogent Economics & Finance*, 2022; 10:1, DOI: 10.1080/23322039.2022.2058189.

54. Etsehiwot S. The Determinants of Agricultural Technology Adoption and Its' Impact on teff productivitye in the Case of Amhara and Oromia National Regional State. Degree of Masters of Science in Economics A.A, Ethiopia, 2018..

55. Peng J, Zhao Z, and Liu D .. Impact of agricultural mechanization on agricultural production, income, and mechanism: evidence from hubei province, china. front. environ. sci, 2022; 10:838686. doi: 10.3389/fenvs.2022.838686

Manuscript

Click here to access/download;Manuscript;clear version of revised manuscript.docx

Impacts of Agricultural Machine Renting on Cereal Crop Productivity and Commercialization in West Gojjam Zone, Ethiopia.

SelamTilahun[a] ,BerhanuKuma[b] and AmsaluBedemo[c]

a. Email:selam4998@gmail.com,Department of Agricultural Economics, Wolaita Sodo University
b. Email:berhanukuma@yahoo.com, Department of Agricultural Economics, Wolaita Sodo, University
c. Email:amsalu72@gmail.com, Department of Policy Studies, Ethiopian Civil Service University

ABSTRACT

Agricultural mechanization plays an essential role to increase production, productivity and commercialization of cereal crops. Despite the Ethiopian's government has made substantial efforts to increase production, productivity and commercialization of cereal crops by the introduction of selective and adaptable agricultural machines to smallholder farmers has remained low. This study. therefore, investigated the impacts of agricultural machine renting on cereal crop productivity and commercialization. The study used cross-sectional data collected from 192 agricultural machine users and 208 non-users from West Gojjam zone, Ethiopia. The data were analyzed using descriptive statistics and econometric models. The econometric model includes Trans log production function, Endogenous switch regression and propensity score matching. The result of descriptive statistics showed that the total factor productivity index of the cereal crop was 2.89, and the mean commercialization index for the sample households was 0.506. The estimation of results of Trans log production function model showed an average level of Technical Efficiency of 80.6%. The result of first stage Endogenous switch regression model showed that age, education level, ownership of oxen, total cultivated land, extension visit, access to information, family size, the position a farmer, and distance to the nearest market significantly affected adoption of agricultural machine renting. The result of endogenous switching regression and propensity score matching models showed that agricultural machine renting had a positive and significant impact on the selected outcome variables, total factor productivity, technical efficiency and output commercialization index. Based on the findings, the study suggests that the government and stakeholders should focus on strengthening the provision of education, development of infrastructures, extension service, to promote agricultural machine renting service, and enhance cereal crops productivity and commercialization.

Keywords: Agricultural machine, Productivity, Commercialization, Endogenous switch regression, Impact

1. INTRODUCTION

Sub-Saharan Africa (SSA) is one of the regions in the world that is mainly characterized by smallholder farm households, where their livelihood depends primarily on agriculture. Similarly, Ethiopia's economy mainly depends on agriculture, which is the main source of income for 84% of the population, and it accounts about 33.8% of the country's Gross Domestic Product (GDP) and 70% of export earnings [1]. Cereal cultivation and marketing is the primary source of income for millions of subsistence farmers in the country. Cereals are accounts for 60 percent of rural employment in Ethiopia, 80% of cultivated land in the country, over 40% of food expenditure for the typical household, and over 60% of total calorie intake [2]. Cereals are the main food crop in terms of area coverage and production.

Cereal crop production and marketing have shown remarkable progress in Ethiopia, yet the domestic price for cereals is growing from time to time due to the rapid increase in domestic demand, rapid population growth, income, and urbanization [3]. Given the importance of cereal crops in national food security endeavors, smallholder farmers have used traditional methods of production with limited mechanization. Thus low productivity and, limited adoption of agricultural technologies including mechanizations and subsistence-based smallholder farming are characteristics of the sector [4,5]. Specifically, a low level of mechanization in agriculture continues to be the main obstacle to advancing cereal production, which, in turn, results in a high cost of production [6]. As a result, agricultural mechanization is among the mechanisms that increase production and productivity and reduce food insecurity [7].In the same way, according to [8] and [9] the utilization of appropriate agricultural mechanization technologies helps to increase the productivity and commercialization of cereal crops.

.

However, there is a public debates about the benefit of agricultural mechanization. According to the International Labor Organization (ILO, 1973) "mechanization leads to unemployment and is linked to economies of scale, meaning that it is more likely to be bought by larger farms [10,11]. In Africafew studies have conducted on impacts of mechanization on employment, and those that have mostly reported mixed effects [12,13,14,15] and positive effect [16,17]. According to [18] the impacts of mechanization on employment depend on location-specific factors such as

wages, land availability, and type of mechanization. On the other hand, smallholder farmers can take advantage of mechanization by renting markets and cooperative exchange [19,20,21].

Developing and intensifying the cereal crops production has been the core of Ethiopia's agricultural development strategies and investments in the last 20 years to address the problem of low agricultural production and productivity [3] Even though, still now, most of the interventions focused on the use of modern agronomic practice to improve agricultural productivity and production [22]. As a result, the problem has been continued with scanty achievement [8]. Many scholars state that the problem of low productivity is mainly related to low technical efficiency [23,24, 3]. Thus, high productivity can be achieved by using appropriate agricultural machines with agronomic practice in the right manner [25] Consequently, using appropriate and selective agricultural machines is the best strategy to increase production and productivity of crops, commercialization, food security [26, 9].

Ethiopia's agriculture sector has depended on inexpensive and surplus labour for a long time. However, the condition is currently changing owning to the rapid migration of young people from rural to urban areas and the availability of universal access to education for all school-aged children [27,28]. This has led to a shortage of farm labor and pressure on the remaining household members. Additionally, the cost of oxen and lack of grazing land have become a major concern for smallholder farmers [19]. Consecquently, the use of machinery rental services has become a viable option for smallholder farmers. Recently, renting agricultural machines by smallholder farmers has been used in Ethiopia. Similarly, the Amhara region is one of the cereal crop growing areas and agriculture is the primary economic sector, but only 4.35% of agricultural activities oprate mechanically, which is far below the national average [29]. Likewise, West Gojjam zone is the major cereal crop production area across the nation [30]. However, utilization of agricultural machine with in this area remains minimal with farming practice relying on age-old and tradtional implements. Moreover, in recent times there has been increase in the population and a significant rise in average labor wage. This has lead to an increase the demand for agricultural machine renting among in small farmers in West Gojjam zone [31].

A number of studies have been conducted on the factors affecting the adoption of agricultural technology in Ethiopia; for example[32,33,34,35,36,37]. Only a few studies have been conducted on what may be the determinant of the adoption of the agricultural mechanization services with no specific studies on the agricultural machine renting services [38,19,39]. These previous studies applied logit, linear probability and tobit models to examine factors affecting agricultural mechanization. An important innovation in this paper was examining the impacts of agricultural machine renting on the productivity and commercialization of cereal crops. Hence there is no study on the impact of agricultural mechanization on productivity and commercialization. As As result, this study provides information on smallholder farmers' agricultural machine renting and its impact on cereal crops productivity and commercialization which helps to draw a clear picture for policymakers involved in the development and dissemination of agricultural mechanization**.**

2. LITRATURE REVIEW

2.1 Theorthical Review

Agricultural mechanization is encompassed within the broader concepts of agricultural transformation and the development of farming systems. According to [40,41], the demand for mechanized technology by farmers is a consequence of the process of agricultural intensification. [41] is well-known for establishing a theoretical and empirical connection between population growth, technological innovation, and agricultural intensification. In order to promote agricultural intensification, new technologies are developed and implemented, such as increased use of inputs like labor and fertilizer, as well as the integration of non-human sources of power such as animal traction or machinery.

Boserup's discussion of mechanization does not extend to consider the source of mechanized technology, nor the relative prices of land, labour, and outputs. The only effort in existing literature to conceptualize the circumstances under which farmers opt for mechanized technology, rather than agricultural intensification in general, is seen in the application of the induced innovation hypothesis by [42]. The demand for labor- or land-intensive technology by farmers is influenced by the relative costs of land, labor, capital, and output, which encourages public or private research institutions to innovate and adjust their development of new technologies to meet the needs of farmers.

2.2. Empirical Review

Different studies have been conducted on the determinants of the adoption of agricultural technology and its impact on productivity, cost of production and households' welfare. However, there is no specific study about the impacts of agricultural mechanization on crop productivity in Ethiopia.

According to a study regarding the effect of farm mechanization on productivity of rice farms in southern Ghana, the productivity of rice differs depending on the type of mechanization intensity. In this particular study, households who used mechanization from tillage, threshing, and transportation had the most significant and positive relationship with productivity. Moreover, land size cultivated, agrochemical expenditure, tillage intensity, education and transportation were significant factors that positively influenced productivity of rice [43]. [44]

assessed the impact of farm mechanization on the productivity of wheat and maize crops in Pesh Awar valley. The result of Cobb-Douglas production function analysis revealed if the land of wheat and maize increases by one hectare, the productivity increases by 25.32 mounts for mechanized farmers to 23.22 mounds for non - mechanized farmers.

[13] examines the state, drivers and, consequently, the impacts of agricultural mechanization in eleven countries in Africa. Significant drivers of agricultural mechanization include the size of the household, gender of the household head, participation in off-farm economic activities, distance to the input and output markets, farm size, land tenure, type of farming system, access to extension services, and use of fertilizer and pesticides. His result showed that in general agricultural mechanization significantly raises the productivity of maize and rice in all cases. Similarly, [45] studied the impact of agricultural mechanization on agricultural production, income, and mechanism: evidence from Hubei province, China. This study analyzed the influence of agricultural mechanization level on agricultural production and income by utilizing a sample-modified endogenous merging model and a threshold effect model. The level of mechanization has a significant positive impact on the cost, output value, and income and return rate of all types of crops. For every 1% increase in the level of mechanization, the yields of all crops, grain crops and cash crops increase by 1.2, 1.56 and 0.43%, respectively.

2.3. Conceptual Framework

[40 and 41] explain agricultural intensification process needs mechanized technology, which is basically motivated by agro-ecological conditions, population density, and market demand. According to [20] demand for mechanization rose due to exogenous pushing factors of agricultural intensification, rising rural wages, urbanization, population pressure. Besides, rapid migration of the youth from rural to urban areas has a consequence of shortage of farm labor and cost draft animals changing the pattern of farming practice [27,19] Availability of machinery service provider in the nearby area of smallholder farmers is among the first factors that are contributing for making renting decision. Internal factors such as demographic and socio-economic and external factor like population growth, technological change, and development of infrastructure, market institutions and policy and are assumed to determine the renting decision of farmers for mechanization services.

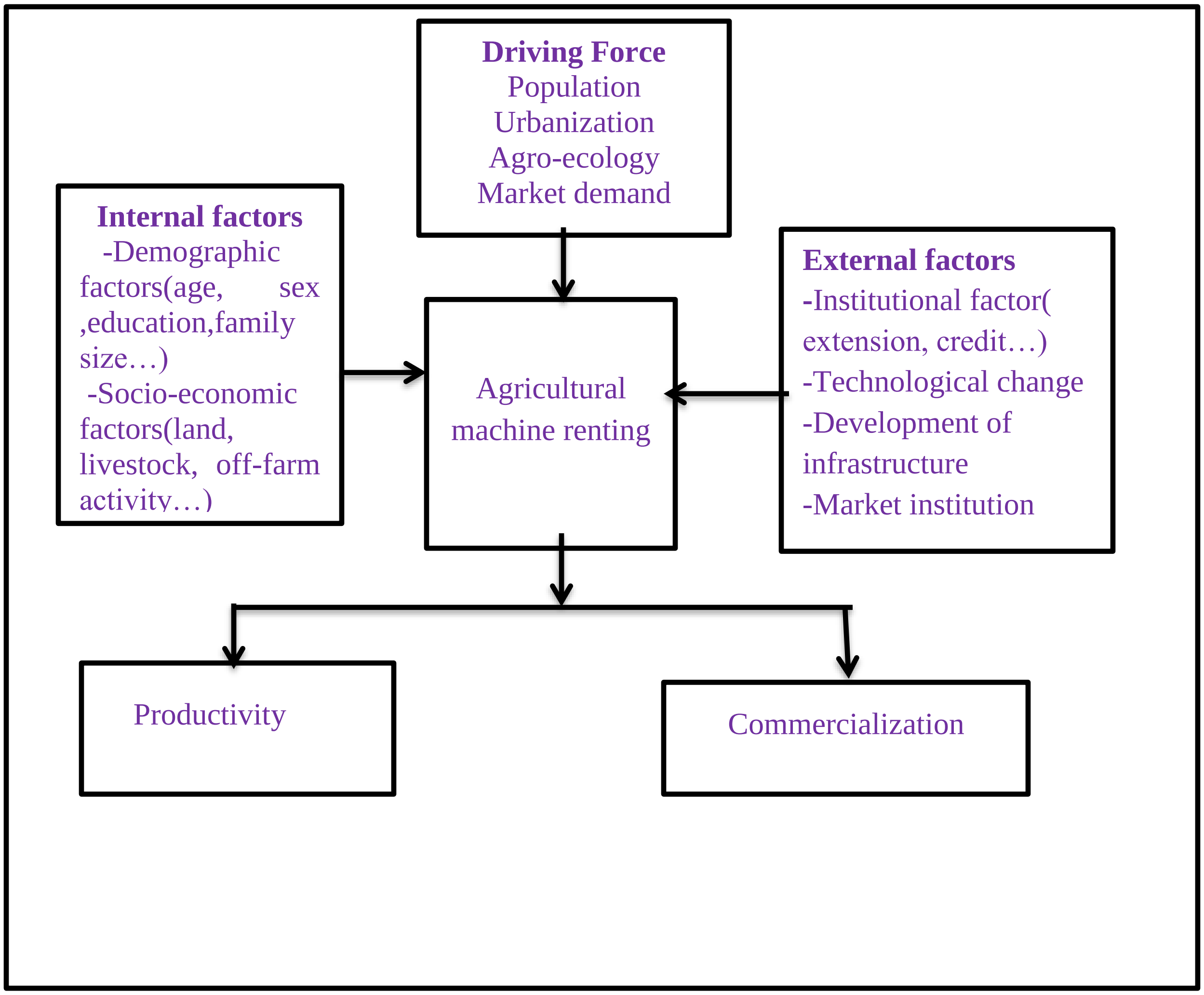

Source: own sketch

Figure 1: Conceptual framework

3. METHODOLOGY

3.1. Area Description

The study was carried out in the West Gojjam Zone of the Amhara region. West Gojjam zone, is one of the eleven (11) administrative zones established under the Amhara region. The administrative zone is bordered to the north by the Northern and Southern Gonder zones; to the east by East Gojjam zone; to the south by Oromia region and to the west by Benshangul Gumuz region. Agriculture system in West Gojjam zone is a combination of both crop and animal farming activities .the primary crops caltitivated in the area include teff, wheat,maize , pepper, fingermillet, bareley beans peas chiechpeas,among others.In the region cearals occupy 84.41% of the total agricultural land (637552 hectares) and account for approximately 89% the total crop yield(19,580,577.34) quintals.

In the West Gojjam Zone, farmers use different agricultural machines for ploughing, harrowing harvesting and threshing through renting agricultural machines. The agricultural machine renting service (tractor, combine harvester, maize Sheller) was started in 1980 and the number of agricultural machines used by farmers increased from time to time. These machine renting services are provided by a union, an agricultural mechanization corporation and privately through communication and agreement with machine owners from the Oromiya region. The total number of agricultural machines in the west Gojjam zone in the union and agricultural mechanization corporation are 84, 10 and 180 tractors, combine harvesters, maize Sheller respectively. Agricultural machine renting payment was different according to the situation due to road, distance to the market (station) and season etc. An average fee for ploughing, harvesting (threshing and winnowing), especially for wheat, and shelling for maize, was 3500 birr/ha, 130-150 birr/qt, and 20-30 birr/qt respectively [46]

3.2. Sampling Procedure and Sample Size Determination

A combination of purposive and multi-stage random sampling procedure was used to select districts, kebeles and sample households. Three districts from West Gojjam zone (Wonberma, Buriezuria and Dembecha) were selected purposively from the areas where agricultural machine rental practices took place. Then, in the first stage, a total of six kebeles were selected by simple random sampling method based on proportional to the number of Kebeles in each district. In the second stage households were stratified into two groups: those that adopted agricultural machines renting and those who did not. In the third stage, a representative sample size of 400 household heads were selected from both strata through systematic random sampling based on proportionate probability size of each districts and kebeles (121 households from Wonberma, 123 households from Burie Zuria and 156 households from Dembecha district)). Finally, 192 households that adopted at least one agricultural machine renting and 208 households that did not adopt an agricultural machine renting were obtained from six kebeles in the 2020/21 production season.as shown Table 1.

[47] method was used determine the sample size. Cochran's method is particularly suitable for stratified sampling designs, where the population is divided into distinct subgroups or strata, and is beneficial when the population size is large or unknown. The equation is written as:

$$n = \frac{z^2\, pq}{d^2}\,(eq)(1)$$

where n is a minimum sample size, Z is 1.96 at 95 % confidence level; p is smallholder farmers who may adopt in agricultural machine (50 %); q is the weight variable and is computed as 1-p), and d is the desired precision or margin of error, expressed as a fraction of 0.05.

Table 1.Selected districts, kebeles and their sample sizes

District	Sampled Kebeles	Total hhs	Sample hhs
Wenberma	Markuma	1029	69
	Koki	1124	76
Buriezuria	Alefa	989	67
	Wadra	808	55
Dembecha	Wade	659	45
	Asteboch	1300	88
Total		5909	400

3.3. Ethics Approval

The ethical approval for the study was obtained from the “Wolaita Sodo University ethical board and College of agriculture” and the research was carried out based guidelines of Wolaita Sodo University ethics statement. Therefore, the study offered an approval number of 356/16 by the ethical committee. We also obtained a survey study titled "agricultural machine renting and its impact on cereal crop productivity and commercialization: the case of smallholder farmers in West Gojjam zone, Amhara region, Ethiopia". Informed consent was obtained from all individual participants included in the study who are voluntary to answer the questionnaires of the research. The consent was obtained through interview method then the response documented by interviewer in written format.

3.4. Data Types and Sources

Both quantitative and qualitative data were used in this study. both secondary and primary data sources were used to generate data types. Primary data sources are farmers who rent agricultural machines and non-users of these machines. Secondary data sources include journals, books, CSA and internet browsing, national policies, and zonal, district and kebele reports.

3.5. Data Collection Methods

The major data collection methods included formal surveys, discussions with key informants and focus groups.The primary data were collected though face-to-face personal interviews using a semi-structured questionnaire. The data were collected based on the research objectives. The questionnaire included both open and closed-ended questions to collect information relevant to the scope of the study. The questionnaire was first tested prior to the field survey for suitability (clarity, suitability and sequence of questions) and then re-tested based on the pre-test feedback.. In addition to interviews, discussion with each of the six selected kebeles extension agents who are working related to agricultural mechanization service (mechanization experts) were disscussed.

3.6. Methods of Data Analyses

Two types of data analysis methods were used to addressthe rereasch objective: descriptive statistics and econometric models.

Descriptive statistics

Descriptive analysis was used to analyze demographic, institutional and socio-economic characteristics of farm households. Further, descriptive statistics such as mean, proportion and standard deviation were used to describe economic and physical characteristics associated with households categorized based on their agricultural machine renting of mechanization behaviors. The independent sample t-test and chi-square tests were used to make comparisons between the categories with respect to key variables specified.

Measurement of cereal crop productivity

In this study, TFP is the ratio of output to the total variable cost (TVC). This approach Total Fixed Cost (TFC), does not role since it is constant and does not have any effects the on the profit maximization and resource-use efficiency conditions [48]. According to [49] and [50] TFP for cross-sectional data or data within a specified time can be measured as the inverse of the unit variable cost because TFP is the ratio of the output and the Total Variable Cost (TVC) as shown in this equation;

$$TFP = \frac{Y}{TVC} = \frac{\sum_{i=1}^{n} Y}{\sum_{j=1}^{n} PijXji} \qquad eq(2)$$

$$ATFP = \frac{Y}{TVC} = \frac{1}{AVC}$$

Where, Yi : The output of cereal crops (in value or in quantities);

TVCi: Total Variable Cost of farm i;

Pji: Unit price of jth variable input used in farm i;

Xji: Quantity of jth variable input used in farm i.

Measurement of market participation

Measuring Crop Output Market Participation Index (COMPI): . Agricultural commercialization is the proportion of agricultural production that is marketed and the households that sell more proportions of his/her product could be considered as commercialized farmer [51]. The meaning of commercialization goes beyond supplying output product to the market and it has to consider both the input and output sides of production [52]. In line with this, smallholder commercialization occur when farmers participate in agricultural markets either as a buyer (input) and/or seller (output) [53]. In this paper focused on output side of commercialization (market participation). Many empirical researches conducted on commercialization level crops [54,55,56,39]. At the household level, one definition of agricultural commercialization is defined as the ratio of the value of agricultural outputs sold to the total value of agricultural outputs produced by a household [57,58,59]. Hence, output side commercialization index or market participation for a household (COMPI) can be computed as:

$$COMPL_K = \sum_{i=1}^{N} \frac{S_{ki}}{Q_{ki}} \quad ; \quad Q_{ki} \geq S_{ki} \qquad \text{eq (3)}$$

Where, $COMPI_k$ is the proportion of crop k sold (S_{ki}) to the total amount produced (Q_{ki}) aggregated over the total sample households in a given location.

Efficiency and productivity analysis

The study used the production data of cereal crops (teff, wheat and maize) in the 2020/21 production season. The stochastic frontier methodology was later developed by[60,61]. It is specified as

$$y_i = f\left(x_{i,}\beta\right) + \varepsilon_i(v_i - u_i) \qquad eq(4)$$

Where: Yi is the amount of the cereal crops yield produced by ith household in the 2020/21 production year that is expressed in quintals; is a production function $f(x_{i,}\beta)$are the parameters to be estimated; X's are exogenous input factors (labour, land, fertilizer seed and herbicide)

affecting the amount of yield; $\varepsilon i$is the error term, equal to ($Vi - Ui$); Vi is a two-sided random error component beyond the control of the farmer, and $Ui$is one-sided inefficiency component.

Given the level of input, the TE of the ith cereal crop producer farmers are the ratio of the observed output to the maximum potential output (frontier output):

$$TE_i = \frac{actual\ output}{potential\ output} = \frac{f(x_i;\beta)\exp(v_i - \mu_i)}{f(x_i;\beta)\exp(v_i)} = \exp(-\mu_i)$$

3.7. The impacts of agricultural machines renting on productivity and commercialization

To measure the impact of agricultural mechanization on the productivity and commercialization of cereal crops, endogenous switch regression (ESR) and propensity score matching (PSM) models were employed.

When assessing the impact of adopting an agricultural machine on the productivity commercialization of a farm household, it is important to capture both the observable and unobserved characteristics of the adopter (treatment group) as well as the non-adopter (control group). However, the majority of impact assessment methods that use non-experimental data do not capture the observable and/or non-observable characteristics that influence adoption as well as outcome variables. For example, instrumental variables only capture unobserved heterogeneity but assume that the parallel shift in outcome variables can be seen as a treatment effect [62,63,64].

In contrast, using regression models to analyze the impact of a given technology using pooled samples of users and non-users might be inappropriate because it gives the similar effect on both groups [64]. A methodological approach that overcomes the aforementioned limitations is ESR, which is the most frequently used common method to analyze the impact of a given technology

[65,64,66] In this paper, we employ parametric ESR with non-parametric PSM technique to reduce the selection bias and assure consistent results by capturing both the observed and unobserved heterogeneity that influence the outcome variable as well as the adoption decision.

The decision to adopt a technology can be modeled in a random utility framework by expressing the unobservable utility from adoption and non-adoption through observable variables[67].We specify the selection equation for technology adoption as:

$$\boldsymbol{P_i^*} = Zi\beta + U_i \qquad P = \begin{cases} 1, & if\ p* > 1 \\ 0, & otherwise \end{cases} \qquad eq(5)$$

Where$\boldsymbol{P_i^*}$: is unobservable or latent variable for machine renting, Yi is its observable counterpart (the dependent variable use of agricultural machines equals one, if the farmer has adopted at least one agricultural machine during 2020/21 cropping season, and zero otherwise), Zi are non-stochastic vectors of observed farm and non-farm characteristics determining adoption and Ui is random disturbances associated with renting of agricultural machine of improved technology.

Farmers use at least one of the agricultural machines (tractor, combine harvester, and maize Sheller) in their farming activities. However, according [66] if the selection equation (first stage) is endogenous in the outcome equation (second stage), the result would be biased and inefficient. Therefore, it is vital to use instrumental variable methods to identify the second stage equation from the first stage equation. The instrumental variable should affect the adoption of agricultural machines renting but not the outcome variables, such as productivity and commercialization. The study use access to information about agricultural machine (yes=1) as a selection instrument. Thus, this variable is more likely to be correlated with the adoption of agricultural machines, but not productivity and commercialization outcome variables or correlated with the unobserved. Moreover, we also check the validity of the instrument variable using a falsification test. The test shows that the variable significantly affects the adoption decision but not our outcome variables.

The two regimes that the smallholder farmers fall in to are represented by the following two regression equations.

$$Regime\ 1: Y_I = \alpha_1 X_i + e_{1i} \quad if\ p = 1 \qquad eq(6)$$

$$Regime\ 2: Y_2 = \alpha_2 X_i + e_{2i} \quad if\ p = 0 \qquad eq(7)$$

Y1i and Y2i are the dependent outcome variables determined by the exogenous variables Xi, α1, and α2, are parameters that show the direction and strength of the relation between the outcome variable and the independent variables. e_{1i} ande_{2i} are error terms.

Finally, the error terms are assumed to have a trivariate normal distribution, with zero mean and non-singular covariance matrix expressed as:

$$cov(u_i, e_{1i}, e_{2i}) = \begin{bmatrix} \sigma^2 u & . & . \\ \sigma^2 e_1 u & \sigma^2 e_1 & . \\ \sigma^2 e_2 u & . & \sigma^2 e_2 \end{bmatrix} \qquad eq(8)$$

Where $\sigma^2 u$ variance of the error term in the selection equation, $\sigma^2 e_1$ and $\sigma^2 e_2$ are variances of the error terms in the continuous equations. $\sigma^2 e_1 u$and$\sigma^2 e_2 u$ are covariance of $u_i, and\ \ e_{1i}\ and\ e_{2i}$respectively. Since Y_{1i} and Y_{21} are not observed simultaneously a covariance of the corresponding error terms is not defined [68]. This structure of the error terms indicates that the error terms of the outcome equation and the error term of the selection equation are correlated which results in non-zero expected value of e_{1i} and e_{2i} given ui - error term of the selection equation [65]. Therefore, the expected values of the truncated error terms E(e_1 | p = 1) and E(e_2 | p = 0) are given below:

E (e_1 | p = 1) = E(e_1 | U > - Zβ)

$$+= \sigma e_1 u \frac{\varphi(\frac{Z\beta}{\sigma})}{\Phi \frac{Z\beta}{\sigma}} = \sigma e_1 u \lambda_1 \qquad eq(9)$$

E (e_2 | p = 1) = E(e_2 | U ≤ - Zβ)

$$= \sigma e_1 u \frac{-\varphi(\frac{Z\beta}{\sigma})}{1 - \Phi(\frac{Z\beta}{\sigma})} = \sigma e_2 u \lambda_2 \qquad eq(10)$$

φ and Φ are the probability density and cumulative distribution function of the standard normal distribution, respectively. The ratio of φ and Φ evaluated at Zβ is referred to as the inverse Mills ratio $\lambda 1$ and $\lambda 2$ (selectivity terms). If the estimated covariance $\sigma^2 \varepsilon 1u$ and $\sigma^2 \varepsilon 2u$ are significantly different from 0 the decision to renting agricultural machine and the outcome variable (productivity and commercialization) are correlated. This implies endogenous switching and the presence of a sample selectivity bias [69]. Further, estimations of treatment effects will be made. Average Treatment effect on the Treated and Untreated (ATT and ATU) are computed using the results for expected values of the dependent variable for users and non-users in actual and counterfactual scenarios:

$$E(Y_{1i}|p_i = 1, X_{1i}) = \alpha_1 X_{1i} + \sigma e_1 u \rho_1 \frac{\varphi(z\beta)}{\Phi(z\beta)} \qquad eq(11)$$

$$E(Y_{2i}|p_i = 0, X_{2i}) = \alpha_2 X_{2i} - \sigma e_2 u \rho_2 \frac{\varphi(z\beta)}{(1 - \Phi(z\beta)} \qquad eq(12)$$

$$E(Y_{2i}|p_i = 1, X_{1i}) = \alpha_2 X_{1i} + \sigma e_2 u \rho_2 \frac{\varphi(z\beta)}{\Phi(z\beta)} \qquad eq(13)$$

$$E(Y_{1i}|p_i = 0, X_{2i}) = \alpha_2 X_{2i} - \sigma e_1 u \rho_2 \frac{\varphi z\beta}{(1 - \Phi(z\beta)} \qquad eq(14)$$

ATT is the difference between the expected value of the outcome variable from Equations 11 and 13; this is the difference between the expected value of the dependent variable for users and if they were not used. ATU is the difference between Equations 12 and 14 which estimates the difference between the expected value of the outcome variable for non-users of agricultural machine.

PSM techniques were applied to supplement the ESR model and to assess the consistency of the results with different assumptions such as nearest neighbour matching, caliper and radius matching, and kernel and local linear matching [70]. The treatment effects will be estimated based on matching estimators selected on the common support region [71]. The average treatment effects can be estimated using the inverse propensity weighing estimates as stated in

IPSW [72] using matching techniques of Kernel Matching (KM), Nearest Neighbor Matching (NNM) and Radius Caliper Matching (RCM).

The selection of a particular approach depends on the data in question and, in particular, the degree of similarity between the treatment and control groups in terms of propensity score [73].The Average Treatment Effect of an individual i can be written as:

$$ATE = E(Y_i^1 | D_i = 1) - E(Y_i^0 \mid D_i = 0) \qquad eq(15)$$

Where ATE, Average Treatment Effect, which is the effect of treatment on the outcome variables:

$E(Yi^1 \mid Di=1)$: Average outcomes for individual, with user, if he/she would participant
$E\ (Yi^0 \mid Di=0)$: Average outcome of non-user, when he/she would non-participant

Variables Definition and Hypotheses

Adoption of agricultural machine renting: A dichotomous dependent variable measured in participation of agricultural machine renting, taking value 1 if there is participation in agricultural machine renting and 0 otherwise.

The explanatory variables:

Age of household head: Is a continuous explanatory variable that measures the number of years of the household head. According to [74] when the household age has increased, the probability of technology adoption decreased. This is as a result of limited planning horizons when households are getting old. As a result, age of the household is hypothesized to affect agricultural technology adoption (tractor, combine harvester and maize Sheller) negatively

Family size: It is the total number of family members, which is measured in terms of adult equivalent**.** Larger family sizes would be expected to decrease the probability that a farmer will use a combine harvester [38]. Consequently, family size would have a negative influence on the adoption of agricultural machines renting.

Education level of household head: Is a continuous independent variable measured in number of years attended formal education. Educated farmers would have the ability to perceive, interpret and respond to new information much faster than their counterparts without education [75] As a result, education is expected to correlate positively with the adoption of agricultural renting machines.

Total cultivated land: is a continuous variable and measured in hectares. It is frequently argued that farmers with larger farms are more likely to adopt improved technology (especially modern varieties) compared with those with small farms. According to [76] farm size of cultivated area of land has a significant influence on technology adoption decisions for wheat production. Therefore, farm size is expected to affect the adoption of agricultural machines renting positively.

Credit use: It is a dummy explanatory variable that takes the value of one if a household head has use of credit service and zero otherwise. Credits are expected to enhance farmers' financial capacity to purchase productivity enhancing. Use of credit services has a significant influence on technology adoption of wheat production [77]. So, a farmer who uses credit would have a positive influence on the adoption of agricultural machines.

Extension contact: This refers to the number of contacts per year that the household made with extension agents, and it is a continuous variable. The effort to disseminate new agricultural technologies is within the field of communication between the extension agent and the farmers at the grass root level [78]. Here, the frequency of contact between the extension agent and the farmers is hypothesized to be the potential force which accelerates the effective dissemination of adequate agricultural information to the farmers, thereby enhancing farmers' decision to adopt new crop technologies. As a result, frequent contact with extension agent would have a positive influence on the adoption of agricultural machines.

Access to information: It is measured as a dummy variable taking a value of one if the farmer had access to agricultural machine information from an extension agent, cooperative and farmers and zero otherwise. Farmers who had information from extension agents were more likely to adopt soil and water conservation practice 79]. Better agricultural machine information would

help farmers to get detailed information about benefit, renting cost, source of agricultural machine.

Off-farm activity participation: This is a dummy explanatory variable that takes a value of one if a household head participated in off-farm activities and zero otherwise. Households with higher off-farm income may reduce the time allocated to farm management and resulting in lower productivity [80]. Therefore, participation in off-farm activity would have negative influence on the adoption of agricultural machines.

Distance to the nearest market: It is measured in kilometers. Distance to the nearest market is likely to influence the adoption of agricultural machines. The closer they are to the nearest market (station), the more likely that the farmers will receive valuable information and get machines. Farmers that are located far away from the nearest markets are less likely to adopt farm inputs than farmers who live near to market due to transaction cost [54]

Distance from main road: It is a continuous explanatory variable and measured in kilometers. Distance to the main road is used as a proxy for accessibility of transportation to markets. Distance from main road far from farmers residence renting of agricultural machines more expensive since due to transport cost and time [19]. Hence, households far away from roads are expected to be less probability of machine adoption.

Distance to the development agent: It is measured in kilometers. Proximity to development agents' office has the advantage of farmers gaining awareness and expose to new ideas and information about productivity inputs and outputs [81]. Households residing in places far from development agents' office were less likely to adopt agricultural machine than those who live near to development agents' office because of higher transaction costs, or may be information gap. This might be the cost of agricultural machines directly expressed in terms of distance from the station. Thus, the adoption status of agricultural machines would be reduced.

Position of farmer in farmer's association: it is a dummy variable coded as 1 if farmers have a position in rural kebeles, or model farmers and, 0 otherwise. Farmers who have some position in rural kebeles or model farmers are more likely to be aware of new practices as they are easily

exposed to information [82]. Therefore, farmers who have a position in the association are hypothesized to have a positive relation with the adoption of agricultural machines renting.

Number of oxen: It is a continuous variable measured as the number of oxen owned by the household heads. All agricultural tasks are not performed only by agricultural machines renting; it also needs other additional oxen [83]. Thus, ownership of oxen has a positive influence on agricultural machine renting adoption.

Livestock (excluding oxen): is a continuous variable measured in terms of Tropical Livestock Units (TLU) of livestock owned by the household excluding oxen. Livestock is the farmers' important source of income, food and draft power for crop cultivation in Ethiopian agriculture. It was hypothesized that as livestock ownership increases, adoption/intensity of adoption is expected to increase because it serves as proxy for wealth status [82].

Membership to cooperative: It is a dummy variable is measured by allocating a score of 1 if a farmer is a member of an organization and 0 otherwise. Belonging to a member of a cooperative influences a farmer’s decision to adopt an improved technology. Being a member of the cooperative has a significant and positive impact on the adoption of teff row planting [84]. Therefore, farmers’ membership of a cooperative would have a positive influence on the adoption of agricultural machines.

Outcome variable

Total factor productivity: Total factor productivity index of cereal crops includes (teff, wheat and maize) produced by households in the 2020/21 production year.

Technical efficiency: Technical efficiency of cereal crops (teff, wheat and maize) in the 2020/21 production season.

Output commercialization: Cereal crop output commercialization indices include teff, wheat and maize output commercialization indicesin the 2020/21 production season.

4. RESULTS AND DISCUSSIONS

4.1. Demographic, Socio-Economic and Institutional Characteristics of households

The results statistical summary that shows the difference between machine users and non-users for demographic, socio-economic and institutional characteristics of households (Table 6). The average ages of household heads for renters and non-renters were 46.69 and 42.91 respectively. The result of the t-test showed that age of household heads had a significant mean difference between renters and non-renters at less than 1% probability level. The aged household heads were adopted agricultural machine renting better than the young aged. These maybe aged household heads have better experience and knowledge about the importance of agricultural machine renting than the young aged household heads. The overall household heads education level was an average of 3.78 years. The study result shows that the farmers who adopted agricultural machines were relatively more educated (with an average of 4.19 years and 3. 39 years for non- adopters) and the difference was statistically significant at less than 1 % probability level. Family size was another variable that had a significant mean difference between renters and non-renters at less than 5% probability level.

Moreover, the result of the t-test revealed that total land and ownership of oxen had significant mean difference between renters and non-renters at less than 1% probability level. Household heads that adopted agricultural machine renting have a comparatively larger farm size and a greater number of oxen. The study result shows that farmers who adopted agricultural machine renting reported higher frequencies of contact with extension agent than non-adopters. Non-adopter farmers were located far from the nearest markets (3.48 Km) compared to adopters (2.87Km). Distance from the nearest market had a significant mean difference between agricultural machine renting renter and non-renters at less than 1% of significance level. This shows that farmers who lived nearest to market centers adopted agricultural machine renting than those who were located far from market centers.

Table 1. Demographic, socio-economic, institutional and pilot characteristics of households by agricultural machine renting for continuous variable

Description	Machine renting N=192	Not Machine renting N=208	Total	t-test
Age of household head	46.69 (0.74)	42.91 (0.61)	44.72 (0.48)	3.98***
Education level	4.19 (0.22)	3.39 (0.21)	3.78 (0.15)	2.61***
Family size	4.86 (0.12)	4.53 (0.11)	4.69 (0.08)	-1.92**
Distance from market	2.87 (0.13)	3.48 (0.18)	3.19 (0.12)	-2.55***
Distance from road	2.34 (0.11)	2.4 (0.09)	2.38 (0.09)	0.35
Distance from DA	2.22 (0.09)	2.35 (0.09)	2.29 (0.06)	1.01
Extension visit	12.4 (0.51)	9.76 (0.35)	11.03 (0.31)	4.34***
Total land	2.41 (0.07)	1.89 (0.05)	2.14 (0.04)	5.88***
Own land holding	1.6 (0.06)	1.35 (0.05)	1.47 (0.04)	3.12***
Number of oxen	2.7 (0.07)	2.1 (0.05)	2.39 (0.04)	6.43***
Total livestock holding (TLU) excluding oxen	4.18 (0.16)	3.99 (0.15)	4 (0.1)	-1.03

The chi-square test results for categorical variables were given in Table 7. It indicated that power in social activities and use of credit had a significant mean difference between agricultural machine renting adopters and non-adopters at less than 1 % probability level. About 63.59% of non-renters farmers had power in social activity whereas 36.41% of farmers were renters. Household heads who adopted agricultural machine renting had less power to do social activity compared to non-renters. In addition, the chi square test showed that farmers who adopted agricultural machine renting were fewer users of credit. Moreover, participation in off-farm activity and access to information from extension agent about agricultural machine renting had significant differences between agricultural machine renting renters and non-renters at less than 10% probability level.

Table2. Demographic, socio-economic, institutional characteristics of households by agricultural machine renting for categorical variable

Variables	Level	No.	%	No.	%	x^2-value
Sex	Male =1	195	51.45	184	48.55	0.87
Power on social	Yes	71	36.41	124	63.59	37.0***
Member-coop	Yes	168	52.66	151	47.34	0.28
Credit use	Yes	72	41.62	101	58.38	13.16***
Off-farm parti	Yes	76	58.02	55	41.98	2.82*
Information EA	Yes	155	54.96	127	45.04	3.37*

Source: Computed from survey data (2021)

4.2. Agricultural machine renting

In the study area, tractors, combine harvestesrs and maize Sheller are used in the production process of cereal crops, either solely or simultaneously. The study showed that Table 4 approximatly 25.3% rented tractors only for ploughing services. Farmers commonly rent tractors in the study area for the first and second tillage. This is becauset the first ploughing operation is the heaviest and most challenging for oxen, whereas a tractor can plough easily and deeply. Similarly, about 22.3% of farmers rented combined harvesters only for wheat harvesting, threshing and winnowing operation and about 28.54% rented maize Sheller machines only for shelling of maize after harvesting. About 11.16% of farm households rent three types of machines (tractor, combine harvester and maize Sheller) simultaneously.

Table 4.Major agricultural machine used in the study area

Types of agricultural machines used	Freq.	Percent
Tractor	118	25.3
Combiner	104	22.3
Sheller	133	28.54
All (tractor, combiner and Sheller)	52	11.16
Tractor &combiner	17	3.65
Tractor & Sheller	20	4.3
Combiner & Sheller	22	4.72

4.3. Impacts of agricultural machine renting on productivity and commercialization

The essential tests under econometric analysis that verified the model were undertaken on hypothesized variables. The variables included in the model were tested for the problems of multicollinearity and heteroskedasaticity. The result shows that there is no problem of multicollinearity and heteroskedasaticity among the variables. Further, the model specification was carried out using the Ramsey-reset test and the results revealed that there were no omitted variables in the model (see from appendix).

Table 5 represents the first stage ESR binary probit estimation results. The probit model fits the data reasonably well [Wald Chi-squared = 137.31, p= 0.000)]. The model results reaveled that household, socioeconomic, and institutional factors had a significant impact on the decision to rent agricultural machines.

The age of the household head affected adoption of agricultural machine renting positively and significantly at 1% level. The marginal effect result indicates that aged household heads adopt more agricultural machines than younger households because, as the age of households increases, working power decreases. This finding is similar to [13]. Agricultural machine renting increased with the level of education of the household heads. Household heads who attened better education level have a good awareness about agricultural machines. Educated household heads are more likely to use agricultural machines [10,39]. The total land cultivated significantly and positively affected the adoption of agricultural machines. Farmers who owned large amount of cultivated land were more likely to adopt agricultural machines than small land-holding farmers. [63] found farm size was a positive influence on the adoption of agricultural technology packaging. Ownership of large amounts of cultivated land often required more labor and time to perform agricultural operations.

Frequency of extension contact influenced the adoption of agricultural machines significantly and positively at the 1% level. This implies that farmers who are frequently in contact with extension agents are more likely to adopt agricultural machines than are those in lower contact with extension agents. This is because, development agents provide advice on agricultural machine utilization and benefits to farmers. The extension provides in-depth information, training and consultancy services on the source, application and relevance of agricultural

technologies for farmers. Similarly, farmers with information about agricultural machines from government extension agents are more likely to adopt agricultural machines. This is because the provision of information helps farmers to become aware of the benefits and costs of machines. The use of credit negatively and significantly affected the probability of farmers using agricultural machines to produce cereal crops at 1%. The result indicated that farmers who used credit services decreased the probability of agricultural machines renting. This may be because households used credit for consumption, fattening livestock and home construction.

Distance from the market and the main road negatively and significantly affected the adoption of agricultural machines by 10% and 5%, a significant level. Proximity to the market and main roads are important factor in adopting agricultural machines because better access to markets and roads is an indicator of access to information and infrastructure. Thus, farmers who live near the market and main road are more likely to rent agricultural machines and other important inputs at a lower cost than those who live far away from markets and main roads. Similarly, [85] the adoption decision of smallholders affected by distance from the marketplace due to cost of production. The number of oxen affects the adoption of agricultural machines positively and significantly at 1%. This implies an increase in the number of oxen and the probability of adopting agricultural machines increases. This means that households with many oxen perform agricultural activities like the second plow, third plow, sowing and threshing more easily than thos fewer oxen.

.

Table 5. Decision of adopting agricultural machines renting: Probit model

Variables	Coeff	Std.Err	Marginal Effect
Age of household	0.03***	0.009	0.012
Education level of household	0.09***	0.026	0.036
Adult equivalent	-0.11	0.059	-0.045
Poweron social activity	-0.49***	0.149	-0.196
Market distance	-0.07*	0.043	-0.031
Road distance	0.14**	0.073	0.058
Distance from office	0.1271	0.086	-0.050
Extension visit	0.0376***	0.012	0.015
Member of cooperative	0.2692	0.185	0.107
Credit use	-0.4276***	0.146	-0.170

Total land	0.3655***	0.040	0.145
TLU	-0.0372	0.041	-0.014
Number of oxen	0.3934***	0.110	0.156
Off farm activities	-0.0666	0.158	-0.026
Information for mechanization	0.3425**	0.165	0.136
-Cons-	-2.1487***	0.705	

Source: Computed from survey data (2021)

The results of the second stage ESR and,the estimated coefficients of total factor productivity, technical efficiency and commercialization index are presented in the appendix

Table 6 shows the results ATT and ATU for the key outcome variables related to agricultural machine renting. In this analysis the main outcome vaiables are TFP, TE and COIM index. Before, we analyzed the result of ESR model to check the validity of instrumental variables using a falsification test. The falsification test shows the instrumental variable significantly affects the adoption of agricultural machines but not TFP, TE and COIM index. Instrument variables are jointly statistically significant in the adoption equation [$\chi 2$ = 4.22 (p = 0.04)] but not outcome functions. The instrumental variable did not affect the TFP [F=0.10(p= 0.7497)] technical efficiency [F=0.45 (p=0.5038)], and commercialization index [F= 1.92 (p=0.1668)].

The adoption of agricultural machines was renting increases total productivity, technical efficiency and commercialization level of farm households. For farmers who adopted agricultural machine renting but theoretically would have not adopted, then their TFP decreased by 0.573 (57.3%). This means, the ATT of total factor productivity of cereal crops would decrease by 57.3% as a result of did not use agricultural machines renting. Likewise, if non-users of agricultural machine adopted agricultural machine renting, then their TFP would have significantly increased by 0.419(41.9%). For farmers who adopted agricultural machines but theoretically would not have adopted them, then their TE decreased by 0.104 (10.4). This means, the ATT of TE on cereal crops would decrease by 10.4% due to do not adopting agricultural machines. Similarly, if non-users of agricultural machine adopted agricultural machines, then their TE would have significantly increased by 0.064(6.4%). In addition, the results of the commercialization index of cereal crops for adopters would drop by 0. 12(12%) if the adopters

were not adopted. In the same way, if the non-users of agricultural machine had adopted agricultural machine renting, their COIM would have increased by 0.098 (9.8%).

Consequently, ESR shows that in Ethiopia the adoption of agricultural machine renting would have increased productivity and commercialization of cereal crops. This result resembles to [3] the adoption of agricultural technology has a positive effect on crop productivity including wheat. The adoption of agricultural technologies has also a positive and significant impact on productivity of teff [84].

Table. 6 Endogenous switching regression model result (average treatment effects)

Outcome variables	Mean farm household type and treatment effect	Decision stage		Treatment effect
		To Adopt	Not to Non-adopt	
Productivity	ATT	3.1	2.52	0.573***
	ATU	3.13	2.7	0.422***
TE	ATT	0.845	0.741	0.104***
	ATU	0.837	0.772	0.064***
COIM	ATT	0.572	0.452	0.120***
	ATU	0.544	0.444	0.099***

Note. ATT = Adoption effect for adopters, ATU = Adoption effect for non-adopters, and *** $p < .01$.

Popensity Score Matching Estimation Results

In addition to the ESR model, this study uses the PSM technique to check the robustness of the results obtained from the ESR model. Propensity scores (the probability of adoption in agricultural machine) are estimated using a probit model. Figure2 shows the distribution of adopter and non-adopter households with respect to estimated propensity scores. The figure illustrates the estimated propensity distribution for treatment and control households. The upper half of the graph refers to the propensity score distribution of treatment groups, while the bottom half shows the control groups. The y-axis refers to the densities of estimated propensity scores.

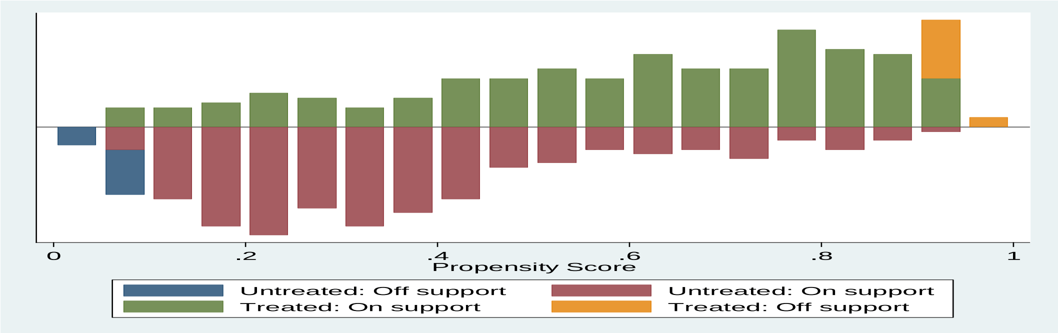

Figure 2: Distribution of estimated propensity distribution for treatment and control groups and common support area

There should be a common support condition that should be applied to how the propensity score is distributed between adopters of agricultural machine renting and those who do not. The estimated propensity scores varied between 0.0826423 and 0.9633814 (mean =0. 6249002) and 0. 0347458 and 0.92732443 (mean, =0.3462459) in the treatment and control groups, respectively. The common support lies between 0.0826423 and 0.92732443. Table 7 presents the balancing tests for each matching algorithm before and after matching. The results show that the mean standardized bias is reduced after matching from 10.3 to 5.3 percent) compared to before matching (38percent). Similarly, Pseudo-R^2 declined substantially, from 22.4 percent to 1 percent. The p-values from the likelihood ratio tests showed that all of the covariates were statistically significant at a p-value of less than 1 percent prior to matching, but insignificant post-match. These p-values clearly demonstrate that the matching process balance the observed characteristics between treated and control after matching.

Table 7.Covariates balancing tests before and after matching

Matching algorism	Pesido-R-square		LRx^2P −value		Mean standard bias	
	Before	After	Before	After	Before	After
NNM1	0.224	0.010	123.87 (0.00)	4.99 (0.986)	38	5.3
NNM2	0.224	0.014	123.87 (0.00)	6.71 (0.278)	38.	6.9
KBM3	0.224	0.010	123.87 (0.00)	4.83 (0.729)	38	6.6
KBM4	0.224	0.011	123.87 (0.00)	5.36 (0.980)	38.	6.5
RCM5	0.224	0.030	123.87 (0.00)	8.55 (0.881)	38	7.8
RCM6	0.224	0.030	123.87 (0.00)	10.22 (0.746)	38	10.3

NNM1 = One nearest neighbor matching and common support

NNM2 = Two nearest neighbor matching and common support

KBM3 = Kernel with band width 0.05 and common support

KBM4 = Kernel with band width 0.1 and common support

RCM4 = Radius Caliper 0.05 matching

RCM5= Radius Caliper 0.1 matching

Table 8 shows the result of PSM, using three different matching algorithm techniques (nearest neighbor matching (NNM), Kernel based matching (KBM), and Radius matching methods). The result reveals that, as in the ESR analysis, the adoption of agricultural machine renting resulted in an increase in TFP, TE and COIM. The PSM result revealed that increased adoption of agricultural machines renting significantly increases total factor productivity of crops in the range of 0.41 to 0.52 (41 to 52 percent). Similarly, the adoption of agricultural machines increases technical efficiency from 0.077 to 0.104 (7.7% -10.4%). Moreover, on average, the adoption of agricultural machines increased the household's commercialization level in the range from 0.102 to 0.116 (10 to 11.6 percent). Therefore, it can be concluded that, apart from the minor differences in magnitude between the PSM and ESR estimates, the results of ESR and PSM indicate that the adoption of agricultural machines had positive impacts on TFP, TE and

COIM levels. This finding is similar to [79] the result from PSM and ESR estimates of soil and water conservation practice on food insecurity and net crop value.

Table 8.Average treatment effects: propensity score matching

Outcome variable	Matching algorism	Mean of outcome variables based on matched observation		ATE	ATT	SE
		Adopter	Non-adopter			
COMI						
	NNM_1	0.573	0.459	0.1105	0.114	0.015
	NNM_2	0.573	0.459	0.105	0.113	0.016
	KBM_3	0.573	0.46	0.105	0.113	0.016
	KBM_4	0.573	0.459	0.105	0.113	0.016
	RCM_5	0.555	0.456	0.098	0.100	0.013
	RCM_6	0.567	0.451	0.108	0.116	0.011
	NNM_1	3.10	2.57	0.46	0.524	0.104
TFP	NNM_2	3.10	2.6	0.44	0.49	0.102
	KBM_3	3.10	2.58	0.44	0.511	0.093
	KBM_4	310	2.58	0.45	0.513	0.090
	RCM_5	3.12	2.64	0.46	0.47	0.080
	RCM_6	3.11	2.68	0.39	0.411	0.066
	NNM_1	0.847	0.753	0.089	0.093	0.020
	NNM_2	0.847	0.745	0.089	0.102	0.017
TE	KBM_3	0.847	0.749	0.085	0.098	0.015
	KBM_4	0.847	0.751	0.84	0.096	0.015
	RCM_5	0.847	0.773	0.073	0.077	0.010
	RCM_6	0.847	0.774	0.070	0.077	0.009

5. SUMMARY

The agricultural sector in Ethiopia is the main source of economic development, and its growth originates from smallholder farmers. Even though smallolder agricultural production and productivity still low. Thus, this study analyzed the of impact agricultural mahines on the productivity and commercialization of cereal crops in West Gojjam, Ethiopia. A series of formal and informal surveys were carried out during the 2020/2021 crop season on 400 sample farm household heads that were randomly drawn from six kebeles in three woredas through a multi-stage sampling procedure. Secondary data were collected from published and unpublished sources to support the primary data.

Descriptive statistics and econometric models were used to analyze the data. The descriptive statistics include the mean, percentage, t-test and chi-squre tests. Econometric models, including endogenous switch regression (ESR) and propensity score matching (PSM) were employed.

The descriptive statistics showed that approximately 48% sample of households were renting agricultural machines to perform different agricultural operations. The study also investigates the total factor productivity and technical efficiency of cereal crops by using the total factor productivity index and stochastic frontier function. The results showed that the total factor index of the cereal crop was 2.89 and the stochastic frontier model showed that technical efficiency of cereal crop producers was 80.6%. The impact of agricultural machine renting on productivity and commercialization was estimated using endogenous switch regression and propensity score matching models. In these models, 192 machine users and 208 non-users of agricultural machines were used. The results of the first ESR model showed that age, education level, ownership of oxen, total cultivated land, extension visit, access to information, family size, the position a farmer, and distance to the nearest market significantly affected adoption of agricultural machine renting. The results of the endogenous switch regression and propensity score matching model indicate that the adoption of agricultural machines has positive and significant impacts on total factor productivity, technical efficiency and output commercialization index.

5.1. Policy implication

The adoption of agricultural machines has a positive and significant impact on the productivity and commercialization of cereal crops. This result indicates that agricultural mechanization enhances productivity and commercialization of cereal crops in the study area. When agricultural mechanization increases productivity and commercialization, we should examine what factors determine agricultural machine renting. Based on the result of study determined by demographic, socio-economic and institustional factors. Therefore, Therefore, governmental and non-governmental stakeholders give more emphasis on:

- formulating appropriate policies to provide adequate and effective basic educational opportunities for rural farming households.
- deliver selective and appropriate agricultural machines to smallholder farmers
- creating better extension services and offering practical training or demonstration on the utilization of agricultural machines for plough, harvesting, threshing and shelling of crops.
- strengthening the existing rural-urban market centers, rural-urban roads and other infrastructure development activities in the study area.

Finally, the government also should formulate a supporting policy for local importers of agricultural implements and strengthen their capacity.

Authors contributions:
Conceptualization, Selam Tilahun, Berhanu Kuma and Amsalu Bedemo
Methdolgy, Selam Tilahun
Validation, Berhanu Kuma and Amsalu Bedemo
Formal analysis, Selam Tilahun
Investigation, Selam Tilahun
Data curation, Berhanu Kuma and Amsalu Bedemo
Writing original draft,Selam Tilahun
Writing review and editing, Selam Tilahun
Visualization, Berhanu Kuma and Amsalu Bedemo
Supervision, Berhanu Kuma and Amsalu Bedemo
Availability of supporting data: the data presented in this study are available from the author upon request

Author Declaration: The authors declare no conflict of interest.

6. REFERENCE

1. World Bank Indicators Washington, DC, 2020. Author

2. CSA (Central Statistical Agency), Agricultural sample survey report on Area and production of major Crops (private peasant holdings, meher season 2016 / 2017; Addis Ababa Ethiopia, the FDRE statistical bulletin, Volume I.

3. Fisseha Z , Abrham S, and Dawit, A . Cereal production practices and technical efficiency among farm households in major “teff” growing mixed farming areasof Ethiopia:CogentEconomics&Finance, 2022;10:1, doi: 10.1080/23322039.2021.2012986

4. Doss C. R, W. M. Mwangi, H. Verkuijl, and H, De Groote. Adoption of Maize and Wheat Technologies in Eastern Africa: A Synthesis of the Findings of 22 Case Studies. International Maize and Wheat Improvement Center (CIMMYT) Economics Working Paper Mexico City,2020, http://repository.cimmyt.org/handle/10883/1037

5. Shita A, Kumar N, and Singh S. Agricultural Technology Adoption and its Determinants in Ethiopia: A Reviewed Paper. Asia Pacific Journal of Research,I(LVV) 2018, 99–104

6. Macauley H. Feeding Africa: Cereal Crops: Rice, Maize, Millet, Sorghum, Wheat. 2015; Vol. 86, AbdouDiouf International Conference Centre, Senegal, 74.

7. CIMMYT . Farm mechanization and conservation Agriculture for sustainable intensificatio(FACASI) project.Market analysis for small mechanization Ethiopia,2014 .

8. Dagninet A, and Wolelaw E. Agricultural Mechanization: Assessment of Mechanization Impact Experiences on the Rural Population and the Implications for Ethiopian Smallholders. Engineering and Applied Sciences, 2016; 1(2): 39-48.

9. Houmy K. M, F. Rojas, and C. Side,. Agri-hire in sub-Saharan Africa Business models or investing in sustainable mechanization Rome, https://doi.org/,2021,10.4060/cb5071en

10. Berhane G, Kalle H, and Bart M. Agricultural Mechanization in Ethiopia: Evidence from the 2015 Feed the Future survey, International Food Policy Research Institute.

11. Feder G, Just R. E, and Zilberman D. Adoption of agricultural innovations in developing countries: A survey. Economic development and cultural change, 1998;.33(2) :255-298

12. Adu-B F, Daum T,and Birner R. Can small farms benefit from big companies’ initiatives to promote mechanization in Africa? A case study from Zambia. Food policy, 2019; 84, 133-145.

13. Kirui O. The Agricultural mechanization in Africa: micro-level analysis of state drivers and effects. ZEF-Discussion Papers on Development Policy, 2019 ; (272).

14. Yukichi Y, Mano, Yukichi Y, Takahashi K,Otsuka K. Contract Farming, Farm Mechanization, and Agricultural Intensification: the Case of Rice Farming in Cote d'Ivoire. Working Papers, 2017; vol. 157 JICA Research Institute.

15. Houssou N, and Chapoto A. Adoption of farm mechanization, cropland expansion, and intensification in Ghana, 2015. (No. 1008-2016-80214).

16. Kahan D, Bymolt R, and Zaal F. Thinking outside the plot: insights on small-scale mechanisation from case studies in east Africa. J. Dev. Stud, 2017;1–16.

17. Cossar F. Impact of mechanization on smallholder agricultural production: evidence from Ghana, 2019 (No. 2229-2019-1910).

18. Daum T, and Birner R. Agricultural mechanization in Africa: Myths, realities and an emerging research agenda. Global Food Security, 2020; 26, 100393. https://doi.org/10.1016/j.gfs.2020.100393

19. Berhane G, Dereje M, Minten B, and Tamru S. The rapid – but from a low base – uptake of agricultural mechanization in Ethiopia : Patterns, implications and challenges. International Food Policy Research Institute (IFPRI). ESSPWorkingPaper,2017; 105

20. Diao X, Cossar F, Houssou N, and Kolavalli S. Mechanization in Ghana: Emerging demand, and the search for alternative supply models. Food Policy, 2014.; 48, 168–181. https://doi.org/10.1016/j.foodpol.2014.05.013

21. Takeshima H. Market imperfections for tractor service provision in Nigeria: International perspectives and empirical evidence, 2016; 32. Intl Food Policy Res Inst.

22. NPC. 2016. Growth and Transformation Plan II (GTP II) (2015/16-2019/20), Volume I: Main Text

23 Tadesse W, Bishaw Z, and Assefa S. Wheat production and breeding in Sub-Saharan Africa: Challenges and opportunities in the face of climate change. *International Journal of Climate Change Strategies and Management*, 2019, 11(5), 696–715. https://doi.org/ 10.1108/IJCCSM-02-2018-0015

24 Belete A. Analysis of technical efficiency in maize production in Guji Zone: stochastic frontier model. Agric & Food Secure, https://doi.org/,2020,10.1186/s40066-020-00270-w

25 Yohannes M. T, Paulos A, and Dawit T. S. Factors affecting farmers' hiring decisions on agricultural mechanization services: A case study in Ethiopia, Cogent Economics & Finance, 2023, 11:2, 2225328, DOI: 10.1080/23322039.2023.2225328

26. Agricultural Transformation Agency. Annual report of 2013/14 Addis Ababa

27. FAO and AUC. Sustainable Agricultural Mechanization: A Framework for Africa. Addis Ababa, 2018; 127pp. Licence: CC BY-NC-SA 3.0 IGO

28 Rosegrant M, W, Koo, J,Cenacchi N, Ringler C, Robertson R, Fisher M, Cox C, Garrett K, Perez N. D, and Sabbagh P. Food Security in a World of Natural Resource Scarcity the Role of Agricultural Technologies. Monographs of the Society for Research in Child Development, 2014; 79 (1)

29. Yared D. Agricultural mechanization, enabling policy, machinery suppliers, hiring services, heterogeneity, hiring cost doi: https://doi.org/,2021,10.21203/rs.3.rs-478878/v1

30. CSA (Central Statistical Agency). Agricultural sample survey report on Area and production of major Crops (private peasant holdings, meher season 2019 / 2020; Addis Ababa Ethiopia, the FDRE statistical bulletin, Volume I

31. Bantelay T, Tsehay D, and Nigist k . An integrated approach to solve small farm holder's mechanization barriers in Ethiopia. Computer science ,Social Telecomnication Enginering, .https:doi.org/,2020,10.1007/978-3030-43690-2_4.

32. Wudu B. Deteraminants of adoption of improved wheat technology: in case of Gozzamen district, east Gojjam in Amhara regional state, Ethiopia (thesis, St. Mary's University) ,2017.

33. Gebru A. The Determinants of Modern Agricultural Inputs Adoption and Their Productivity in Ethiopia (The Case of Amhara and Tigray Regions) (thesis, Addis Ababa University), 2016.

34. Worku A. Factors affecting diffusion and adoption of agricultural innovations among farmers in Ethiopia case study of Ormia regional state WestsernShewa. International Journal of Agricultural Extension, 2019;7(2):137–147. https://doi.org/10.33687/ijae.007.02.2648

35 Workineh M, Lakew T, and Haile K. E. Agricultural technology adoption and its impact on smallholder farmers welfare in Ethiopia. African Journal of Agricultural Research, 2020; 15(3): 431–445. https://doi.org/10.5897/AJAR2019.14302

36. Massresha E, Lema Z, Neway, and Degua A. Perception and determinants of agricultural technology adoption in north shoa zone, Amhara regional state, Ethiopia. Cogent Economics & Finance, 2021; 9(1), 1956774.https://doi.org/10.1080/23322039.2021.1956774

37 Tamirat N, and Abafita J. Adoption of row planting technology and household welfare in southern Ethiopia, in case of wheat grower farmers in Duna district, Ethiopia. Asia-Pacific Journal of Science and Technology, 2021; 26(2).

38. Hassena M, Regassa E, Mwangi H, and Verkuijl H. A Comparative Assessment of Combine Harvesting Vis-à-vis Conventional Harvesting and Threshing in Arsi Region, Ethiopia.

Mexico, D.F. (CIMMYT) and Ethiopia Agricultural Research Organization(EARO) , 2000.

39. Tamrat G, Mengistu K, Arega S, and Getachew L. Determinants of farm mechanization in centraland southeast oromia region, Ethiopia,2023.https://doi.org/10.1016/j.heliyon.2023.e18390

40. Boserup E. The Conditions of Agricultural Growth.The economics of agrarian change under population pressure. New Brunswick, USA: Transaction Publishers, 1965.

41. Pingali P, Bigot Y, and Binswanger H.P. Agricultural mechanization and the evolution of farming systems in Sub-Saharan Africa. Am. J. Agric. Econ 1987, 70 (2), 498.

42. Binswanger H, and Ruttan V. Induced Innovation. Baltimore and London: The Johns Hopkins University Press,1978.

43. Apiors E, Kuwornu J, and Kwadzo, G. Effect of mechanization use intensity on the productivity of rice farms in southern Ghana. Acta agriculture Slovenica, 2016,107(2): 439.

44. Din R,U, and Khattak N,U. Impacts of farm mechanization om wheat and maize productivity in Peshawar valley,Sarhad Journal of Agriculture, 2018,34(3)

45. Peng J, Zhao Z, and Liu D. Impact of agricultural mechanization on agricultural production, income, and mechanism: evidence from hubei province, china. front. environ. sci. 10:838686. doi: 10.3389/fenvs.2022.838686

46 .West Gojam West Gojjam Zone of Agriculture. West Gojjam Zone Bereau of Agriculture and Rural Development, Amhara Region, Ethiopia,2021.

47. Cochran, W.G. Sampling Technique. 2nd Edition, John Wiley and Sons Inc., 1963, New York.

48. Bamidele F.S, Babatunde R.O, and Rasheed A. Productivity analysis of cassava-basede production systems in the Guinea Savannah: Case study of Kwara State, Nigeria. American-Eurasian Journal of Scientific Research, 2008; 3 (1): 33-39.

49. Key N. and Mcbride W. Production contracts and productivity in the US hog sector. American Journal of Agricultural Economics, 2003; 85 (1): 121-133

50. Ukoha O.O, Okoye B.C and Emetu J. Analysis of the Determinants of Total Factor Productivity among Small-Holder Cassava Farmers in Ohafia L.G.A of Abia State, 2010.

51. Govereh J, Jayne T, and Nyoro J. Smallholder commercialization, interlinked markets and food crop productivity: Cross country evidence in eastern and southern Africa, Michigan State University, 1999.

52. Pingai P. From subsistence to commercial production systems: The transformation of Asian agriculture. American journal of agricultural economics, 1997,79: 628-634

53. Osmani A, and Hossain E. Market participation decision of smallholder farmers and it's in Bangladesh. Journal of Economics of Agriculture 1/2015UDC: 631.1.0173:339.3

54. Alelign A. Crop productivity, efficiency and commercialization of smallholder farmers in the Highlands of Eastern Ethiopia in partial fulfillment of the requirements for the degree of doctor of philosophy in agricultural economics Haramaya University, Ethiopia,2017.

55 Addisu G, Degye G, and Adam B . Commercialization of teff: The Case of Smallholder Farmers in Dendi District of Oromia, Central Ethiopia. International Journal of African and Asian Studies, 2019, 56.

56. Birara E, Mezegebu A, Fenta A, and Zemen A. Determinants of Wheat Commercialization among Smallholder Farmers in Debre Elias Woreda, Ethiopia: Advance in agriculture, https://doi.org/,2020,10.1155/2020/2195823

57. Von Braun J, and Kennedy E. Agricultural Commercialization, Economic Development and Nutrition.The Johns Hopkins University Press, Baltimore and London,1994.

58. Strasberg P, Jayne T, Yamano T, Nyoro J, Karanja D, and Strau*--ss, J. Effects of agricultural commercialization on food crop input use and productivity in Kenya: Office of sustainable development; policy synthesis for USAID-Africa Bureau, 1999.

59. Berhanu G, and Moti J. Commercialization of smallholders: Does market orientation translate into market participation? Improving Productivity and Market Success (IPMS) of Ethiopian farmer's project, working paper, 2010; 22: 30 p.

60. Coelli T.J, and G.E. Battesse. A Model of technical efficiency effects in stochastic frontier function for panel data. Journal of Empirical Economics, 1995; 20: 325-332.

61. Meeusen W, and van den Broeck J. Efficiency estimation from Cobb-Douglas production functions with composed error. International Economic Review, 1977; 18: 435-444.

62. KabungaN. S, Dubois T, and Qaim M. Yield effects of tissue culture bananas in Kenya: Accounting for selection Bias and the role of complementary inputs. Journal ofAgricultural Economics, 2012; 63:444–464. https://doi.org/10.1111/j.1477-9552.2012.00337.

63. Shiferaw B, Kassie M, Jaleta M, and Yirga C. Adoption of improved wheat varieties and impacts on household food security in Ethiopia. Food Policy, 2014; 44:272–284. https://doi.org/10.1016/j.foodpol.2013.09.012

64. Ahmed M, Kassaun G, Amreo, T, and Eeden A. The impact of improved maize varieties on farm productivity and wellbeing: Evidence from the east Hararghe zone of Ethiopia. Development Studies Research, 2017; 4, 9–21.

65. Abdulai, A, and Huffman W. The adoption and impact of soil and water conservation technology: An endogenous switching regression application. Land Economics, 90, 26–43. https://doi.org/10.3368/le.90.1.26, 2014.

66.Jaleta M, Kassie M, Marenya P, Yirga C, and Erenstein O. Impact of improved maize adoption on household food security of maize producing smallholder farmers in Ethiopia. Food Sec, 10: 81–93. https://doi.org/10.1007/s12571-017-0759-y, 2018.

67. Khonje M, Manda J, Alene A. D, & Kassie M. Analysis of Adoption and Impacts of Improved Maize Varieties in Eastern Zambia. World Development, 2015; 66: 695- 706.

68. Maddala G.S. Limited dependent and qualitative variables in econometrics. Cambridge, U.K.: Cambridge University Press, 1983.

69. Maddala G.S, and Nelson F.D. Switching regression models with exogenous and endogenous switching. Proceeding of the American Statistical Association (Business andEconomics Section), 1975; pp. 423-426.

70. Caliendo, M, and Kopeinig S. Some practical guidance for the implementation of propensity score matching, Discussion paper No. 1588, University of Cologne,2008.

71. Owusu V, and Awudu A. Nonfarm Employment and Poverty Reduction in Rural Ghana: APropensity-Score Matching Analysis, 2009. DOI 10.22004/ag.econ.51363

72. Nguezet P, Diagne A, Okoruwa V, and Ojohomon V. Impact of improved rice technology on income and poverty among rice farming household in Nigeria; contributed paper prepared for the 25th conference of the Centre for the Studies of African Economics, 2011.

73 Dehejia R, and Wahba S. Propensity score matching methods for non -experimental causalstudies. The Review of Economics and Statistics, 2002; 84(1):151–161.

74. Nicholson CF, Thornton PK, Mohammed L, Muinga RW, Mwamachi DM, Elbasha EH, Staal SJ, and Thorpe W .Smallholder dairy technology in coastal Kenya: An adoption and impact, 1999.

75. Getahun D, Mwangi H, Verkuijl, and Abdishekur W. Assessment of the Adoption of Seed and Fertilizer Packages and the Role of Credit in Smallholder Maize Production, 2000.

76. Mulugeta T, and Hundie B. Impacts of Adoption of Improved Wheat Technologies on Households‟ Food Consumption in Southeastern Ethiopia. Selected Poster prepared for presentation at the International Association of Agricultural Economists (IAAE) Triennial Conference, Foz do Iguaçu, Brazil, 18-24 August, 2012

77. Tolessa Alemu. Adoption and impact of improved agricultural practices and wheat production efficiency of smallholders in arsi zone of Ethiopia. In partial fulfillment of the requirements for the degree of doctor of philosophy in agricultural economics,2014.

78. Grimachew S. Determinants of Adoption of soil and water conservation practices in the environments of Semin Mountains: National Parks Eth. An M.Sc. Thesis Submitted to School of Graduate Studies of Haramaya University, 2005.

79. Million S. Determinants for adoption of physical soil and water conservation measures by smallholder farmers in Ethiopia Doctoral Dissertation, Sokoine University of Agriculture, Morogoro, Tanzania 80.Ana 2009

81. Assefa T, Jema H, Lemma Z, and Dawit A. Commercialization of small holder pulse producer east Gojjam, Ethiopia. Sustainable Agriculture Researc, 20218(4)

82. Habtemariam A. The comparative Influence of Intervening variable in the adoption of Maize and Dairy Farmers in Shashemene and Debrezieit, Ethiopia.PhD Thesis, University of Pretoria, 2004.

83. Astewel T, Yihenew Se. Socio-economic analysis of conditions for adoption of tractor hiring services among smallholder farmers, North-westernEthiopia. Cogent Food and Agriculture https://doi.org/, 2018,10.1080/23311932.2018.1453978

84. Etsehiwot S. The Determinants of Agricultural Technology Adoption and Its' Impact on teff productivitye in the Case of Amhara and Oromia National Regional State. Degree of Masters of Science in Economics A.A, Ethiopia, 2018.

85. Mesele Z, Abebaw H, and Getamesay B. Determinants of multiple agricultural technology adoption: evidence from rural Amhara region, Ethiopia, Cogent Economics & Finance, 2022; 10:1, DOI: 10.1080/23322039.2022.2058189.

Click here to access/download
Supporting Information
Appendix.docx

Click here to access/download
Supporting Information
selam.dta

Click here to access/download
Supporting Information
ethical approval.docx

Revised Manuscript with Track Changes

Impacts of Agricultural Machine Renting on Cereal Crop Productivity and Commercialization in West Gojjam Zone, Ethiopia.

SelamTilahun[a] ,BerhanuKuma[b] and AmsaluBedemo[c]

a. Email:selam4998@gmail.com,Department of Agricultural Economics, Wolaita Sodo University
b. Email:berhanukuma@yahoo.com, Department of Agricultural Economics, Wolaita Sodo, University
c. Email:amsalu72@gmail.com, Department of Policy Studies, Ethiopian Civil Service University

ABSTRACT

Agricultural mechanization plays an essential role to increase production, productivity and commercialization of cereal crops. Despite the Ethiopian's government has made substantial efforts to increase production, productivity and commercialization of cereal crops by the introduction of selective and adaptable agricultural machines to smallholder farmers has remained low. This stu[illegible]herefore, investigated the impacts of agricultural machine renting on cereal crop productivity and commercialization. The study used cross-sectional data collected from 192 agricultural machine users and 208 non-users from West Gojjam zone, Ethiopia. The data were analyzed using descriptive statistics and econometric models. The econometric model includes Trans log production function, Endogenous switch regression and propensity score matching. The result of descriptive statistics showed that the total factor productivity index of the cereal crop was 2.89, and the mean commercialization index for the sample households was 0.506. The estimation of results of Trans log production function model showed an average level of Technical Efficiency of 80.6%. The result of first stage Endogenous switch regression model showed that age, education level, ownership of oxen, total cultivated land, extension visit, access to information, family size, the position a farmer, and distance to the nearest market significantly affected adoption of agricultural machine renting. The result of endogenous switching regression and propensity score matching models showed that agricultural machine renting had a positive and significant impact on the selected outcome variables, total factor productivity, technical efficiency and output commercialization index. Based on the findings, the study suggests that the government and stakeholders should focus on strengthening the provision of education, development of infrastructures, extension service, to promote agricultural machine renting service, and enhance cereal crops productivity and commercialization.

Keywords: Agricultural machine, Productivity, Commercialization, Endogenous switch regression, Impact

1. INTRODUCTION

Sub-Saharan Africa (SSA) is one of the regions in the world that is mainly characterized by smallholder farm households, where their livelihood depends primarily on agriculture. Similarly, Ethiopia's economy mainly depends on agriculture, which is the main source of income for 84% of the population, and it accounts about 33.8% of the country's Gross Domestic Product (GDP) and 70% of export earnings [1]. Cereal cultivation and marketing is the primary source of income for millions of subsistence farmers in the country. Cereals are accounts for 60 percent of rural employment in Ethiopia, 80% of cultivated land in the country, over 40% of food expenditure for the typical household, and over 60% of total calorie intake [2]. Cereals are the main food crop in terms of area coverage and production.

Cereal crop production and marketing have shown remarkable progress in Ethiopia, yet the domestic price for cereals is growing from time to time due to the rapid increase in domestic demand, rapid population growth, income, and urbanization [3]. Given the importance of cereal crops in national food security endeavors, smallholder farmers have used traditional methods of production with limited mechanization. Thus low productivity and, limited adoption of agricultural technologies including mechanizations and subsistence-based smallholder farming are characteristics of the sector [4,5]. Specifically, a low level of mechanization in agriculture continues to be the main obstacle to advancing cereal production, which, in turn, results in a high cost of production [6]. As a result, agricultural mechanization is among the mechanisms that increase production and productivity and reduce food insecurity [7].In the same way, according to [8] and [9] the utilization of appropriate agricultural mechanization technologies helps to increase the productivity and commercialization of cereal crops.

.

However, there is a public debates about the benefit of agricultural mechanization. According to the International Labor Organization (ILO, 1973) "mechanization leads to unemployment and is linked to economies of scale, meaning that it is more likely to be bought by larger farms [10,11]. In Africafew studies have conducted on impacts of mechanization on employment, and those that have mostly reported mixed effects [12,13,14,15] and positive effect [16,17]. According to [18] the impacts of mechanization on employment depend on location-specific factors such as

wages, land availability, and type of mechanization. On the other hand, smallholder farmers can take advantage of mechanization by renting markets and cooperative exchange [19,20,21].

Developing and intensifying the cereal crops production has been the core of Ethiopia's agricultural development strategies and investments in the last 20 years to address the problem of low agricultural production and productivity [3]-Even though, still now, most of the interventions focused on the use of modern agronomic practice to improve agricultural productivity and production [22]. As a result, the problem has been continued with scanty achievement [8]. Many scholars state that the problem of low productivity is mainly related to low technical efficiency [23,24, 3]. Thus, high productivity can be achieved by using appropriate agricultural machines with agronomic practice in the right manner [25]-Consequently, using appropriate and selective agricultural machines is the best strategy to increase production and productivity of crops, commercialization, food security [26, 9].

Ethiopia's agriculture sector has depended on inexpensive and surplus labour for a long time. However, the condition is currently changing owning to the rapid migration of young people from rural to urban areas and the availability of universal access to education for all school-aged children [27,28]. This has led to a shortage of farm labor and pressure on the remaining household members. Additionally, the cost of oxen and lack of grazing land have become a major concern for smallholder farmers [19]. Consecquently, the use of machinery rental services has become a viable option for smallholder farmers. Recently, renting agricultural machines by smallholder farmers has been used in Ethiopia. Similarly, the Amhara region is one of the cereal crop growing areas and agriculture is the primary economic sector, but only 4.35% of agricultural activities oprate mechanically, which is far below the national average [29]. Likewise, West Gojjam zone is the major cereal crop production area across the nation [30]. However, utilization of agricultural machine with in this area remains minimal with farming practice relying on age-old and tradtional implements. Moreover, in recent times there has been increase in the population and a significant rise in average labor wage. This has lead to an increase the demand for agricultural machine renting among in small farmers in West Gojjam zone [31].

A number of studies have been conducted on the factors affecting the adoption of agricultural technology in Ethiopia; for example[32,33,34,35,36,37]. Only a few studies have been conducted on what may be the determinant of the adoption of the agricultural mechanization services with no specific studies on the agricultural machine renting services [38,19,39]. These previous studies applied logit, linear probability and tobit models to examine factors affecting agricultural mechanization. An important innovation in this paper was examining the impacts of agricultural machine renting on the productivity and commercialization of cereal crops. Hence there is no study on the impact of agricultural mechanization on productivity and commercialization. ~~As~~ As result, this study provides information on smallholder farmers' agricultural machine renting and its impact on cereal crops productivity and commercialization which helps to draw a clear picture for policymakers involved in the development and dissemination of agricultural mechanization**.**

2. LITRATURE REVIEW

2.1 Theorthical Review

Agricultural mechanization is encompassed within the broader concepts of agricultural transformation and the development of farming systems. According to [40,41], the demand for mechanized technology by farmers is a consequence of the process of agricultural intensification. [41] is well-known for establishing a theoretical and empirical connection between population growth, technological innovation, and agricultural intensification. In order to promote agricultural intensification, new technologies are developed and implemented, such as increased use of inputs like labor and fertilizer, as well as the integration of non-human sources of power such as animal traction or machinery.

Boserup's discussion of mechanization does not extend to consider the source of mechanized technology, nor the relative prices of land, labour, and outputs. The only effort in existing literature to conceptualize the circumstances under which farmers opt for mechanized technology, rather than agricultural intensification in general, is seen in the application of the induced innovation hypothesis by [42]. The demand for labor- or land-intensive technology by farmers is influenced by the relative costs of land, labor, capital, and output, which encourages public or private research institutions to innovate and adjust their development of new technologies to meet the needs of farmers.

2.2. Empirical Review

Different studies have been conducted on the determinants of the adoption of agricultural technology and its impact on productivity, cost of production and households' welfare. However, there is no specific study about the impacts of agricultural mechanization on crop productivity in Ethiopia.

According to a study regarding the effect of farm mechanization on productivity of rice farms in southern Ghana, the productivity of rice differs depending on the type of mechanization intensity. In this particular study, households who used mechanization from tillage, threshing, and transportation had the most significant and positive relationship with productivity. Moreover, land size cultivated, agrochemical expenditure, tillage intensity, education and transportation were significant factors that positively influenced productivity of rice [43]. [44]

assessed the impact of farm mechanization on the productivity of wheat and maize crops in Pesh Awar valley. The result of Cobb-Douglas production function analysis revealed if the land of wheat and maize increases by one hectare, the productivity increases by 25.32 mounts for mechanized farmers to 23.22 mounds for non - mechanized farmers.

[13] examines the state, drivers and, consequently, the impacts of agricultural mechanization in eleven countries in Africa. Significant drivers of agricultural mechanization include the size of the household, gender of the household head, participation in off-farm economic activities, distance to the input and output markets, farm size, land tenure, type of farming system, access to extension services, and use of fertilizer and pesticides. His result showed that in general agricultural mechanization significantly raises the productivity of maize and rice in all cases. Similarly, [45] studied the impact of agricultural mechanization on agricultural production, income, and mechanism: evidence from Hubei province, China. This study analyzed the influence of agricultural mechanization level on agricultural production and income by utilizing a sample-modified endogenous merging model and a threshold effect model. The level of mechanization has a significant positive impact on the cost, output value, and income and return rate of all types of crops. For every 1% increase in the level of mechanization, the yields of all crops, grain crops and cash crops increase by 1.2, 1.56 and 0.43%, respectively.

2.3. Conceptual Framework

[40 and 41] explain agricultural intensification process needs mechanized technology, which is basically motivated by agro-ecological conditions, population density, and market demand. According to [20] demand for mechanization rose due to exogenous pushing factors of agricultural intensification, rising rural wages, urbanization, population pressure. Besides, rapid migration of the youth from rural to urban areas has a consequence of shortage of farm labor and cost draft animals changing the pattern of farming practice [27,19] Availability of machinery service provider in the nearby area of smallholder farmers is among the first factors that are contributing for making renting decision. Internal factors such as demographic and socio-economic and external factor like population growth, technological change, and development of infrastructure, market institutions and policy and are assumed to determine the renting decision of farmers for mechanization services.

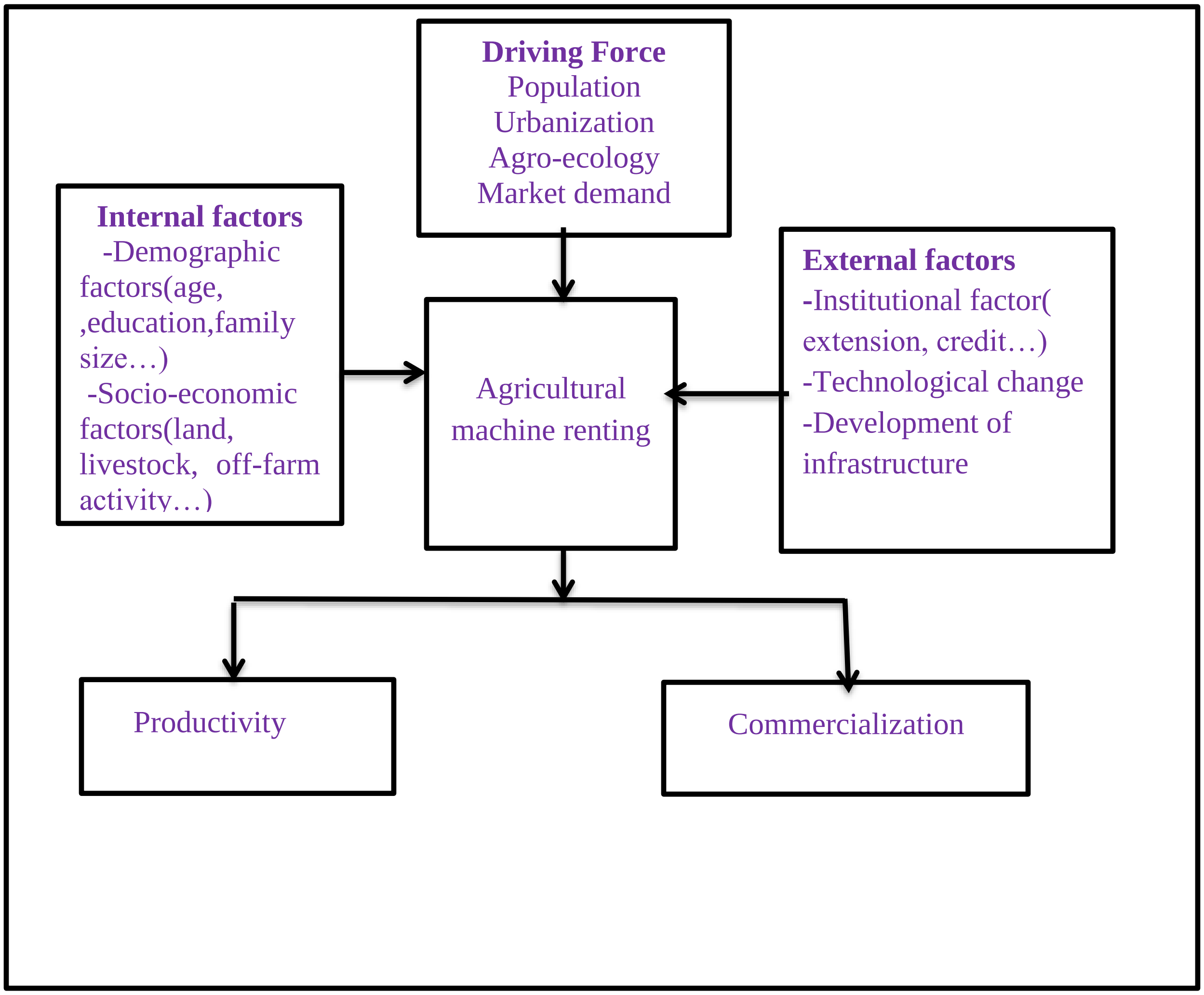

Source: based on literature

Figure 1: Conceptual framework

3. METHODOLOGY

3.1. Area Description

The study was carried out in the West Gojjam Zone of the Amhara region. West Gojjam zone, is one of the eleven (11) administrative zones established under the Amhara region. The administrative zone is bordered to the north by the Northern and Southern Gonder zones; to the east by East Gojjam zone; to the south by Oromia region and to the west by Benshangul Gumuz region. Agriculture system in West Gojjam zone is a combination of both crop and animal farming activities .the primary crops caltitivated in the area include teff, wheat,maize , pepper, fingermillet, bareley beans peas chiechpeas,among others.In the region cearals occupy 84.41% of the total agricultural land (637552 hectares) and account for approximately 89% the total crop yield(19,580,577.34) quintals.

In the West Gojjam Zone, farmers use different agricultural machines for ploughing, harrowing harvesting and threshing through renting agricultural machines. The agricultural machine renting service (tractor, combine harvester, maize Sheller) was started in 1980 and the number of agricultural machines used by farmers increased from time to time. These machine renting services are provided by a union, an agricultural mechanization corporation and privately through communication and agreement with machine owners from the Oromiya region. The total number of agricultural machines in the west Gojjam zone in the union and agricultural mechanization corporation are 84, 10 and 180 tractors, combine harvesters, maize Sheller respectively. Agricultural machine renting payment was different according to the situation due to road, distance to the market (station) and season etc. An average fee for ploughing, harvesting (threshing and winnowing), especially for wheat, and shelling for maize, was 3500 birr/ha, 130-150 birr/qt, and 20-30 birr/qt respectively [46]

3.2. Sampling Procedure and Sample Size Determination

A combination of purposive and multi-stage random sampling procedure was used to select districts, kebeles and sample households. Three districts from West Gojjam zone (Wonberma, Buriezuria and Dembecha) were selected purposively from the areas where agricultural machine rental practices took place. Then, in the first stage, a total of six kebeles were selected by simple random sampling method based on proportional to the number of Kebeles in each district. In the second stage households were stratified into two groups: those that adopted agricultural machines renting and those who did not. In the third stage, a representative sample size of 400 household heads were selected from both strata through systematic random sampling based on proportionate probability size of each districts and kebeles (145 households from Wonberma, 122 households from Burie Zuria and 133 households from Dembecha district)). Finally, 192 households that adopted at least one agricultural machine renting and 208 households that did not adopt an agricultural machine renting were obtained from six kebeles in the 2020/21 production season.as shown Table 1.

[47] method was used determine the sample size. Cochran's method is particularly suitable for stratified sampling designs, where the population is divided into distinct subgroups or strata, and is beneficial when the population size is large or unknown. The equation is written as:

$$n = \frac{z^2\,pq}{d^2}\,(eq)(1)$$

where n is a minimum sample size, Z is 1.96 at 95 % confidence level; p is smallholder farmers who may adopt in agricultural machine (50 %); q is the weight variable and is computed as 1-p), and d is the desired precision or margin of error, expressed as a fraction of 0.05.

Table 1.Selected districts, kebeles and their sample sizes

District	Sampled Kebeles	Total hhs	Sample hhs
Wenberma	Markuma	1029	69
	Koki	1124	76
Buriezuria	Alefa	989	67
	Wadra	808	55
Dembecha	Wade	659	45
	Asteboch	1300	88
Total		5909	400

3.3. Ethics Approval

The ethical approval for the study was obtained from the “Wolaita Sodo University ethical board and College of agriculture” and the research was carried out based guidelines of Wolaita Sodo University ethics statement. Therefore, the study offered an approval number of 356/16 by the ethical committee. We also obtained a survey study titled "agricultural machine renting and its impact on cereal crop productivity and commercialization: the case of smallholder farmers in West Gojjam zone, Amhara region, Ethiopia". Informed consent was obtained from all individual participants included in the study.

3.4. Data Types and Sources

Both quantitative and ~~qualitative data~~ were used in this study. both secondary and primary data sources were used to generate data types. Primary data sources are farmers who rent agricultural machines and non-users of these machines. Secondary data sources include journals, books, CSA and internet browsing, national policies, and zonal, district and kebele reports.

3.5. Data Collection Methods

The major data collection methods included formal surveys, discussions with key informants and focus groups.The primary data were collected though face-to-face personal interviews using a semi-structured questionnaire. The data were collected based on the research objectives. The questionnaire included both open and closed-ended questions to collect information relevant to the scope of the study. The questionnaire was first tested prior to the field survey for suitability (clarity, suitability and sequence of questions) and then re-tested based on the pre-test feedback.. In addition to interviews, discussion with each of the six selected kebeles extension agents who are working related to agricultural mechanization service (mechanization experts) were disscussed.

3.6. Methods of Data Analyses

Two types of data analysis methods were used to addressthe rereasch objective: descriptive statistics and econometric models.

Descriptive statistics

Descriptive analysis was used to analyze demographic, institutional and socio-economic characteristics of farm households. Further, descriptive statistics such as mean, proportion and standard deviation were used to describe economic and physical characteristics associated with households categorized based on their agricultural machine renting of mechanization behaviors. The independent sample t-test and chi-square tests were used to make comparisons between the categories with respect to key variables specified.

Measurement of cereal crop productivity

In this study, TFP is the ratio of output to the total variable cost (TVC). This approach Total Fixed Cost (TFC), does not role since it is constant and does not have any effects the on the profit maximization and resource-use efficiency conditions [48]. According to [49] and [50] TFP for cross-sectional data or data within a specified time can be measured as the inverse of the unit variable cost because TFP is the ratio of the output and the Total Variable Cost (TVC) as shown in this equation;

$$TFP = \frac{Y}{TVC} = \frac{\sum_{i=1}^{n} Y}{\sum_{j=1}^{n} PijXji} \qquad eq(2)$$

$$TFP = \frac{Y}{TVC} = \frac{1}{AVC}$$

Where, Yi : The output of cereal crops (in value or in quantities);

TVCi: Total Variable Cost of farm i;

Pji: Unit price of jth variable input used in farm i;

Xji: Quantity of jth variable input used in farm i.

Measurement of market participation

Measuring Crop Output Market Participation Index (COMPI): . Agricultural commercialization is the proportion of agricultural production that is marketed and the households that sell more proportions of his/her product could be considered as commercialized farmer [51]. The meaning of commercialization goes beyond supplying output ~~product~~ to the

market and it has to consider both the input and output sides of production [52]. In line with this, smallholder commercialization occur when farmers participate in agricultural markets either as a buyer (input) and/or seller (output) [53]. In this paper focused on output side of commercialization (market participation). Many empirical researches conducted on commercialization level crops [54,55,56,39]. At the household level, one definition of agricultural commercialization is defined as the ratio of the value of agricultural outputs sold to the total value of agricultural outputs produced by a household [57,58,59]. Hence, output side commercialization index or market participation for a household (COMPI) can be computed as:

$$COMPL_K = \sum_{i=1}^{N} \frac{S_{ki}}{Q_{ki}} \quad ; \quad Q_{ki} \geq S_{ki} \qquad \text{eq (3)}$$

Where, $COMPI_k$ is the proportion of crop k sold (S_{ki}) to the total amount produced (Q_{ki}) aggregated over the total sample households in a given location.

Efficiency and productivity analysis

The study used the production data of cereal crops (teff, wheat and maize) in the 2020/21 production season. The stochastic frontier methodology was later developed by[60,61]. It is specified as

$$y_i = f\left(x_{i,}\beta\right) + \varepsilon_i(v_i - u_i) \qquad eq(4)$$

Where: Yi is the amount of the cereal crops yield produced by ith household in the 2020/21 production year that is expressed in quintals; is a production function $f(x_{i,}\beta)$are the parameters to be estimated; X's are exogenous input factors (labour, land, fertilizer seed and herbicide) affecting the amount of yield; $\varepsilon i$is the error term, equal to ($Vi - Ui$); Vi is a two-sided random error component beyond the control of the farmer, and $Ui$is one-sided inefficiency component.

Given the level of input, the TE of the ith cereal crop producer farmers are the ratio of the observed output to the maximum potential output (frontier output):

$$TE_i = \frac{actual\ output}{potential\ output} = \frac{f(x_i;\beta)\exp(v_i - \mu_i)}{f(x_i;\beta)\exp(v_i)} = \exp(-\mu_i)$$

3.7. The impacts of agricultural machines renting on productivity and commercialization

To measure the impact of agricultural mechanization on the productivity and commercialization of cereal crops, endogenous switch regression (ESR) and propensity score matching (PSM) models were employed.

When assessing the impact of adopting an agricultural machine on the productivity commercialization of a farm household, it is important to capture both the observable and unobserved characteristics of the adopter (treatment group) as well as the non-adopter (control group). However, the majority of impact assessment methods that use non-experimental data do not capture the observable and/or non-observable characteristics that influence adoption as well as outcome variables. For example, instrumental variables only capture unobserved heterogeneity but assume that the parallel shift in outcome variables can be seen as a treatment effect [62,63,64].

In contrast, using regression models to analyze the impact of a given technology using pooled samples of users and non-users might be inappropriate because it gives the similar effect on both groups [64]. A methodological approach that overcomes the aforementioned limitations is ESR, which is the most frequently used common method to analyze the impact of a given technology [65,64,66]. In this paper, we employ parametric ESR with non-parametric PSM technique to reduce the selection bias and assure consistent results by capturing both the observed and unobserved heterogeneity that influence the outcome variable as well as the adoption decision.

The decision to adopt a technology can be modeled in a random utility framework by expressing the unobservable utility from adoption and non-adoption through observable variables[67].We specify the selection equation for technology adoption as:

$$\boldsymbol{P_i^*} = Zi\beta + U_i \qquad P = \begin{cases} 1, & if\ p *> 1 \\ 0, & otherwise \end{cases} \qquad eq(5)$$

Where$\boldsymbol{P_i^*}$: is unobservable or latent variable for machine renting, Yi is its observable counterpart (the dependent variable use of agricultural machines equals one, if the farmer has adopted at least one agricultural machine during 2020/21 cropping season, and zero otherwise), Zi are non-stochastic vectors of observed farm and non-farm characteristics determining adoption and Ui is random disturbances associated with renting of agricultural machine of improved technology.

Farmers use at least one of the agricultural machines (tractor, combine harvester, and maize Sheller) in their farming activities. However, according [66] if the selection equation (first stage) is endogenous in the outcome equation (second stage), the result would be biased and inefficient. Therefore, it is vital to use instrumental variable methods to identify the second stage equation from the first stage equation. The instrumental variable should affect the adoption of agricultural machines renting but not the outcome variables, such as productivity and commercialization. The study use access to information about agricultural machine (yes=1) as a selection instrument. Thus, this variable is more likely to be correlated with the adoption of agricultural machines, but not productivity and commercialization outcome variables or correlated with the unobserved. Moreover, we also check the validity of the instrument variable using a falsification test. The test shows that the variable significantly affects the adoption decision but not our outcome variables.

The two regimes that the smallholder farmers fall in to are represented by the following two regression equations.

$$Regime\ 1: Y_I = \alpha_1 X_i + e_{1i} \qquad if\ p = 1 \qquad eq(6)$$

$$Regime\ 2: Y_2 = \alpha_2 X_i + e_{2i} \qquad if\ p = 0 \qquad eq(7)$$

Y1i and Y2i are the dependent outcome variables determined by the exogenous variables Xi, α1, and α2, are parameters that show the direction and strength of the relation between the outcome variable and the independent variables. e_{1i} ande_{2i} are error terms.

Finally, the error terms are assumed to have a trivariate normal distribution, with zero mean and non-singular covariance matrix expressed as:

$$cov(u_i, e_{1i}, e_{2i}) = \begin{bmatrix} \sigma^2 u & . & . \\ \sigma^2 e_1 u & \sigma^2 e_1 & . \\ \sigma^2 e_2 u & . & \sigma^2 e_2 \end{bmatrix} \qquad eq(8)$$

Where $\sigma^2 u$ variance of the error term in the selection equation, $\sigma^2 e_1$ and $\sigma^2 e_2$ are variances of the error terms in the continuous equations. $\sigma^2 e_1 u$and$\sigma^2 e_2 u$ are covariance of $u_i, and\ \ e_{1i}\ and\ e_{2i}$respectively. Since Y_{1i} and Y_{21} are not observed simultaneously a covariance of the corresponding error terms is not defined [68]. This structure of the error terms indicates that the error terms of the outcome equation and the error term of the selection equation are correlated which results in non-zero expected value of e_{1i} and e_{2i} given ui - error term of the selection equation [65]. Therefore, the expected values of the truncated error terms E(e_1 | p = 1) and E(e_2 | p = 0) are given below:

E (e_1 | p = 1) = E(e_1 | U > - Zβ)

$$+= \sigma e_1 u \frac{\varphi(\frac{Z\beta}{\sigma})}{\Phi \frac{Z\beta}{\sigma}} = \sigma e_1 u \lambda_1 \qquad eq(9)$$

E (e_2 | p = 1) = E(e_2 | U ≤ - Zβ)

$$= \sigma e_1 u \frac{-\varphi(\frac{Z\beta}{\sigma})}{1 - \Phi(\frac{Z\beta}{\sigma})} = \sigma e_2 u \lambda_2 \qquad eq(10)$$

φ and Φ are the probability density and cumulative distribution function of the standard normal distribution, respectively. The ratio of φ and Φ evaluated at Zβ is referred to as the inverse Mills ratio $\lambda 1$ and $\lambda 2$ (selectivity terms). If the estimated covariance $\sigma^2 \varepsilon 1u$ and $\sigma^2 \varepsilon 2u$ are

significantly different from 0 the decision to renting agricultural machine and the outcome variable (productivity and commercialization) are correlated. This implies endogenous switching and the presence of a sample selectivity bias [69]. Further, estimations of treatment effects will be made. Average Treatment effect on the Treated and Untreated (ATT and ATU) are computed using the results for expected values of the dependent variable for users and non-users in actual and counterfactual scenarios:

$$E(Y_{1i}|p_i = 1, X_{1i}) = \alpha_1 X_{1i} + \sigma e_1 u \rho_1 \frac{\varphi(z\beta)}{\Phi(z\beta)} \qquad eq(11)$$

$$E(Y_{2i}|p_i = 0, X_{2i}) = \alpha_2 X_{2i} - \sigma e_2 u \rho_2 \frac{\varphi(z\beta)}{(1 - \Phi(z\beta)} \qquad eq(12)$$

$$E(Y_{2i}|p_i = 1, X_{1i}) = \alpha_2 X_{1i} + \sigma e_2 u \rho_2 \frac{\varphi(z\beta)}{\Phi(z\beta)} \qquad eq(13)$$

$$E(Y_{1i}|p_i = 0, X_{2i}) = \alpha_2 X_{2i} - \sigma e_1 u \rho_2 \frac{\varphi z\beta}{(1 - \Phi(z\beta)} \qquad eq(14)$$

ATT is the difference between the expected value of the outcome variable from Equations 11 and 13; this is the difference between the expected value of the dependent variable for users and if they were not used. ATU is the difference between Equations 12 and 14 which estimates the difference between the expected value of the outcome variable for non-users of agricultural machine.

PSM techniques were applied to supplement the ESR model and to assess the consistency of the results with different assumptions such as nearest neighbour matching, caliper and radius matching, and kernel and local linear matching [70]. The treatment effects will be estimated based on matching estimators selected on the common support region [71]. The average treatment effects can be estimated using the inverse propensity weighing estimates as stated in IPSW [72] using matching techniques of Kernel Matching (KM), Nearest Neighbor Matching (NNM) and Radius Caliper Matching (RCM).

The selection of a particular approach depends on the data in question and, in particular, the degree of similarity between the treatment and control groups in terms of propensity score [73].The Average Treatment Effect of an individual i can be written as:

$$ATE = E(Y_i^1 | D_i = 1) - E(Y_i^0 | D_i = 0) \qquad eq(15)$$

Where ATE, Average Treatment Effect, which is the effect of treatment on the outcome variables:

$E(Yi^1 | Di=1)$: Average outcomes for individual, with user, if he/she would participant
$E (Yi^0 | Di=0)$: Average outcome of non-user, when he/she would non-participant

Variables Definition and Hypotheses

Adoption of agricultural machine renting: A dichotomous dependent variable measured in participation of agricultural machine renting, taking value 1 if there is participation in agricultural machine renting and 0 otherwise.

The explanatory variables:

Age of household head: Is a continuous explanatory variable that measures the number of years of the household head. According to [74] when the household age has increased, the probability of technology adoption decreased. This is as a result of limited planning horizons when households are getting old. As a result, age of the household is hypothesized to affect agricultural technology adoption (tractor, combine harvester and maize Sheller) negatively

Family size: It is the total number of family members, which is measured in terms of adult equivalent**.** Larger family sizes would be expected to decrease the probability that a farmer will use a combine harvester [38]. Consequently, family size would have a negative influence on the adoption of agricultural machines renting.

Education level of household head: Is a continuous independent variable measured in number of years attended formal education. Educated farmers would have the ability to perceive, interpret and respond to new information much faster than their counterparts without education

[75] As a result, education is expected to correlate positively with the adoption of agricultural renting machines.

Total cultivated land: is a continuous variable and measured in hectares. It is frequently argued that farmers with larger farms are more likely to adopt improved technology (especially modern varieties) compared with those with small farms. According to [76] farm size of cultivated area of land has a significant influence on technology adoption decisions for wheat production. Therefore, farm size is expected to affect the adoption of agricultural machines renting positively.

Credit use: It is a dummy explanatory variable that takes the value of one if a household head has use of credit service and zero otherwise. Credits are expected to enhance farmers' financial capacity to purchase productivity enhancing. Use of credit services has a significant influence on technology adoption of wheat production [77]. So, a farmer who uses credit would have a positive influence on the adoption of agricultural machines.

Extension contact: This refers to the number of contacts per year that the household made with extension agents, and it is a continuous variable. The effort to disseminate new agricultural technologies is within the field of communication between the extension agent and the farmers at the grass root level [78]. Here, the frequency of contact between the extension agent and the farmers is hypothesized to be the potential force which accelerates the effective dissemination of adequate agricultural information to the farmers, thereby enhancing farmers' decision to adopt new crop technologies. As a result, frequent contact with extension agent would have a positive influence on the adoption of agricultural machines.

Access to information: It is measured as a dummy variable taking a value of one if the farmer had access to agricultural machine information from an extension agent, cooperative and farmers and zero otherwise. Farmers who had information from extension agents were more likely to adopt soil and water conservation practice 79]. Better agricultural machine information would help farmers to get detailed information about benefit, renting cost, source of agricultural machine.

Off-farm activity participation: This is a dummy explanatory variable that takes a value of one if a household head participated in off-farm activities and zero otherwise. Households with higher off-farm income may reduce the time allocated to farm management and resulting in lower productivity [80]. Therefore, participation in off-farm activity would have negative influence on the adoption of agricultural machines.

Distance to the nearest market: It is measured in kilometers. Distance to the nearest market is likely to influence the adoption of agricultural machines. The closer they are to the nearest market (station), the more likely that the farmers will receive valuable information and get machines. Farmers that are located far away from the nearest markets are less likely to adopt farm inputs than farmers who live near to market due to transaction cost [54]

Distance from main road: It is a continuous explanatory variable and measured in kilometers. Distance to the main road is used as a proxy for accessibility of transportation to markets. Distance from main road far from farmers residence renting of agricultural machines more expensive since due to transport cost and time [19]. Hence, households far away from roads are expected to be less probability of machine adoption.

Distance to the development agent: It is measured in kilometers. Proximity to development agents' office has the advantage of farmers gaining awareness and expose to new ideas and information about productivity inputs and outputs [81]. Households residing in places far from development agents' office were less likely to adopt agricultural machine than those who live near to development agents' office because of higher transaction costs, or may be information gap. This might be the cost of agricultural machines directly expressed in terms of distance from the station. Thus, the adoption status of agricultural machines would be reduced.

Position of farmer in farmer's association: it is a dummy variable coded as 1 if farmers have a position in rural kebeles, or model farmers and, 0 otherwise. Farmers who have some position in rural kebeles or model farmers are more likely to be aware of new practices as they are easily exposed to information [82]. Therefore, farmers who have a position in the association are hypothesized to have a positive relation with the adoption of agricultural machines renting.

Number of oxen: It is a continuous variable measured as the number of oxen owned by the household heads. All agricultural tasks are not performed only by agricultural machines renting; it also needs other additional oxen [83]. Thus, ownership of oxen has a positive influence on agricultural machine renting adoption.

Livestock (excluding oxen): is a continuous variable measured in terms of Tropical Livestock Units (TLU) of livestock owned by the household excluding oxen. Livestock is the farmers' important source of income, food and draft power for crop cultivation in Ethiopian agriculture. It was hypothesized that as livestock ownership increases, adoption/intensity of adoption is expected to increase because it serves as proxy for wealth status [82].

Membership to cooperative: It is a dummy variable is measured by allocating a score of 1 if a farmer is a member of an organization and 0 otherwise. Belonging to a member of a cooperative influences a farmer’s decision to adopt an improved technology. Being a member of the cooperative has a significant and positive impact on the adoption of teff row planting [84]. Therefore, farmers’ membership of a cooperative would have a positive influence on the adoption of agricultural machines.

Outcome variable

Total factor productivity: Total factor productivity index of cereal crops includes (teff, wheat and maize) produced by households in the 2020/21 production year.

Technical efficiency: Technical efficiency of cereal crops (teff, wheat and maize) in the 2020/21 production season.

Output commercialization: Cereal crop output commercialization indices include teff, wheat and maize output commercialization indicesin the 2020/21 production season.

4. RESULTS AND DISCUSSIONS

4.1. Demographic, Socio-Economic and Institutional Characteristics of households

The results statistical summary that shows the difference between machine users and non-users for demographic, socio-economic and institutional characteristics of households (Table 6). The average ages of household heads for renters and non-renters were 46.69 and 42.91 respectively. The result of the t-test showed that age of household heads had a significant mean difference between renters and non-renters at less than 1% probability level. The aged household heads were adopted agricultural machine renting better than the young aged. These maybe aged household heads have better experience and knowledge about the importance of agricultural machine renting than the young aged household heads. The overall household heads education level was an average of 3.78 years. The study result shows that the farmers who adopted agricultural machines were relatively more educated (with an average of 4.19 years and 3. 39 years for non- adopters) and the difference was statistically significant at less than 1 % probability level. Family size was another variable that had a significant mean difference between renters and non-renters at less than 5% probability level.

Moreover, the result of the t-test revealed that total land and ownership of oxen had significant mean difference between renters and non-renters at less than 1% probability level. Household heads that adopted agricultural machine renting have a comparatively larger farm size and a greater number of oxen. The study result shows that farmers who adopted agricultural machine renting reported higher frequencies of contact with extension agent than non-adopters. Non-adopter farmers were located far from the nearest markets (3.48 Km) compared to adopters (2.87Km). Distance from the nearest market had a significant mean difference between agricultural machine renting renter and non-renters at less than 1% of significance level. This shows that farmers who lived nearest to market centers adopted agricultural machine renting than those who were located far from market centers.

Table 1. Demographic, socio-economic, institutional and pilot characteristics of households by agricultural machine renting for continuous variable

Description	Machine renting N=192	Not Machine renting N=208	Total	t-test
Age of household head	46.69 (0.74)	42.91 (0.61)	44.72 (0.48)	3.98***
Education level	4.19 (0.22)	3.39 (0.21)	3.78 (0.15)	2.61***
Family size	4.86 (0.12)	4.53 (0.11)	4.69 (0.08)	-1.92**
Distance from market	2.87 (0.13)	3.48 (0.18)	3.19 (0.12)	-2.55***
Distance from road	2.34 (0.11)	2.4 (0.09)	2.38 (0.09)	0.35
Distance from DA	2.22 (0.09)	2.35 (0.09)	2.29 (0.06)	1.01
Extension visit	12.4 (0.51)	9.76 (0.35)	11.03 (0.31)	4.34***
Total land	2.41 (0.07)	1.89 (0.05)	2.14 (0.04)	5.88***
Own land holding	1.6 (0.06)	1.35 (0.05)	1.47 (0.04)	3.12***
Number of oxen	2.7 (0.07)	2.1 (0.05)	2.39 (0.04)	6.43***
Total livestock holding (TLU) excluding oxen	4.18 (0.16)	3.99 (0.15)	4 (0.1)	-1.03

The chi-square test results for categorical variables were given in Table 7. It indicated that power in social activities and use of credit had a significant mean difference between agricultural machine renting adopters and non-adopters at less than 1 % probability level. About 63.59% of non-renters farmers had power in social activity whereas 36.41% of farmers were renters. Household heads who adopted agricultural machine renting had less power to do social activity compared to non-renters. In addition, the chi square test showed that farmers who adopted agricultural machine renting were fewer users of credit. Moreover, participation in off-farm activity and access to information from extension agent about agricultural machine renting had significant differences between agricultural machine renting renters and non-renters at less than 10% probability level.

Table2. Demographic, socio-economic, institutional characteristics of households by agricultural machine renting for categorical variable

Variables	Level	No.	%	No.	%	x^2-value
Sex	Male =1	195	51.45	184	48.55	0.87
Power on social	Yes	71	36.41	124	63.59	37.0***
Member-coop	Yes	168	52.66	151	47.34	0.28
Credit use	Yes	72	41.62	101	58.38	13.16***
Off-farm parti	Yes	76	58.02	55	41.98	2.82*
Information EA	Yes	155	54.96	127	45.04	3.37*

Source: Computed from survey data (2021)

4.2. Agricultural machine renting

In the study area, tractors, combine harvestesrs and maize Sheller are used in the production process of cereal crops, either solely or simultaneously. The study showed that Table 4 approximatly 25.3% rented tractors only for ploughing services. Farmers commonly rent tractors in the study area for the first and second tillage. This is becauset the first ploughing operation is the heaviest and most challenging for oxen, whereas a tractor can plough easily and deeply. Similarly, about 22.3% of farmers rented combined harvesters only for wheat harvesting, threshing and winnowing operation and about 28.54% rented maize Sheller machines only for shelling of maize after harvesting. About 11.16% of farm households rent three types of machines (tractor, combine harvester and maize Sheller) simultaneously.

Table 4.Major agricultural machine used in the study area

Types of agricultural machines used	Freq.	Percent
Tractor	118	25.3
Combiner	104	22.3
Sheller	133	28.54
All (tractor, combiner and Sheller)	52	11.16
Tractor &combiner	17	3.65
Tractor & Sheller	20	4.3
Combiner & Sheller	22	4.72

4.3. Impacts of agricultural machine renting on productivity and commercialization

The essential tests under econometric analysis that verified the model were undertaken on hypothesized variables. The variables included in the model were tested for the problems of multicollinearity and heteroskedasaticity. The result shows that there is no problem of multicollinearity and heteroskedasaticity among the variables. Further, the model specification was carried out using the Ramsey-reset test and the results revealed that there were no omitted variables in the model (see from appendix).

Table 5 represents the first stage ESR binary probit estimation results. The probit model fits the data reasonably well [Wald Chi-squared = 137.31, p= 0.000)]. The model results reaveled that household, socioeconomic, and institutional factors had a significant impact on the decision to rent agricultural machines.

The age of the household head affected adoption of agricultural machine renting positively and significantly at 1% level. The marginal effect result indicates that aged household heads adopt more agricultural machines than younger households because, as the age of households increases, working power decreases. This finding is similar to [13]. Agricultural machine renting increased with the level of education of the household heads. Household heads who attened better education level have a good awareness about agricultural machines. Educated household heads are more likely to use agricultural machines [10,39]. The total land cultivated significantly and positively affected the adoption of agricultural machines. Farmers who owned large amount of cultivated land were more likely to adopt agricultural machines than small land-holding farmers. [63] found farm size was a positive influence on the adoption of agricultural technology packaging. Ownership of large amounts of cultivated land often required more labor and time to perform agricultural operations.

Frequency of extension contact influenced the adoption of agricultural machines significantly and positively at the 1% level. This implies that farmers who are frequently in contact with extension agents are more likely to adopt agricultural machines than are those in lower contact with extension agents. This is because, development agents provide advice on agricultural machine utilization and benefits to farmers. The extension provides in-depth information, training and consultancy services on the source, application and relevance of agricultural

technologies for farmers. Similarly, farmers with information about agricultural machines from government extension agents are more likely to adopt agricultural machines. This is because the provision of information helps farmers to become aware of the benefits and costs of machines. The use of credit negatively and significantly affected the probability of farmers using agricultural machines to produce cereal crops at 1%. The result indicated that farmers who used credit services decreased the probability of agricultural machines renting. This may be because households used credit for consumption, fattening livestock and home construction.

Distance from the market and the main road negatively and significantly affected the adoption of agricultural machines by 10% and 5%, a significant level. Proximity to the market and main roads are important factor in adopting agricultural machines because better access to markets and roads is an indicator of access to information and infrastructure. Thus, farmers who live near the market and main road are more likely to rent agricultural machines and other important inputs at a lower cost than those who live far away from markets and main roads. Similarly, [85] the adoption decision of smallholders affected by distance from the marketplace due to cost of production. The number of oxen affects the adoption of agricultural machines positively and significantly at 1%. This implies an increase in the number of oxen and the probability of adopting agricultural machines increases. This means that households with many oxen perform agricultural activities like the second plow, third plow, sowing and threshing more easily than thos fewer oxen.

.

Table 5. Decision of adopting agricultural machines renting: Probit model

Variables	Coeff	Std.Err	Marginal Effect
Age of household	0.03***	0.009	0.012
Education level of household	0.09***	0.026	0.036
Adult equivalent	-0.11	0.059	-0.045
Poweron social activity	-0.49***	0.149	-0.196
Market distance	-0.07*	0.043	-0.031
Road distance	0.14**	0.073	0.058
Distance from office	0.1271	0.086	-0.050
Extension visit	0.0376***	0.012	0.015
Member of cooperative	0.2692	0.185	0.107
Credit use	-0.4276***	0.146	-0.170

Total land	0.3655***	0.040	0.145
TLU	-0.0372	0.041	-0.014
Number of oxen	0.3934***	0.110	0.156
Off farm activities	-0.0666	0.158	-0.026
Information for mechanization	0.3425**	0.165	0.136
-Cons-	-2.1487***	0.705	

Source: Computed from survey data (2021)

The results of the second stage ESR and,the estimated coefficients of total factor productivity, technical efficiency and commercialization index are presented in the appendix

Table 6 shows the results ATT and ATU for the key outcome variables related to agricultural machine renting. In this analysis the main outcome vaiables are TFP, TE and COIM index. Before, we analyzed the result of ESR model to check the validity of instrumental variables using a falsification test. The falsification test shows the instrumental variable significantly affects the adoption of agricultural machines but not TFP, TE and COIM index. Instrument variables are jointly statistically significant in the adoption equation [$\chi2 = 4.22$ ($p = 0.04$)] but not outcome functions. The instrumental variable did not affect the TFP [$F=0.10$($p= 0.7497$)] technical efficiency [$F=0.45$ ($p=0.5038$)], and commercialization index [$F= 1.92$ ($p=0.1668$)].

The adoption of agricultural machines was renting increases total productivity, technical efficiency and commercialization level of farm households. For farmers who adopted agricultural machine renting but theoretically would have not adopted, then their TFP decreased by 0.573 (57.3%). This means, the ATT of total factor productivity of cereal crops would decrease by 57.3% as a result of did not use agricultural machines renting. Likewise, if non-users of agricultural machine adopted agricultural machine renting, then their TFP would have significantly increased by 0.419(41.9%). For farmers who adopted agricultural machines but theoretically would not have adopted them, then their TE decreased by 0.104 (10.4). This means, the ATT of TE on cereal crops would decrease by 10.4% due to do not adopting agricultural machines. Similarly, if non-users of agricultural machine adopted agricultural machines, then their TE would have significantly increased by 0.064(6.4%). In addition, the results of the commercialization index of cereal crops for adopters would drop by 0. 12(12%) if the adopters

were not adopted. In the same way, if the non-users of agricultural machine had adopted agricultural machine renting, their COIM would have increased by 0.098 (9.8%).

Consequently, ESR shows that in Ethiopia the adoption of agricultural machine renting would have increased productivity and commercialization of cereal crops. This result resembles to [3] the adoption of agricultural technology has a positive effect on crop productivity including wheat. The adoption of agricultural technologies has also a positive and significant impact on productivity of teff [84].

Table. 6 Endogenous switching regression model result (average treatment effects)

Outcome variables	Mean farm household type and treatment effect	Decision stage		Treatment effect
		To Adopt	Not to Non-adopt	
Productivity	ATT	3.1	2.52	0.573***
	ATU	3.13	2.7	0.422***
TE	ATT	0.845	0.741	0.104***
	ATU	0.837	0.772	0.064***
COIM	ATT	0.572	0.452	0.120***
	ATU	0.544	0.444	0.099***

Note. ATT = Adoption effect for adopters, ATU = Adoption effect for non-adopters, and *** p <.01.

Popensity Score Matching Estimation Results

In addition to the ESR model, this study uses the PSM technique to check the robustness of the results obtained from the ESR model. Propensity scores (the probability of adoption in agricultural machine) are estimated using a probit model. Figure2 shows the distribution of adopter and non-adopter households with respect to estimated propensity scores. The figure illustrates the estimated propensity distribution for treatment and control households. The upper half of the graph refers to the propensity score distribution of treatment groups, while the bottom half shows the control groups. The y-axis refers to the densities of estimated propensity scores.

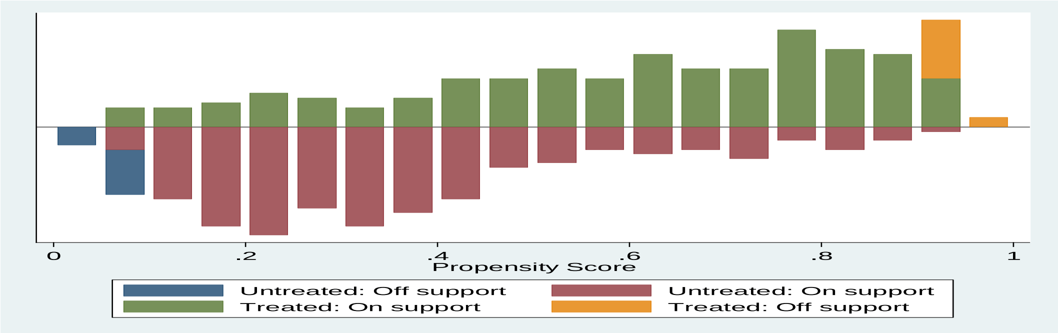

Figure 2: Distribution of estimated propensity distribution for treatment and control groups and common support area

There should be a common support condition that should be applied to how the propensity score is distributed between adopters of agricultural machine renting and those who do not. The estimated propensity scores varied between 0.0826423 and 0.9633814 (mean =0. 6249002) and 0. 0347458 and 0.92732443 (mean, =0.3462459) in the treatment and control groups, respectively. The common support lies between 0.0826423 and 0.92732443. Table 7 presents the balancing tests for each matching algorithm before and after matching. The results show that the mean standardized bias is reduced after matching from 10.3 to 5.3 percent) compared to before matching (38percent). Similarly, Pseudo-R^2 declined substantially, from 22.4 percent to 1 percent. The p-values from the likelihood ratio tests showed that all of the covariates were statistically significant at a p-value of less than 1 percent prior to matching, but insignificant post-match. These p-values clearly demonstrate that the matching process balance the observed characteristics between treated and control after matching.

Table 7.Covariates balancing tests before and after matching

Matching algorism	Pesido-R-square		LRx^2P −value		Mean standard bias	
	Before	After	Before	After	Before	After
NNM_1	0.224	0.010	123.87 (0.00)	4.99 (0.986)	38	5.3
NNM_2	0.224	0.014	123.87 (0.00)	6.71 (0.278)	38.	6.9
KBM_3	0.224	0.010	123.87 (0.00)	4.83 (0.729)	38	6.6
KBM_4	0.224	0.011	123.87 (0.00)	5.36 (0.980)	38.	6.5
RCM_5	0.224	0.030	123.87 (0.00)	8.55 (0.881)	38	7.8
RCM_6	0.224	0.030	123.87 (0.00)	10.22 (0.746)	38	10.3

NNM1 = One nearest neighbor matching and common support

NNM_2 = Two nearest neighbor matching and common support

KBM3 = Kernel with band width 0.05 and common support

KBM4 = Kernel with band width 0.1 and common support

RCM4 = Radius Caliper 0.05 matching

RCM5= Radius Caliper 0.1 matching

Table 8 shows the result of PSM, using three different matching algorithm techniques (nearest neighbor matching (NNM), Kernel based matching (KBM), and Radius matching methods). The result reveals that, as in the ESR analysis, the adoption of agricultural machine renting resulted in an increase in TFP, TE and COIM. The PSM result revealed that increased adoption of agricultural machines renting significantly increases total factor productivity of crops in the range of 0.41 to 0.52 (41 to 52 percent). Similarly, the adoption of agricultural machines increases technical efficiency from 0.077 to 0.104 (7.7% -10.4%). Moreover, on average, the adoption of agricultural machines increased the household's commercialization level in the range from 0.102 to 0.116 (10 to 11.6 percent). Therefore, it can be concluded that, apart from the minor differences in magnitude between the PSM and ESR estimates, the results of ESR and PSM indicate that the adoption of agricultural machines had positive impacts on TFP, TE and

COIM levels. This finding is similar to [79] the result from PSM and ESR estimates of soil and water conservation practice on food insecurity and net crop value.

Table 8.Average treatment effects: propensity score matching

Outcome variable	Matching algorism	Mean of outcome variables based on matched observation		ATE	ATT	SE
		Adopter	Non-adopter			
COMI						
	NNM_1	0.573	0.459	0.1105	0.114	0.015
	NNM_2	0.573	0.459	0.105	0.113	0.016
	KBM_3	0.573	0.46	0.105	0.113	0.016
	KBM_4	0.573	0.459	0.105	0.113	0.016
	RCM_5	0.555	0.456	0.098	0.100	0.013
	RCM_6	0.567	0.451	0.108	0.116	0.011
	NNM_1	3.10	2.57	0.46	0.524	0.104
TFP	NNM_2	3.10	2.6	0.44	0.49	0.102
	KBM_3	3.10	2.58	0.44	0.511	0.093
	KBM_4	310	2.58	0.45	0.513	0.090
	RCM_5	3.12	2.64	0.46	0.47	0.080
	RCM_6	3.11	2.68	0.39	0.411	0.066
	NNM_1	0.847	0.753	0.089	0.093	0.020
	NNM_2	0.847	0.745	0.089	0.102	0.017
TE	KBM_3	0.847	0.749	0.085	0.098	0.015
	KBM_4	0.847	0.751	0.84	0.096	0.015
	RCM_5	0.847	0.773	0.073	0.077	0.010
	RCM_6	0.847	0.774	0.070	0.077	0.009

5. SUMMARY

The agricultural sector in Ethiopia is the main source of economic development, and its growth originates from smallholder farmers. Even though smallolder agricultural production and productivity still low. Thus, this study analyzed the of impact agricultural mahines on the productivity and commercialization of cereal crops in West Gojjam, Ethiopia. A series of formal and informal surveys were carried out during the 2020/2021 crop season on 400 sample farm household heads that were randomly drawn from six kebeles in three woredas through a multi-stage sampling procedure. Secondary data were collected from published and unpublished sources to support the primary data.

Descriptive statistics and econometric models were used to analyze the data. The descriptive statistics include the mean, percentage, t-test and chi-squre tests. Econometric models, including endogenous switch regression (ESR) and propensity score matching (PSM) were employed.

The descriptive statistics showed that approximately 48% sample of households were renting agricultural machines to perform different agricultural operations. The study also investigates the total factor productivity and technical efficiency of cereal crops by using the total factor productivity index and stochastic frontier function. The results showed that the total factor index of the cereal crop was 2.89 and the stochastic frontier model showed that technical efficiency of cereal crop producers was 80.6%. The impact of agricultural machine renting on productivity and commercialization was estimated using endogenous switch regression and propensity score matching models. In these models, 192 machine users and 208 non-users of agricultural machines were used. The results of the first ESR model showed that age, education level, ownership of oxen, total cultivated land, extension visit, access to information, family size, the position a farmer, and distance to the nearest market significantly affected adoption of agricultural machine renting. The results of the endogenous switch regression and propensity score matching model indicate that the adoption of agricultural machines has positive and significant impacts on total factor productivity, technical efficiency and output commercialization index.

5.1. Policy implication

The adoption of agricultural machines has a positive and significant impact on the productivity and commercialization of cereal crops. This result indicates that agricultural mechanization enhances productivity and commercialization of cereal crops in the study area. When agricultural mechanization increases productivity and commercialization, we should examine what factors determine agricultural machine renting. Based on the result of study determined by demographic, socio-economic and institutional factors. Therefore, Therefore, governmental and non-governmental stakeholders give more emphasis on:

- formulating appropriate policies to provide adequate and effective basic educational opportunities for rural farming households.
- deliver selective and appropriate agricultural machines to smallholder farmers
- creating better extension services and offering practical training or demonstration on the utilization of agricultural machines for plough, harvesting, threshing and shelling of crops.
- strengthening the existing rural-urban market centers, rural-urban roads and other infrastructure development activities in the study area.

Finally, the government also should formulate a supporting policy for local importers of agricultural implements and strengthen their capacity.

Authors contributions:
Conceptualization, Selam Tilahun, Berhanu Kuma and Amsalu Bedemo
Methdolgy, Selam Tilahun
Validation, Berhanu Kuma and Amsalu Bedemo
Formal analysis, Selam Tilahun
Investigation, Selam Tilahun
Data curation, Berhanu Kuma and Amsalu Bedemo
Writing original draft,Selam Tilahun
Writing review and editing, Selam Tilahun
Visualization, Berhanu Kuma and Amsalu Bedemo
Supervision, Berhanu Kuma and Amsalu Bedemo
Availability of supporting data: the data presented in this study are available from the author upon request

Author Declaration: The authors declare no conflict of interest.

6. REFERENCE

1. World Bank Indicators Washington, DC, 2020. Author

2. CSA (Central Statistical Agency), Agricultural sample survey report on Area and production of major Crops (private peasant holdings, meher season 2016 / 2017; Addis Ababa Ethiopia, the FDRE statistical bulletin, Volume I.

3. Fisseha Z , Abrham S, and Dawit, A . Cereal production practices and technical efficiency among farm households in major "teff" growing mixed farming areasof Ethiopia:CogentEconomics&Finance, 2022;10:1, doi: 10.1080/23322039.2021.2012986

4. Doss C. R, W. M. Mwangi, H. Verkuijl, and H, De Groote. Adoption of Maize and Wheat Technologies in Eastern Africa: A Synthesis of the Findings of 22 Case Studies. International Maize and Wheat Improvement Center (CIMMYT) Economics Working Paper Mexico City,2020, http://repository.cimmyt.org/handle/10883/1037

5. Shita A, Kumar N, and Singh S. Agricultural Technology Adoption and its Determinants in Ethiopia: A Reviewed Paper. Asia Pacific Journal of Research,I(LVV) 2018, 99–104

6. Macauley H. Feeding Africa: Cereal Crops: Rice, Maize, Millet, Sorghum, Wheat. 2015; Vol. 86, AbdouDiouf International Conference Centre, Senegal, 74.

7. CIMMYT . Farm mechanization and conservation Agriculture for sustainable intensificatio(FACASI) project.Market analysis for small mechanization Ethiopia,2014 .

8. Dagninet A, and Wolelaw E. Agricultural Mechanization: Assessment of Mechanization Impact Experiences on the Rural Population and the Implications for Ethiopian Smallholders. Engineering and Applied Sciences, 2016; 1(2): 39-48.

9. Houmy K. M, F. Rojas, and C. Side,. Agri-hire in sub-Saharan Africa Business models or investing in sustainable mechanization Rome, https://doi.org/,2021,10.4060/cb5071en

10. Berhane G, Kalle H, and Bart M. Agricultural Mechanization in Ethiopia: Evidence from the 2015 Feed the Future survey, International Food Policy Research Institute.

11. Feder G, Just R. E, and Zilberman D. Adoption of agricultural innovations in developing countries: A survey. Economic development and cultural change, 1998;.33(2) :255-298

12. Adu-B F, Daum T,and Birner R. Can small farms benefit from big companies' initiatives to promote mechanization in Africa? A case study from Zambia. Food policy, 2019; 84, 133-145.

13. Kirui O. The Agricultural mechanization in Africa: micro-level analysis of state drivers and effects. ZEF-Discussion Papers on Development Policy, 2019 ; (272).

14. Yukichi Y, Mano, Yukichi Y, Takahashi K,Otsuka K. Contract Farming, Farm Mechanization, and Agricultural Intensification: the Case of Rice Farming in Cote d'Ivoire. Working Papers, 2017; vol. 157 JICA Research Institute.

15. Houssou N, and Chapoto A. Adoption of farm mechanization, cropland expansion, and intensification in Ghana, 2015. (No. 1008-2016-80214).

16. Kahan D, Bymolt R, and Zaal F. Thinking outside the plot: insights on small-scale mechanisation from case studies in east Africa. J. Dev. Stud, 2017;1–16.

17. Cossar F. Impact of mechanization on smallholder agricultural production: evidence from Ghana, 2019 (No. 2229-2019-1910).

18. Daum T, and Birner R. Agricultural mechanization in Africa: Myths, realities and an emerging research agenda. Global Food Security, 2020; 26, 100393. https://doi.org/10.1016/j.gfs.2020.100393

19. Berhane G, Dereje M, Minten B, and Tamru S. The rapid – but from a low base – uptake of agricultural mechanization in Ethiopia : Patterns, implications and challenges. International Food Policy Research Institute (IFPRI). ESSPWorkingPaper,2017; 105

20. Diao X, Cossar F, Houssou N, and Kolavalli S. Mechanization in Ghana: Emerging demand, and the search for alternative supply models. Food Policy, 2014.; 48, 168–181. https://doi.org/10.1016/j.foodpol.2014.05.013

21. Takeshima H. Market imperfections for tractor service provision in Nigeria: International perspectives and empirical evidence, 2016; 32. Intl Food Policy Res Inst.

22. NPC. 2016. Growth and Transformation Plan II (GTP II) (2015/16-2019/20), Volume I: Main Text

23 Tadesse W, Bishaw Z, and Assefa S. Wheat production and breeding in Sub-Saharan Africa: Challenges and opportunities in the face of climate change. *International Journal of Climate Change Strategies and Management*, 2019, 11(5), 696–715. https://doi.org/ 10.1108/IJCCSM-02-2018-0015

24 Belete A. Analysis of technical efficiency in maize production in Guji Zone: stochastic frontier model. Agric & Food Secure, https://doi.org/,2020,10.1186/s40066-020-00270-w

25 Yohannes M. T, Paulos A, and Dawit T. S. Factors affecting farmers’ hiring decisions on agricultural mechanization services: A case study in Ethiopia, Cogent Economics & Finance, 2023, 11:2, 2225328, DOI: 10.1080/23322039.2023.2225328

26. Agricultural Transformation Agency. Annual report of 2013/14 Addis Ababa

27. FAO and AUC. Sustainable Agricultural Mechanization: A Framework for Africa. Addis Ababa, 2018; 127pp. Licence: CC BY-NC-SA 3.0 IGO

28 Rosegrant M, W, Koo, J,Cenacchi N, Ringler C, Robertson R, Fisher M, Cox C, Garrett K, Perez N. D, and Sabbagh P. Food Security in a World of Natural Resource Scarcity the Role of Agricultural Technologies. Monographs of the Society for Research in Child Development, 2014; 79 (1)

29. Yared D. Agricultural mechanization, enabling policy, machinery suppliers, hiring services, heterogeneity, hiring cost doi: https://doi.org/,2021,10.21203/rs.3.rs-478878/v1

30. CSA (Central Statistical Agency). Agricultural sample survey report on Area and production of major Crops (private peasant holdings, meher season 2019 / 2020; Addis Ababa Ethiopia, the FDRE statistical bulletin, Volume I

31. Bantelay T, Tsehay D, and Nigist k . An integrated approach to solve small farm holder's mechanization barriers in Ethiopia. Computer science ,Social Telecomnication Enginering, .https:doi.org/,2020,10.1007/978-3030-43690-2_4.

32. Wudu B. Deteraminants of adoption of improved wheat technology: in case of Gozzamen district, east Gojjam in Amhara regional state, Ethiopia (thesis, St. Mary's University) ,2017.

33. Gebru A. The Determinants of Modern Agricultural Inputs Adoption and Their Productivity in Ethiopia (The Case of Amhara and Tigray Regions) (thesis, Addis Ababa University), 2016.

34. Worku A. Factors affecting diffusion and adoption of agricultural innovations among farmers in Ethiopia case study of Ormia regional state WestsernShewa. International Journal of Agricultural Extension, 2019;7(2):137–147. https://doi.org/10.33687/ijae.007.02.2648

35 Workineh M, Lakew T, and Haile K. E. Agricultural technology adoption and its impact on smallholder farmers welfare in Ethiopia. African Journal of Agricultural Research, 2020; 15(3): 431–445. https://doi.org/10.5897/AJAR2019.14302

36. Massresha E, Lema Z, Neway, and Degua A. Perception and determinants of agricultural technology adoption in north shoa zone, Amhara regional state, Ethiopia. Cogent Economics & Finance, 2021; 9(1), 1956774.https://doi.org/10.1080/23322039.2021.1956774

37 Tamirat N, and Abafita J. Adoption of row planting technology and household welfare in southern Ethiopia, in case of wheat grower farmers in Duna district, Ethiopia. Asia-Pacific Journal of Science and Technology, 2021; 26(2).

38. Hassena M, Regassa E, Mwangi H, and Verkuijl H. A Comparative Assessment of Combine Harvesting Vis-à-vis Conventional Harvesting and Threshing in Arsi Region, Ethiopia.

Mexico, D.F. (CIMMYT) and Ethiopia Agricultural Research Organization(EARO) , 2000.

39. Tamrat G, Mengistu K, Arega S, and Getachew L. Determinants of farm mechanization in centraland southeast oromia region, Ethiopia,2023.https://doi.org/10.1016/j.heliyon.2023.e18390

40. Boserup E. The Conditions of Agricultural Growth.The economics of agrarian change under population pressure. New Brunswick, USA: Transaction Publishers, 1965.

41. Pingali P, Bigot Y, and Binswanger H.P. Agricultural mechanization and the evolution of farming systems in Sub-Saharan Africa. Am. J. Agric. Econ 1987, 70 (2), 498.

42. Binswanger H, and Ruttan V. Induced Innovation. Baltimore and London: The Johns Hopkins University Press,1978.

43. Apiors E, Kuwornu J, and Kwadzo, G. Effect of mechanization use intensity on the productivity of rice farms in southern Ghana. Acta agriculture Slovenica, 2016,107(2): 439.

44. Din R,U, and Khattak N,U. Impacts of farm mechanization om wheat and maize productivity in Peshawar valley,Sarhad Journal of Agriculture, 2018,34(3)

45. Peng J, Zhao Z, and Liu D. Impact of agricultural mechanization on agricultural production, income, and mechanism: evidence from hubei province, china. front. environ. sci. 10:838686. doi: 10.3389/fenvs.2022.838686

46 .West Gojam West Gojjam Zone of Agriculture. West Gojjam Zone Bereau of Agriculture and Rural Development, Amhara Region, Ethiopia,2021.

47. Cochran, W.G. Sampling Technique. 2nd Edition, John Wiley and Sons Inc., 1963, New York.

48. Bamidele F.S, Babatunde R.O, and Rasheed A. Productivity analysis of cassava-basede production systems in the Guinea Savannah: Case study of Kwara State, Nigeria. American-Eurasian Journal of Scientific Research, 2008; 3 (1): 33-39.

49. Key N. and Mcbride W. Production contracts and productivity in the US hog sector. American Journal of Agricultural Economics, 2003; 85 (1): 121-133

50. Ukoha O.O, Okoye B.C and Emetu J. Analysis of the Determinants of Total Factor Productivity among Small-Holder Cassava Farmers in Ohafia L.G.A of Abia State, 2010.

51. Govereh J, Jayne T, and Nyoro J. Smallholder commercialization, interlinked markets and food crop productivity: Cross country evidence in eastern and southern Africa, Michigan State University, 1999.

52. Pingai P. From subsistence to commercial production systems: The transformation of Asian agriculture. American journal of agricultural economics, 1997,79: 628-634

53. Osmani A, and Hossain E. Market participation decision of smallholder farmers and it's in Bangladesh. Journal of Economics of Agriculture 1/2015UDC: 631.1.0173:339.3

54. Alelign A. Crop productivity, efficiency and commercialization of smallholder farmers in the Highlands of Eastern Ethiopia in partial fulfillment of the requirements for the degree of doctor of philosophy in agricultural economics Haramaya University, Ethiopia,2017.

55 Addisu G, Degye G, and Adam B . Commercialization of teff: The Case of Smallholder Farmers in Dendi District of Oromia, Central Ethiopia. International Journal of African and Asian Studies, 2019, 56.

56. Birara E, Mezegebu A, Fenta A, and Zemen A. Determinants of Wheat Commercialization among Smallholder Farmers in Debre Elias Woreda, Ethiopia: Advance in agriculture, https://doi.org/,2020,10.1155/2020/2195823

57. Von Braun J, and Kennedy E. Agricultural Commercialization, Economic Development and Nutrition.The Johns Hopkins University Press, Baltimore and London,1994.

58. Strasberg P, Jayne T, Yamano T, Nyoro J, Karanja D, and Strau*--ss, J. Effects of agricultural commercialization on food crop input use and productivity in Kenya: Office of sustainable development; policy synthesis for USAID-Africa Bureau, 1999.

59. Berhanu G, and Moti J. Commercialization of smallholders: Does market orientation translate into market participation? Improving Productivity and Market Success (IPMS) of Ethiopian farmer's project, working paper, 2010; 22: 30 p.

60. Coelli T.J, and G.E. Battesse. A Model of technical efficiency effects in stochastic frontier function for panel data. Journal of Empirical Economics, 1995; 20: 325-332.

61. Meeusen W, and van den Broeck J. Efficiency estimation from Cobb-Douglas production functions with composed error. International Economic Review, 1977; 18: 435-444.

62. KabungaN. S, Dubois T, and Qaim M. Yield effects of tissue culture bananas in Kenya: Accounting for selection Bias and the role of complementary inputs. Journal ofAgricultural Economics, 2012; 63:444–464. https://doi.org/10.1111/j.1477-9552.2012.00337.

63. Shiferaw B, Kassie M, Jaleta M, and Yirga C. Adoption of improved wheat varieties and impacts on household food security in Ethiopia. Food Policy, 2014; 44:272–284. https://doi.org/10.1016/j.foodpol.2013.09.012

64. Ahmed M, Kassaun G, Amreo, T, and Eeden A. The impact of improved maize varieties on farm productivity and wellbeing: Evidence from the east Hararghe zone of Ethiopia. Development Studies Research, 2017; 4, 9–21.

65. Abdulai, A, and Huffman W. The adoption and impact of soil and water conservation technology: An endogenous switching regression application. Land Economics, 90, 26–43. https://doi.org/10.3368/le.90.1.26, 2014.

66.Jaleta M, Kassie M, Marenya P, Yirga C, and Erenstein O. Impact of improved maize adoption on household food security of maize producing smallholder farmers in Ethiopia. Food Sec, 10: 81–93. https://doi.org/10.1007/s12571-017-0759-y, 2018.

67. Khonje M, Manda J, Alene A. D, & Kassie M. Analysis of Adoption and Impacts of Improved Maize Varieties in Eastern Zambia. World Development, 2015; 66: 695- 706.

68. Maddala G.S. Limited dependent and qualitative variables in econometrics. Cambridge, U.K.: Cambridge University Press, 1983.

69. Maddala G.S, and Nelson F.D. Switching regression models with exogenous and endogenous switching. Proceeding of the American Statistical Association (Business andEconomics Section), 1975; pp. 423-426.

70. Caliendo, M, and Kopeinig S. Some practical guidance for the implementation of propensity score matching, Discussion paper No. 1588, University of Cologne,2008.

71. Owusu V, and Awudu A. Nonfarm Employment and Poverty Reduction in Rural Ghana: APropensity-Score Matching Analysis, 2009. DOI 10.22004/ag.econ.51363

72. Nguezet P, Diagne A, Okoruwa V, and Ojohomon V. Impact of improved rice technology on income and poverty among rice farming household in Nigeria; contributed paper prepared for the 25th conference of the Centre for the Studies of African Economics, 2011.

73 Dehejia R, and Wahba S. Propensity score matching methods for non -experimental causalstudies. The Review of Economics and Statistics, 2002; 84(1):151–161.

74. Nicholson CF, Thornton PK, Mohammed L, Muinga RW, Mwamachi DM, Elbasha EH, Staal SJ, and Thorpe W .Smallholder dairy technology in coastal Kenya: An adoption and impact, 1999.

75. Getahun D, Mwangi H, Verkuijl, and Abdishekur W. Assessment of the Adoption of Seed and Fertilizer Packages and the Role of Credit in Smallholder Maize Production, 2000.

76. Mulugeta T, and Hundie B. Impacts of Adoption of Improved Wheat Technologies on Households‟ Food Consumption in Southeastern Ethiopia. Selected Poster prepared for presentation at the International Association of Agricultural Economists (IAAE) Triennial Conference, Foz do Iguaçu, Brazil, 18-24 August, 2012

77. Tolessa Alemu. Adoption and impact of improved agricultural practices and wheat production efficiency of smallholders in arsi zone of Ethiopia. In partial fulfillment of the requirements for the degree of doctor of philosophy in agricultural economics,2014.

78. Grimachew S. Determinants of Adoption of soil and water conservation practices in the environments of Semin Mountains: National Parks Eth. An M.Sc. Thesis Submitted to School of Graduate Studies of Haramaya University, 2005.

79. Million S. Determinants for adoption of physical soil and water conservation measures by smallholder farmers in Ethiopia Doctoral Dissertation, Sokoine University of Agriculture, Morogoro, Tanzania 80.Ana 2009

81. Assefa T, Jema H, Lemma Z, and Dawit A. Commercialization of small holder pulse producer east Gojjam, Ethiopia. Sustainable Agriculture Researc, 20218(4)

82. Habtemariam A. The comparative Influence of Intervening variable in the adoption of Maize and Dairy Farmers in Shashemene and Debrezieit, Ethiopia.PhD Thesis, University of Pretoria, 2004.

83. Astewel T, Yihenew Se. Socio-economic analysis of conditions for adoption of tractor hiring services among smallholder farmers, North-westernEthiopia. Cogent Food and Agriculture https://doi.org/, 2018,10.1080/23311932.2018.1453978

84. Etsehiwot S. The Determinants of Agricultural Technology Adoption and Its’ Impact on teff productivitye in the Case of Amhara and Oromia National Regional State. Degree of Masters of Science in Economics A.A, Ethiopia, 2018.

85. Mesele Z, Abebaw H, and Getamesay B. Determinants of multiple agricultural technology adoption: evidence from rural Amhara region, Ethiopia, Cogent Economics & Finance, 2022; 10:1, DOI: 10.1080/23322039.2022.2058189.

Our reply to reviewers comment and suggestion

We appreciate the constructive and insightful comments of both reviewers for their careful reading of the manuscript and their constructive remarks, on our manuscript entitled ‘‘**Impacts of Agricultural Machine Renting on Cereal Crop Productivity and Commercialization in West Gojjam Zone, Ethiopia**". We are pleased by the comment, input, and clarity raised by both reviewers’. We have taken the reviewers’ comments fully to incorporate, clarify, and improve our manuscript, which will meet the standard of the Plos ONE. The point-by-point response to all the comments is presented below. We highly acknowledge the reviewers for the time and effort spent in reviewing our manuscript.

Reviewer 1

1. The paper's overall structure needs work. There is no research gap in the abstract, and it is rather superficial. The introduction briefly explains the necessity of undertaking this research but does not provide a clear picture of the investigation.

Response: We accepted the comments and clearly rewrite the manuscript on the abstract and introduction.

2. Before the methodology, it should be necessary to conduct a literature review, both theoretical and empirical, and the authors should clarify how the conceptual framework guides the study.

Response: We appreciate and understand the reviewer’s viewpoint. we include theoretical and empirical review and conceptual framework before methodology.

3. The area of the description part is shallow, and it needs more explanation in terms of agricultural machines renting on cereal crop productivity and commercialization in West Gojjam Zone, particularly in your selected districts

Response: We recognized the comments and clearly write the manuscript on the area of the description.

4. To determine the sample size, you should not show the sampling techniques and procedures. Which random sampling techniques were employed to select the sample respondents? What are the benchmarks—192 households adopted and 208 households non-adopted? Please support it with empirical evidence. From Table 1, how did you calculate the sample households?

Response: Thank you for your insightful comment. The entire question answers on sample size determination section and sample household calculation is corrected accordingly your comments.

5. From the data collection methods, you stated that the major data collection methods included observations and visual aids. I have not seen photos in the discussion part. What is important for setting the data collection method?

Response. Thank you for your comments. We avoid the data collection methods of observation and visual aids because it is difficult add on the manuscript. If you want the photo we can attach as a supplementary material.

6**.** The methods of data analysis, particularly the descriptive statistics part, were not clearly explained, and why not consider other alternative methods like inferential statistics (chi-square test and t-test)? The chi-square test and t-test should be used to determine statistical differences and the association between agricultural machine adopters and non-adopters on independent variables.

Response: Accepted and we appreciate the reviewer's comment. We add chi-square test and t-test to determine statistical differences and the association between agricultural machine adopters and non-adopters on independent variables in the descriptive statistics part.

7. Under the econometric model, you should separately explain the theoretical and analytical framework, and then you should specify the model and the conditional expectations, treatment, and heterogeneity effect. Measurement of market participation: It is so shallow; it needs more explanation with supporting theoretical and empirical literature reviews.

Response: We appreciate the reviewer's comments and add supporting theoretical and empirical literature reviews in the revised manuscript.

8. Under equation 4 the authors stated input factors (labour, land, fertilizer seed and herbicide) for production function. In addition to these variables, why not incorporated agricultural machines (like tractor, combiner, and Sheller) as inputs for the production of cereal crops?

Response: We appreciate and understand the reviewer's viewpoint**.** In the production function we did not incorporated agricultural machines (like tractor, combiner, and Sheller) as inputs for the production of cereal crops because these agricultural machines employed by only 192 sample household heads. Moreover, agricultural machines were not fully employed by agricultural operation. For instance, combine harvester used for harvesting, threshing and winnowing of wheat and maize Sheller only used as Sheller of maize. Therefore, we did no incorporated agricultural machines as input.

9. The impacts of agricultural machine rental on productivity and commercialization: The specification of the endogenous switching regression model should not be well organized. So, please specify the ESR model while supporting the theoretical and empirical literature review.

Response: We appreciate the reviewer's comments and we add supporting theoretical and empirical literature reviews about the ESR model in the revised manuscript

10. Under the definition of variables, not only list the independent variables; you should define each independent variable and hypothesize based on the literature review.

Response: Accepted and rewritten accordingly in the revised manuscript

11. Under the discussion part, specifically the descriptive results, the authors simply show the number rather than the implication of the numbers. What are the implications of your results or numbers (percent, mean, standard deviation, min, and max)?

Response: Thank you for your valuable comments and we appreciate the reviewer's viewpoint. We add on the result and discussion part on descriptive statistics sub section the agricultural machine adopter and non- adopter mean difference of independent variable by t-test and chi-square test.

12. Under the discussion part, specifically the impacts of agricultural machine renting on productivity and commercialization, the investigation needs deeper and more detailed discussion of the implications of the findings and how to relate them to the existing literature.

Response : We accepted the comments and clearly rewrite the manuscript on the discussion part.

13. The paper's major contributions and their consequences for practice and policy are not succinctly summarized in the conclusion, which comes off as hurried. It is necessary to have a more thorough conversation about proposed policies.

Response: We appreciate the reviewer's comments and we add the proposed policies on the revised manuscript.

Reviewer #2:

1. What is the contribution of this study to the existing literature?

Response: Accepted and we appreciate the reviewer's questions.

This study provides information on smallholder farmers' agricultural machine renting and its impact on cereal crops productivity and commercialization which helps to draw a clear picture for policymakers involved in the development and dissemination of agricultural mechanization.

2. Adding the description, measurement and expected hypothesis of the independent variable in the methodology section with citation.

Response: Accepted and rewritten accordingly in the revised manuscript

3. If the farmers are used more than two machines, why not use multinomial logit model?

Response: We appreciate and understand the reviewer's viewpoint. We employed ESR model investigate determinants of agricultural machine renting and its impact on productivity simultaneously.in addition the number of each machine adopters were not enough to use multinomial logit model.

4. What are the diagnostic tests that you conducted?

Response: We appreciate you for your understandable and valuable comments. Different model tests such as multicollinearity test, heteroscedasticity test, and omitted variable test have been added in the revised manuscript. The clear descriptions diagnostic tests put on appendix.

5. Support your finding by more empirical evidence.

Response: Thank you for your valuable comment. We have included supporting empirical evidence on the finding with citation dark blue color.

6. Policy implication is too general, so make it specific to the concerned body.

Response: We appreciate the reviewer's comments and we add the proposed policies on the revised manuscript.

7. Generally this paper lacks of consistency

Response: Accepted and we have rearranged the manuscript according to your comments and Plose One guild line.
