# Supplementary material for: Impacts of agricultural machine renting on cereal crop productivity and commercialization in West Gojjam Zone, Ethiopia
Source: PLoS One. 2025 Oct 27;20(10):e0300831. doi: 10.1371/journal.pone.0300831 (PMC12558508; doi:10.1371/journal.pone.0300831)
Supplement: Attachment — Submitted filename: Reviewer report.docx [file pone.0300831.s004.docx]

Impacts of Agricultural Machine Renting on Cereal Crop Productivity and Commercialization in West Gojjam Zone, Ethiopia

**Reviewer comments:**

The paper's overall structure needs work. There is no research gap in the abstract, and it is rather superficial. The introduction briefly explains the necessity of undertaking this research but does not provide a clear picture of the investigation.

Before the methodology, it should be necessary to conduct a literature review, both theoretical and empirical, and the authors should clarify how the conceptual framework guides the study.

The area of the description part is shallow, and it needs more explanation in terms of agricultural machines renting on cereal crop productivity and commercialization in West Gojjam Zone, particularly in your selected districts.

To determine the sample size, you should not show the sampling techniques and procedures. Which random sampling techniques were employed to select the sample respondents? What are the benchmarks—192 households adopted and 208 households non-adopted? Please support it with empirical evidence. From Table 1, how did you calculate the sample households?

From the data collection methods, you stated that **t**he major data collection methods included observations and visual aids. I have not seen photos in the discussion part. What is important for setting the data collection method?

The methods of data analysis, particularly the descriptive statistics part, were not clearly explained, and why not consider other alternative methods like inferential statistics (chi-square test and t-test)? The chi-square test and t-test should be used to determine statistical differences and the association between agricultural machine adopters and non-adopters on independent variables.

Under the econometric model, you should separately explain the theoretical and analytical framework, and then you should specify the model and the conditional expectations, treatment, and heterogeneity effect.

Measurement of market participation: It is so shallow; it needs more explanation with supporting theoretical and empirical literature reviews.

Under equation 4 the authors stated input factors (labour, land, fertilizer seed and herbicide) for production function. In addition to these variables, why not incorporated agricultural machines (like tractor, combiner, and Sheller) as inputs for the production of cereal crops?

**The impacts of agricultural machine rental on productivity and commercialization:** The specification of the endogenous switching regression model should not be well organized. So, please specify the ESR model while supporting the theoretical and empirical literature review**.**

Under the definition of variables**,** not only list the independent variables; you should define each independent variable and hypothesize based on the literature review.

Under the discussion part, specifically the descriptive results, the authors simply show the number rather than the implication of the numbers. What are the implications of your results or numbers (percent, mean, standard deviation, min, and max)?

Under the discussion part, specifically the impacts of agricultural machine renting on productivity and commercialization, the investigation needs deeper and more detailed discussion of the implications of the findings and how to relate them to the existing literature.

The paper's major contributions and their consequences for practice and policy are not succinctly summarized in the conclusion, which comes off as hurried. It is necessary to have a more thorough conversation about proposed policies.
