# Supplementary material for: Impacts of agricultural machine renting on cereal crop productivity and commercialization in West Gojjam Zone, Ethiopia
Source: PLoS One. 2025 Oct 27;20(10):e0300831. doi: 10.1371/journal.pone.0300831 (PMC12558508; doi:10.1371/journal.pone.0300831)
Supplement: Attachment — Submitted filename: reviewrs point by point response plose.docx [file pone.0300831.s005.docx]

**Our reply to reviewers comment and suggestion**

We appreciate the constructive and insightful comments of both reviewers for their careful reading of the manuscript and their constructive remarks, on our manuscript entitled ‘‘**Impacts of Agricultural Machine Renting on Cereal Crop Productivity and Commercialization in West Gojjam Zone, Ethiopia**". We are pleased by the comment, input, and clarity raised by both reviewers’. We have taken the reviewers’ comments fully to incorporate, clarify, and improve our manuscript, which will meet the standard of the Plos ONE. The point-by-point response to all the comments is presented below. We highly acknowledge the reviewers for the time and effort spent in reviewing our manuscript.

**Reviewer 1**
1. The paper's overall structure needs work. There is no research gap in the abstract, and it is rather superficial. The introduction briefly explains the necessity of undertaking this research but does not provide a clear picture of the investigation.

**Response:** We accepted the comments and clearly rewrite the manuscript on the abstract and introduction.

2. Before the methodology, it should be necessary to conduct a literature review, both theoretical and empirical, and the authors should clarify how the conceptual framework guides the study.

**Response:** We appreciate and understand the reviewer’s viewpoint. we include theoretical and empirical review and conceptual framework before methodology.

3. The area of the description part is shallow, and it needs more explanation in terms of agricultural machines renting on cereal crop productivity and commercialization in West Gojjam Zone, particularly in your selected districts

**Response:** We recognized the comments and clearly write the manuscript on the area of the description.

4. To determine the sample size, you should not show the sampling techniques and procedures. Which random sampling techniques were employed to select the sample respondents? What are the benchmarks—192 households adopted and 208 households non-adopted? Please support it with empirical evidence. From Table 1, how did you calculate the sample households?

**Response:** Thank you for your insightful comment. The entire question answers on sample size determination section and sample household calculation is corrected accordingly your comments.

5. From the data collection methods, you stated that the major data collection methods included observations and visual aids. I have not seen photos in the discussion part. What is important for setting the data collection method?

**Response.** Thank you for your comments. We avoid the data collection methods of observation and visual aids because it is difficult add on the manuscript. If you want the photo we can attach as a supplementary material.

6**.** The methods of data analysis, particularly the descriptive statistics part, were not clearly explained, and why not consider other alternative methods like inferential statistics (chi-square test and t-test)? The chi-square test and t-test should be used to determine statistical differences and the association between agricultural machine adopters and non-adopters on independent variables.

**Response:** Accepted and we appreciate the reviewer’s comment. We add chi-square test and t-test to determine statistical differences and the association between agricultural machine adopters and non-adopters on independent variables in the descriptive statistics part.

7. Under the econometric model, you should separately explain the theoretical and analytical framework, and then you should specify the model and the conditional expectations, treatment, and heterogeneity effect. Measurement of market participation: It is so shallow; it needs more explanation with supporting theoretical and empirical literature reviews.

**Response:** We appreciate the reviewer’s comments and add supporting theoretical and empirical literature reviews in the revised manuscript.

8. Under equation 4 the authors stated input factors (labour, land, fertilizer seed and herbicide) for production function. In addition to these variables, why not incorporated agricultural machines (like tractor, combiner, and Sheller) as inputs for the production of cereal crops?

**Response:** We appreciate and understand the reviewer’s viewpoint**.** In the production function we did not incorporated agricultural machines (like tractor, combiner, and Sheller) as inputs for the production of cereal crops because these agricultural machines employed by only 192 sample household heads. Moreover, agricultural machines were not fully employed by agricultural operation. For instance, combine harvester used for harvesting, threshing and winnowing of wheat and maize Sheller only used as Sheller of maize. Therefore, we did no incorporated agricultural machines as input.

9. The impacts of agricultural machine rental on productivity and commercialization: The specification of the endogenous switching regression model should not be well organized. So, please specify the ESR model while supporting the theoretical and empirical literature review.

**Response:** We appreciate the reviewer’s comments and we add supporting theoretical and empirical literature reviews about the ESR model in the revised manuscript

10. Under the definition of variables, not only list the independent variables; you should define each independent variable and hypothesize based on the literature review.

**Response:** Accepted and rewritten accordingly in the revised manuscript

11. Under the discussion part, specifically the descriptive results, the authors simply show the number rather than the implication of the numbers. What are the implications of your results or numbers (percent, mean, standard deviation, min, and max)?

**Response:** Thank you for your valuable comments and we appreciate the reviewer’s viewpoint. We add on the result and discussion part on descriptive statistics sub section the agricultural machine adopter and non- adopter mean difference of independent variable by t-test and chi-square test.

12. Under the discussion part, specifically the impacts of agricultural machine renting on productivity and commercialization, the investigation needs deeper and more detailed discussion of the implications of the findings and how to relate them to the existing literature.

**Response :**We accepted the comments and clearly rewrite the manuscript on the discussion part.

13. The paper's major contributions and their consequences for practice and policy are not succinctly summarized in the conclusion, which comes off as hurried. It is necessary to have a more thorough conversation about proposed policies.

**Response:** We appreciate the reviewer’s comments and we add the proposed policies on the revised manuscript.

**Reviewer #2:**

1. What is the contribution of this study to the existing literature?

**Response:** Accepted and we appreciate the reviewer’s questions.

This study provides information on smallholder farmers’ agricultural machine renting and its impact on cereal crops productivity and commercialization which helps to draw a clear picture for policymakers involved in the development and dissemination of agricultural mechanization**.**

2. Adding the description, measurement and expected hypothesis of the independent variable in the methodology section with citation.

**Response**: Accepted and rewritten accordingly in the revised manuscript

3. If the farmers are used more than two machines, why not use multinomial logit model?

**Response:** We appreciate and understand the reviewer’s viewpoint. We employed ESR model investigate determinants of agricultural machine renting and its impact on productivity simultaneously.in addition the number of each machine adopters were not enough to use multinomial logit model.

4. What are the diagnostic tests that you conducted?

**Response:** We appreciate you for your understandable and valuable comments. Different model tests such as multicollinearity test, heteroscedasticity test, and omitted variable test have been added in the revised manuscript. The clear descriptions diagnostic tests put on appendix.

5. Support your finding by more empirical evidence.

**Response:** Thank you for your valuable comment. We have included supporting empirical evidence on the finding with citation dark blue color.

6. Policy implication is too general, so make it specific to the concerned body.

**Response:** We appreciate the reviewer’s comments and we add the proposed policies on the revised manuscript.

7. Generally this paper lacks of consistency

Response: Accepted and we have rearranged the manuscript according to your comments and Plose One guild line.
