# Supplementary material for: Impacts of agricultural machine renting on cereal crop productivity and commercialization in West Gojjam Zone, Ethiopia
Source: PLoS One. 2025 Oct 27;20(10):e0300831. doi: 10.1371/journal.pone.0300831 (PMC12558508; doi:10.1371/journal.pone.0300831)
Supplement: Attachment — Submitted filename: reviewer response.docx [file pone.0300831.s007.docx]

**Our reply to reviewers comment and suggestion**

We appreciate the constructive and insightful comments of reviewer for their careful reading of the manuscript and their constructive remarks, on our manuscript entitled ‘‘Impacts of Agricultural Machine Renting on Cereal Crop Productivity and Commercialization in West Gojjam Zone, Ethiopia". We are pleased by the comment, input, and clarity raised by reviewer. We have taken the reviewer comment fully to incorporate, clarify, and improve our manuscript, which will meet the standard of the Plos ONE. The point-by-point response to all the comments is presented below. We highly acknowledge the reviewer for the time and effort spent in reviewing our manuscript. Accordingly, changes made in the revised manuscript are highlighted with green color.

1. Show the national average index of agricultural mechanization in number?

**Response:** Accepted and we appreciate the reviewer’s comment. We read different journals and other materials about agricultural mechanization status in Ethiopia even though, they describe only in comparison. Therefore, we add some explanation in number about mechanization index of Ethiopian region in the revised manuscript.

2. Justify the theory of Boserup about agricultural mechanization?

**Response:** We appreciate the reviewer’s comments and add clarification about the theory of Boserup in the revised manuscript.

3. What are the bench mark of 192 households adopted and 208 households non-adopted?

**Response**: Thank you for your insightful comment. We recognized the comments and clearly write the bench mark of adopter and non-adopter in the revised manuscript .in table 1. Additionally, households were selected in each kebele proportionally.

4. Comment from the manuscript grammatical and spelling error.

**Response:** accepted and we appreciate the critical view of the reviewer’s typos error. We corrected every error in the revised manuscript according to your comment.

5. . There was one citation appearing in the narrative section but not in the reference.

Response: Thank you for your valuable comment. We have included the in reference section.

6. Add the source of technical efficiency formula.

Response; Accepted and we appreciate the reviewer’s comment. We add citation for technical efficiency formula in the revised manuscript..
